# Since Albert and Whetten: the dissemination of Albert and Whetten’s conceptualization of organizational identity

**DOI:** 10.1007/s11301-022-00311-7

**Published:** 2022-12-02

**Authors:** Karin Knorr, Franziska Hein-Pensel

**Affiliations:** 1 Faculty of Business Administration and Economics, Department Management, Paderborn University, Paderborn, Germany; 2 Department of Economic Sciences and Media, TU Ilmenau, Ilmenau, Germany

**Keywords:** Organizational identity, Systematic literature review, Mixed methods approach, Organization theory

## Abstract

Since the seminal work of Albert and Whetten, the organizational identity concept has become ubiquitous and highly relevant in various fields. This study systematically reviews how Albert and Whetten’s concept of organizational identity has been disseminated in different research areas. It employs quantitative (topic modeling) and qualitative text analysis, as well as a network analysis to examine a sample of 1,041 papers published between 1985 and mid-2022 that cite Albert and Whetten’s seminal work. Using this method of systematic literature analysis, the current study investigates the criteria of the basic definition and hypotheses mentioned in their work that contribute to its increasing significance, and those with the potential to become substantial aspects of future organizational identity research. Accordingly, Albert and Whetten’s conceptualization of organizational identity is often partially adopted in the literature. Thus, this study contributes to organizational identity research by unveiling further research questions on the evolving character of organizational identity, research methodology, and quantitative operationalization, on the basis of Albert and Whetten’s organizational identity conceptualization.

**Supplementary information:**

The online version contains supplementary material available at 10.1007/s11301-022-00311-7.

## Introduction

Since Albert and Whetten’s (1985) seminal work, research on organizational identity has proliferated (He and Brown 2013), and inspired over 50,000 articles.^1^ Organizational identity is defined as what is “central, distinctive and enduring” about an organization (Albert and Whetten 1985, p. 265). When organizations face crises or difficult decisions, identity becomes highly relevant “because of its powerful sense-making, direction-setting and motivating roles” (Annosi et al. 2017, p. 620). Pugliese et al. (2022) show that the more organizations embody the prototypical characteristics of their market sector in their organizational identity, the lower the negative effects of a crisis on perceived organizational performance. In response to the COVID-19 crisis, some organizations also adjust the formulations of their mission to emphasize their sense of identity and commitment to communities (Ortiz 2022). Doing so demonstrates that active identity management and adaptation in real time enables organizations to make a better overall impression on potential applicants. It also ensures that organizations have larger and higher quality applicant pools, and current employees have stronger retention and loyalty to the organization (Bankins and Waterhouse 2019). The continuous questioning and adaptation of organizational identity also plays a major role in the introduction of new technologies into the organization. In a time of technological transformation, Tripsas (2009) describes the requirement for the adaptation of organizational identity by both organizational members and external stakeholders through a fundamental change in the belief of what the organization represents. This differentiation between external and internal identity is very common in organizational theory. Thereby, internal identity can be defined as organizational identity, and external identity represents how outside audiences perceive the organization.

A structuring of research works from the last decades per the research focus on organizational identity reveals two major objectives. The first is the creation of organizational identity via claims, such as according to the history (Barnes and Newton 2018; Foster et al. 2011; Oertel and Thommes 2018); founder (Basque and Langley 2018); organizational members (Anteby and Molnár 2012; Bojovic et al. 2020); and products, artifacts, and practices (Watkiss and Glynn 2016). The second involves ascertaining the intentional and unintentional effects of organizational identity on elements, such as employee commitment and behavior (Matherne et al. 2017), fostering of organizational interests (Ashforth and Mael 1996), and the strength of employee identification with the organization (Dutton et al. 1994). Central to studies in both literature streams are three key questions for the conceptualization of organizational identity: Who are we? What are we doing? What do we believe? (Lerpold et al. 2012).

Accordingly, several studies have characterized Albert and Whetten’s (1985) concept as an essential contribution (Gioia et al. 2000; He and Brown 2013) and foundation for further research. For instance, Corley et al. (2006, p. 86) define Albert and Whetten’s (1985) paper as an “influential work” that has “launched a wave of research and theory that continues to the present.” Further, Albert et al. (2000, p. 13) describe it as a “root construct” for organizational research as they develop over 30 hypotheses that highlight further investigation.

Previous literature reviews (e.g., He and Brown 2013; Ravasi and Canato 2013) refer to organizational identity research in general; none of them explain which hypotheses of Albert and Whetten’s (1985) seminal paper have been considered in research, which have been left unconsidered, and which offer possible further research avenues. Therefore, it is vital to appreciate this “root” and identify the parts of their concept that have spread to better understand the organizational identity field that emerged from their research. Hence, this study’s contribution is twofold. First, it provides an in-depth analysis of the dissemination of Albert and Whetten’s (1985) seminal paper. Particularly, we analyze how the three criteria of central character, distinctiveness, and temporal continuity of Albert and Whetten’s (1985) basic definition have been addressed in research. Moreover, it provides an overview of which hypotheses and their thematic foci (i.e., perception of organizational identity, organizational identity during life-cycle events, mono and dual identities, and comparison of organizational identity in different organization types), as well as the metaphor analysis as a methodological approach, mentioned therein have been implemented by an analysis of their dissemination in various research fields. Further, we investigate the interrelationship among different research fields and authors by a demonstration of how studies from various fields are connected. The systematic analysis generates an in-depth understanding of how authors and research fields reference Albert and Whetten’s (1985) conceptualization of organizational identity in different contexts, which demonstrates the versatility of organizational identity research. Through a qualitative content analysis of recurring topics and a keyword-in-context search, we group studies and their content into five *aggregated dimensions*, each of which describe a different focus derived from Albert and Whetten’s (1985) seminal paper. A subsequent contextualization of the content analysis with the author network data reveals that six subnetworks of authors can be categorized.

Second, this study offers suggestions for future research in three different research areas, based on the seminal work of Albert and Whetten (1985). It highlights which of their hypotheses and how their methodological approach have been forgotten via a systematic analysis of organizational identity research. The resulting transparency will allow future researchers to reflect on their perception of organizational identity based on prior studies and better integrate their work into existing literature. Our findings clearly illustrate that the three basic criteria that an organizational identity has to satisfy—central character, distinctiveness, and temporal continuity—are frequently used to describe and characterize the phenomena of organizational identity. Differences can be observed with regard to the use of the groups of hypotheses. While the hypotheses that concern the perception of organizational identity, especially internal identity, are frequently referenced, the groups of hypotheses that concern the organizational life-cycle or the comparison of organization types have found little resonance. The same applies to the methodological approach suggested by Albert and Whetten (1985). Based on these results, future research avenues are derived.

The next section addresses Albert and Whetten’s (1985) paper and explains its central aspects as a basis for this study. Following the presentation of the data and analysis methods, we discuss the results and note future research opportunities.

## Albert and Whetten’s concept of organizational identity research

Albert and Whetten (1985) underline that the concept of organizational identity has two uses. First, as a scientific concept, it is used to define and characterize organizational aspects. Second, organizations refer to the concept to characterize certain aspects of themselves. Moreover, Albert and Whetten (1985) state their basic definition of organizational identity using three criteria: central character, distinctiveness, and temporal continuity, and emphasize that these need to be satisfied to obtain an adequate statement of the organizational identity. The literature primarily references these criteria, while largely ignoring their 32 hypotheses^2^ of the organizational identity concept (Foreman and Whetten 2016). In terms of content, these hypotheses (Table 1) can be assigned to four groups, with some of the hypotheses falling into more than one group. The first group (Perception of organizational identity) includes hypotheses that relate to the perception of organizational identity, the consequences of a difference between internal and external perceptions, and their different constructions (H1–H3). The second group (Mono and dual identities) summarizes hypotheses that relate to organizations that have more than just one organizational identity (H4–H6). Regarding dual identity, they further distinguish between ideographic and holographic identities. Unlike ideographic dual identity, where different organization groups construct various organizational identities, a holographic dual identity reflects identities across organization groups, which they share simultaneously (Albert and Whetten 1985). Thus, leaders of organizations with dual or multiple identities should personify and support them all instead of a single identity that they consider to be the most suitable (Albert and Whetten 1985). The personification of only one identity can induce a lack of confidence in their leadership and reflect a lack of legitimacy, especially in times of crisis. Moreover, although Albert and Whetten’s (1985) definition of organizational identity includes the criteria of temporal continuity, the researchers also emphasize that an organization’s identity is unstable and can evolve over time. Thus, they conclude that organizational identity adapts to the organizational life-cycle that results in the third group of hypotheses (H7–H12). The final group of hypotheses (Comparison of organization types) focuses on the identity formation of normative and utilitarian organizations. Thus, hypotheses that can be assigned to this group draw a comparison between the identity formation of these two types of organizations (H22, H27, H29, H30, H33). Moreover, metaphor analysis is suggested as a methodological approach. Albert and Whetten (1985, p. 280) highlight that an “extended metaphor analysis” should be particularly conducted “if there is no comprehensive theory to predict how many identities an organization has, or how the dimensions of each are to be defined.” Thus, a characterization rather than a definition of identity should be conducted (Albert and Whetten 1985). They employed metaphor analysis to compare universities and religious institutions.

Since the concept of organizational identity has in the past often been related to other concepts (such as image, reputation, or legitimacy) that underline the complexity of the organizational identity concept, some researchers identify how these concepts differ from or are related to organizational identity (e.g., Brown et al. 2006; Hatch and Schultz 2002; Whetten 2006). Ravasi (2016) states that the two terms, organizational image and organizational reputation, are used interchangeably to refer to the stakeholder’s external perception of the organization. Hatch and Schultz (2002) emphasize that image can be defined as the external definition of organizational identity. In contrast, organizational culture refers to the internal definition of organizational identity. Thus, organizational identity mirrors the image of the stakeholders, and leaves an impression on them. At the same time, organizational identity is anchored in the organization’s cultural patterns while culture makes itself known from the identity claims (Hatch and Schultz 2002). Thus, both organizational image and culture influence the organizational identity and vice versa, where organizational identity serves as a link between image and culture (Hatch and Schultz 2002; Ravasi 2016). Aligning and realigning the organizational image, identity, and culture is vital for organizations to successfully manage identity-threatening events (Ravasi and Tripsas 2020). Additionally, organizational identity is associated with an organization’s legitimacy; organizations strive to construct an identity in order to build and strengthen their legitimacy (King and Whetten 2008). In this regard, the three criteria of the central, enduring, and distinctive nature of organizational identity are the primary contributors that make the organization legitimate to other similar ones (Whetten and Mackey 2002).

This study provides a general overview of Albert and Whetten’s (1985) contribution in the field of organizational identity research. We conduct a systematic analysis to investigate changes in the adaptation of Albert and Whetten’s (1985) concept over the years, illustrate author networks and their groups of hypotheses, and consider the journals that published studies that cite Albert and Whetten (1985). Further, we investigate the adaptation of the criteria of the basic definition and groups of hypotheses emergent from this analysis in the business literature and other fields to estimate their influence on research, and allow for a deeper understanding of the same in organizational research and other research fields. The analysis process is summarized in the following research questions:

*Which criteria of the basic definition and groups of hypotheses of* Albert and Whetten’s (1985*) seminal study have been disseminated in subsequent research? (RQ1a)*

*Which criteria of the basic definition and groups of hypotheses, journals, and author networks play a central role in the adaptation of* Albert and Whetten’s (1985*) concept within and across fields outside of the business literature over time? What are the differences and similarities? (RQ1b)*

Answers to these questions reveal which parts of Albert and Whetten’s (1985) original concept have been forgotten and may be significant to future research, or whether they require revision. Therefore, we investigate a final research question in a separate chapter on future research questions.

*Which criteria of the basic definition and groups of hypotheses of* Albert and Whetten’s (1985*) organizational identity construct have been neglected in the existing literature, and how can they complement future research? (RQ2)*

**Table 1:** Albert and Whetten’s (1985*) Hypotheses*

Hypothesis	Group of hypotheses	Key statements	Pages
**H1**	Organizational identity perception	The more the perception within an organization differs from the outside perception, the more the organization’s health is threatened.	269
**H2**	Organizational identity perception	The identity that is publicly presented will typically be more positive than the identity that is internally perceived.	269
**H3**	Organizational identity perception	The identity that is publicly presented will typically be more monolithic than the identity that is internally perceived.	269
**H4**	Mono and dual identities	According to taxonomic traditions, most organizations have a single identity. Alternatively, many, if not most, organizations are hybrids comprising multiple types.	270
**H5**	Mono and dual identities (ideographic organizations)	Ideographic organizations possess greater specializations and purer identity types. Thus, members are better prepared to observe different environmental conditions and provide appropriate recommendations for adaptive organizational modifications.	272
**H6**	Mono and dual identities (holographic organizations)	Holographic organizations have less diversity to draw upon to formulate a “correct” action plan. As soon as a plan is proposed, leaders can draw on common characteristics in a unit to build consensus.	272
**H7**	Organizational life-cycle events (formation)	As the organization forms and defines its niche exactly, questions about goals, means, and technology (“who are we as an organization”) will emerge.	274
**H8**	Organizational life-cycle events (loss)	The loss of an identity-forming element (e.g., founder of a young organization leaves prematurely) will cause a soul-searching phase regarding the organization’s identity in the effort to find a suitable successor.	274
**H9**	Organizational life-cycle events (raison d’être)	An organization has to work out its raison d’être. Thus, organizations consider various alternatives, including those that may change their central focus and purpose of existing.	274
**H10**	Organizational life-cycle events (growth)	With extremely high organizational growth, the organization reflects on issues of identity.	274
**H11**	Organizational life-cycle events (collective status)	The change in “collective status” in an organization (e.g., “threat of a hostile takeover” and “a carefully planned merger”) is likely to trigger vigorous debates about the mission, values, and identity of the organization.	274
**H12**	Organizational life-cycle events (retrenchment)	When organizational growth is slow, the issue of organizational identity is most acute. Slow growth induces additional goals, missions, and objectives. In addition, cutbacks require the definition of organizational identity as they demand the use of prioritizing budgets.	274–275
**H13**	Mono and dual identities, Organizational life-cycle events	As organizations expand, there is a general tendency for organizations with a single identity to adopt a dual identity.	276
**H14**	Mono and dual identities (environment), Organizational life-cycle events	Suppose the environment in which an organization is embedded becomes increasingly complex over time. In that case, an organization with a dual identity must have adaptive advantages over an organization with a single identity. Organizations acquire dual identities over time to take advantage of opportunities presented by an increasingly complex and changing environment while coping with increasing environmental constraints and regulations.	276
**H15**	Mono and dual identities, Organizational life-cycle events	Some organizations adopt multiple identities and become the repository for all things other organizations do not want. Organizations with relatively little control over the scope of their mission, are expected to have a common path to duality.	276–277
**H16**	Mono and dual identities (identity divestiture), Organizational life-cycle events	Organizations tend to become attached to what they have been and rarely replace old identity traits with new ones. If this assumption were true, it would mean that drifting toward duality is necessary for a process of identity expansion.	277
**H17**	Mono and dual identities (success), Organizational life-cycle events	It is common for organizations that are successful pursuing a single identity to enter a second sphere of activity because of their success in the first sphere.	277
**H18**	Mono and dual identities, Organizational life-cycle events (identity shifts)	Organizations that change their identity throughout their life-cycle and, after a brief period of trying out a new identity, return to their earlier ideological roots is an avenue for investigation. These shifts may be intentional (taking advantage of new opportunities) but are more likely to occur because of identity drift, especially in young organizations.	277
**H19**	Organizational life-cycle events, Comparison of organization types (identity shifts)	A normative organization (e.g., church) expanding or over time, will look more like a utilitarian organization (e.g., business).	278
**H20**	Organizational life-cycle events, Comparison of organization types (identity shifts)	Over time, a growing utilitarian organization (e.g., growing business) is less likely to look like a normative organization than an expanding normative organization (e.g., expanding church) is to look like a business.	278
**H21**	**NA**	No reference to H21 is made in the paper’s body of text.	
**H22**	Comparison of organization types (threats)	A normative organization under attack will prepare a utilitarian defense, just like a threatened utilitarian organization will defend itself on normative grounds.	279–280
**H23**	Organizational life-cycle events (stable identity), Comparison of organization types	Single normative identities persisting throughout the life-cycles of the organization (Path 1) occur infrequently.	280
**H24**	Organizational life-cycle events (identity shifts), Mono and dual identities, Comparison of organization types	Normative organizations that change their identity throughout their life-cycle events and end up with a single identity (Paths 2 and 3) occur less frequently than normative organizations that end up with dual identities (Paths 4 and 5).	280
**H25**	Organizational life-cycle events, Comparison of organization types (cuts)	A normative organization withstands profound across-the-board cuts better than a utilitarian one. Normative organizations typically require a long socialization period, reinforcing a sense of cohesion and shared belief.	287
**H26**	Perception of organizational identity, Comparison of organization types (decision-making)	What is preserved in a normative organization likely differs from that in a utilitarian organization simply because the principles underpinning such decision-making are quite different (normative organizations tradition; utilitarian organization cost-effectiveness).	287
**H27**	Comparison of organization types (leadership)	Normative and utilitarian organizations have different leadership patterns.	288
**H28**	Perception of organizational identity, Organizational life-cycle events, Comparison of organization types (organizational members)	A leader of a normative organization expects that disclosure of an external threat binds members more closely to the organization and mobilizes them to defend it. The opposite is true for utilitarian organizations. Moreover, normative organizations might have a greater tendency to view themselves as unique than utilitarian organizations. Thus, individuals might be particularly reluctant to join because they perceive that they have nowhere else to go.	288
**H29**	Comparison of organization types (organizational learning)	It is expected that utilitarian organizations are more likely to seek management advice from outsiders than normative organizations. Normative organizations are more likely to believe that only an insider can understand the organization’s workings.	288
**H30**	Comparison of organization types (scarcity)	Normative organizations are often prevented by law and ideology, from accumulating purely economic wealth in case of future scarcity. Thus, normative organizations are economically vulnerable. That is, unless they can hide their wealth or redefine its meaning.	289
**H31**	Organizational life-cycle events, Comparison of organization types (merger)	A merger is a common solution to reduce costs. While merging two entities is always fraught with challenges, such mergers are much more difficult when what is to be merged represents different beliefs.	289
**H32**	Perception of organizational identity, Comparison of organization types (marketing)	Utilitarian organizations pursue advertising and marketing, while normative organizations pursue missionary activities. Normative organizations sometimes reject advertising because it is demeaning or undignified, based on the claim that if something of intrinsic value can be shown to have instrumental value, its intrinsic value is diminished. Thus, the normative core of a university is ambivalent about “selling” the university to an external environment.	289–290
**H33**	Comparison of organization types, Normative and utilitarian organizations (priorities)	Normative and utilitarian organizations can be expected to arrive at different priorities in response to scarcity and differ in how judgments are formulated.	290

## Methods

### Access and sample

The dataset for this study offers a unique view of organizational identity as it includes all papers in English published between 1985 and mid-2022 that reference Albert and Whetten (1985), are listed in the Web of Science online databank on the date of data collection (July 11, 2022), and are categorized as an article by the Web of Science. Since this study focuses on how seminal ideas on organizational identity spread, data collection is not restricted to a list of specific journals and research fields.

The Web of Science lists 1,116 papers for the described criteria. Some of the papers are contributions to books (45) or dissertations (1), which we eliminated for better comparability in subsequent analysis steps; 29 papers were inaccessible, which left us with a dataset of 1,041 English papers (Appendix 1 provides the complete list). Further, we noted every paper’s citation rate, the journal it was submitted to, and the corresponding research field per the Web of Science categories.

This study investigates the adaptation of Albert and Whetten’s (1985) concept within and across fields over time via quantitative and qualitative text analysis with relevant metadata. We employ the full-text corpus (instead of only abstracts) to compare the research themes and explicit text fragments where Albert and Whetten (1985) are referenced, which thus improves the quality of the quantitative text analysis by up to 40% (Syed and Spruit 2017).

### Analytical approach

**Fig. 1:**
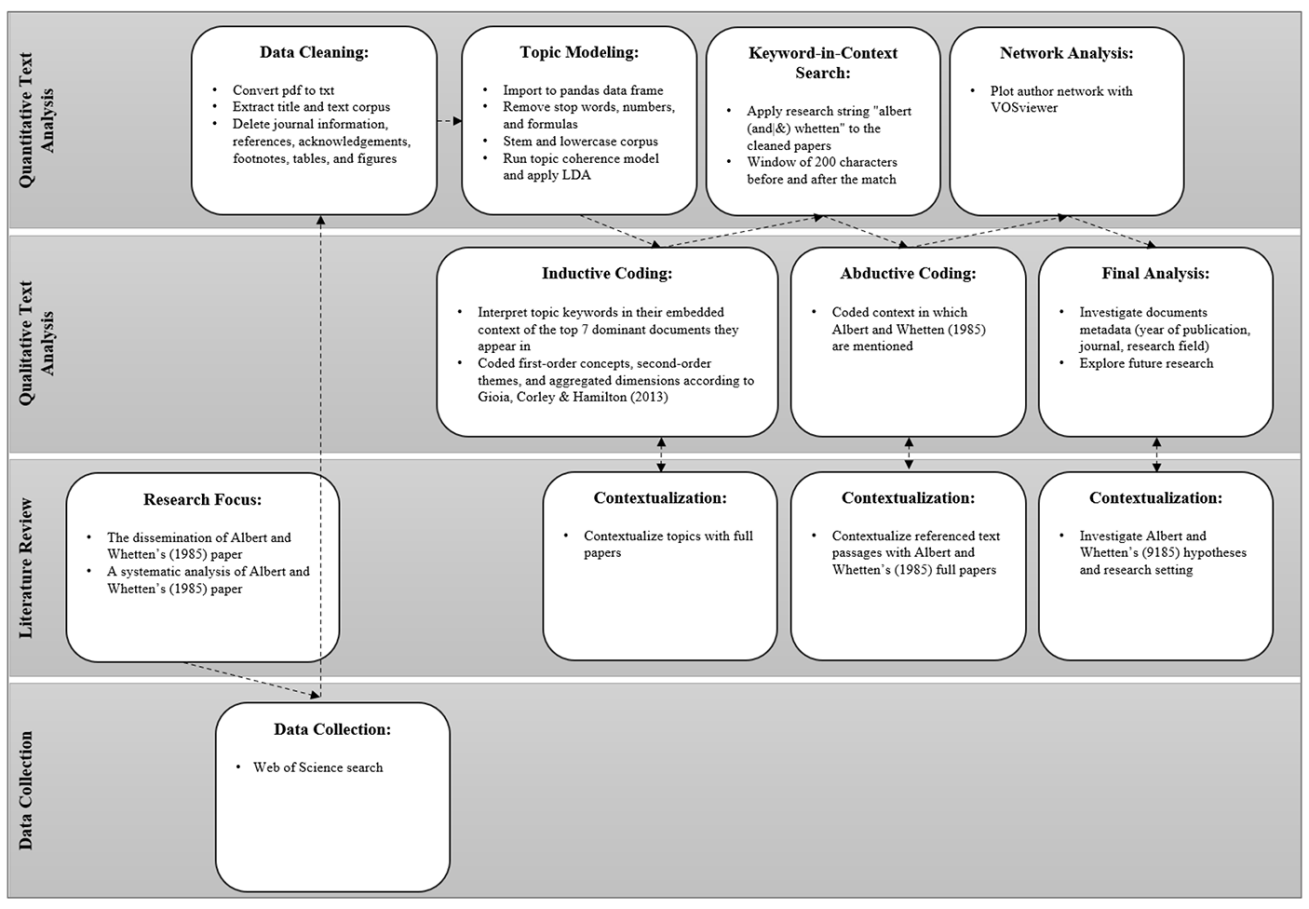
Analytical Approach

To analyze the dissemination of the basic criteria, the hypotheses and the suggested metaphor analysis by Albert and Whetten (1985) and to expose those parts that gained less attention or have even been forgotten, we provide a systematic overview by the combination of quantitative and qualitative text analysis (Fig. 1). In the first step, we apply *latent Dirichlet allocation* (LDA), a machine-learning algorithm for unsupervised topic modeling, to our text corpus. LDA generates a probability distribution over topics for each document in the corpus. Similar patterns of these distributions indicate a semantic similarity between documents and reveal hidden structures in the content, which allow us to analyze them in depth. The topic with the highest probability (dominant topic) provides a good indication of the primary content of a document, given each topic’s probabilistic association with a set of keywords. Overall, topic modeling is an exploratory technique for unveiling information from large-scale textual data (DiMaggio et al. 2013). It provides a first impression on how scholars discuss organizational identity and their focus of research by the automatic creation of topics based on word occurrences and frequencies (DiMaggio et al. 2013). From the mathematical notion of topic coherence (Röder et al. 2015), we derived an optimal number of 18 topics for our dataset (Appendix 2 provides detailed specifications).

Further, to develop a complete picture of how the article by Albert and Whetten (1985) influenced scholars in organizational identity research, we complement the initially generated quantitative results of topic modeling by qualitative content analysis. Hence, we offer an in-depth analysis of the dissemination of Albert and Whetten’s (1985) concept to construct organizational identity at a much larger scale than through a mere qualitative approach (Croidieu and Kim 2018; Shimizu 2017).

First, using Gioia et al.’s (2013) inductive coding approach, we interpret the topic keywords in the embedded context of the respective top five dominant documents, which allows for the association of each topic with multiple thematic labels (*first-order concepts*) to contextualize them. Zooming out of the articles again and comparing the created *first-order concepts* help generalize them into shared themes (*second-order themes*). Independent coding results are then compared, and differences are discussed until a consensus of the topics is derived. Finally, we alternate between the *first-order codes, second-order themes*, and full documents to inductively derive more *aggregated dimensions* (Table 2).

**Table 2:** Inductive Coding arranged by Aggregated Dimensions. (In addition to the 14 topics that we were able to assign to the five *aggregated dimensions*, our analysis revealed four further topics. These are only mentioned as the dominant topic in a total of two papers and, therefore, are not a good representation of the papers in the data sample and not considered further in the analysis.)

Topic	Illustrative topics vocabularies	First-order concepts	Second-order themes	Aggregated dimensions
**0**	*firm, famili, busi, green, resourc, ventur, industri, innov, perform, environment*	• Family business • Lone founder firms • Corporate governance • Growth • Merger & acquisition	Family firm	Organizational fields
**1**	*univers, student, school, educ, institut, academ, program, colleg, faculti, higher*	• Higher education • School institutional identities • Primary school • Business schools	Educational institutions	
**11**	*foundat, nonprofit, organ, communiti, activist, volunt, public, mission, fund, campaign*	• Nonprofit organizations • Philanthropic organizations • Religious nonprofit organizations • Religious congregations • Funding	Nonprofit organizations	
**7**	*manag, chang, process, collabor, new, develop, innov, partner, knowledg, practic*	• Organizational change • Organizational tension/demands • Organizational challenge • Enterprise resource planning • Potential managerial pathways • Cognitive framing perspective • Paradox research	Organizational change	Organizational development
**10**	*ident, organiz, organ, chang, manag, imag, strategi, studi, strateg, extern*	• Strategic change • Organizational change • External presentation of organizational identity • Organizational identity • Image • Strategic projection • Identity-strategy	Organizational identity management	
**6**	*employe, organiz, organ, studi, leadership, valu, identif, behavior, effect, relationship*	• Organizational identification • Ethical leadership • Servant leadership • Human resource practices • Ethical organizational culture • Work–family • Turnover intention • Work engagement • Employee’s (psychological) well-being	Leadership and workplace conditions	Strategic management
**9**	*csr, organis, stakehold, corpor, respons, orient, activ, authent, social, relationship*	• Corporate social responsibility • Stakeholder management • Attachment • Authenticity • Company-favoring outcomes	Corporate social responsibility	
**13**	*brand, corpor, compani, custom, market, consum, product, reput, communic, hotel*	• Corporate identity • Corporate brand • Corporate reputation • Corporate communication • Marketing • Brand commitment	Corporate branding	
**15**	*social, organ, institut, valu, public, servic, agenc, legitimaci, mission, govern*	• Social businesses • Nonprofit agencies • Benefit corporation • Multiple organizational forms • Public relations • Social expectations • Strategies	Corporate governance	
**16**	*ident, identif, organ, member, group, individu, organiz, social, may, like*	• Organizational identity • Group identity • Work identity • Organizational perceptions • Organizational identification • Social identity theory	Organizational identification	Organizational theory research
**17**	*ident, organ, organiz, use, claim, mean, collect, form, construct, understand*	• Organizational identity use • Organizational identity construction • Collective understanding • Organizational theorizing	Organizational identity conceptualization	
**2**	*effect, use, variabl, model, measur, result, studi, differ, signific, network*	• Bivariate correlation • Cluster analysis • Logistic regression • Ordinary least squares	Quantitative method	Methodology/Research approach
**4**	*research, studi, ident, collect, data, work, develop, interview, find, field*	• Qualitative and quantitative studies • Mixed-methods • Citation analysis • Bibliometric studies • Co-word analysis • Scholarly network/network maps • Survey • Questionnaire • Field study	Mixed methods	
**5**	*work, one, peopl, interview, new, also, time, case, would, manag*	• Interviews • Training reports • Semi-structured interviews • Ethnography	Explorative/Qualitative method	

We employ a keyword-in-context analysis by searching every citation of Albert and Whetten (1985) in the dataset to investigate RQ1a. Therefore, we set our keyword string to “albert (and|&) whetten”^3^ and defined our search to ignore upper and lower cases. The output is defined by a window of 200 (200) single elements on the right-hand (left-hand) side of the keyword. Overall, we found 2,011 relevant matches for the regular expression. We independently coded 200 matches per the context in which Albert and Whetten (1985) are referenced. After discussing and combining the results of the independent coding process, we coded the remaining matches.

Further, the discovered *aggregated dimensions* can be set to the document’s metadata, such as the publication year, journal, and co-authorship, which helps to specify their dissemination. Hence, we can use the publication year and journal to additionally answer RQ1b.^4^ The connection of the results by the construction of a bibliometric network allows for the analysis of different groups of authors who have shaped the thematic scope of the dissemination of Albert and Whetten’s (1985) concept (van Eck and Waltman 2010). Additionally, we illustrate the relationships between the authors (Kraus et al. 2014). Thus, to enhance our understanding of how author networks develop, we combine the content analysis findings with bibliographic data (Zupic and Čater 2015) to illustrate which author networks play a central role in adapting Albert and Whetten’s (1985) concept and investigate how they use it (RQ1b). Assigning different research fields to the authors and their author networks helps differentiate networks based on their related research fields. Thus, we can detect interdisciplinary networks and the dissemination of Albert and Whetten’s (1985) concept across the fields. Therefore, this study sheds light on the most influential authors across different fields and how they affect each other (RQ1b).

We used the open-source tool VOSviewer to visualize the network of authors and set the minimum number of nodes to two (van Eck and Waltman 2010). Hence, an author must be connected via co-authorship with at least two other scholars in our dataset to appear in the network. Finally, we compare the keyword-in-context analysis results and the different references to Albert and Whetten (1985) to address RQ2.

## Findings

### Organizational identity research referencing Albert and Whetten (1985)

#### Investigating topics

The quantitative text analysis revealed 18 significantly different topics. We used inductive manual coding (Croidieu and Kim 2018; Shimizu 2017) for the topic modeling results to find five distinct *aggregated dimensions*^5^ of research on Albert and Whetten (1985) (Table 2). Most topics (topics: 6, 9, 13, 15) are categorized as *strategic management*, including all *second-order themes* that share a general thematic focus on corporate governance and leadership, and how to use them strategically to meet environmental expectations, foster employee well-being, and achieve business-favorable outcomes. Another aggregated dimension is *organizational fields* (topics: 0, 1, 11), which considers all *second-order themes* that focus on specific types of businesses, such as family and nonprofit businesses or educational institutions. Furthermore, the aggregated dimension of *organizational development* (topics: 7, 10) is defined, which on the one hand includes the *second-order theme* that refers to organizational changes triggered by demands and obstacles that the organization has to overcome; therefore, new resource planning or management paths have to be considered. On the other hand, the *second-order theme* of organizational identity management is assigned to this aggregate dimension, since papers of this *second-order theme* concentrate on the organization’s development through the strategic and conscious change of the (projected) organizational identity. The aggregated dimension of *organizational theory research* (topics: 16, 17), focuses on the theoretical enrichment of organizational identity research. All studies under this *aggregated dimension* share a general thematic focus on either organizational identification or how organizational identity is conceptualized. The last aggregated dimension *methodology/research approach* (topics: 2, 4, 5) summarizes papers that highlight or use different empirical methods, including quantitative, explorative/qualitative, and mixed-method approaches (e.g., Ordinary Least Squares, semi-structured interviews, or bibliographic analysis).

**Fig. 2:**
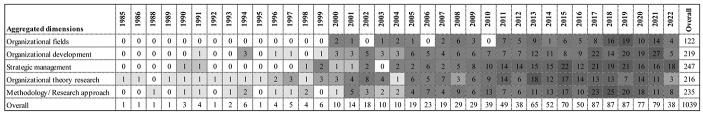
Dominant Appearances of Aggregated Dimensions per Year

The seminal paper of Albert and Whetten (1985) is primarily represented in Topic 17, which is assigned to the aggregated dimension o*rganizational theory research*. This is also the dimension that has a constant or increasing publication trend since the publication of Albert and Whetten’s seminal paper until 2015 (Fig. 2). Furthermore, the paper is also assigned to Topic 10 (*organizational development*) and Topic 15 (*strategic management*). In contrast to the declining number of publications in the aggregated dimension *organizational fields* (peaked between 2011 and 2015) as well as in *organizational development* (peaked between 2015 and 2021), an increasing number of publications in the aggregated dimension of *strategic management* can be observed (since 2015). Hence, organizational identity literature based on Albert and Whetten (1985) demonstrates an increasing interest in the strategic use of organizational identity.

In addition to these topics, which we were able to assign to the five *aggregated dimensions*, there are four other topics, which, however, are only mentioned as the most dominant in a total of two papers. Consequently, these topics appear to be insignificant and are not further considered in the analysis.

#### Investigating research fields

An investigation of the dissemination of Albert and Whetten’s (1985) concept in various research fields and a comparison of the same retraces the evolution that leads to the popularity of the seminal paper. Out of 1,041 papers in the dataset, more than half (589 papers) can be assigned to the business field,^6^ and 44% (452 papers) outside the field (e.g., communication, computer science, economics, education, environment, finance, health, psychology, and sociology). Most studies outside the field have been published in the last 12 years. Notably, the growing interest in Albert and Whetten’s (1985) work in business research has induced an increasing attention to surrounding fields (Appendix 3). In 1988, three years after the publication of Albert and Whetten’s (1985) paper, an interdisciplinary paper (main field: political science) was published. Nevertheless, an increase in publications across research fields was observed 2013 onwards. Albert and Whetten’s (1985) organizational identity concept has gained significant popularity, especially in sociology, psychology, and education, with 85 of 95 publications having been published since 2009.

### Dissemination of the basic criteria, groups of hypotheses and methodological approach

A consideration of the keyword-in-context analysis by searching citations of Albert and Whetten’s (1985) work in the dataset allows for the investigation of how and which criteria of their basic definition (central, distinct, enduring), groups of hypotheses, and their suggestion of a metaphor analysis as a methodological approach are embedded in the literature. Table 3 demonstrates the final coding scheme of the keyword-in-context analysis via the three criteria, four hypotheses groups, and the methodological approach considered by studies that cite them, with their absolute and relative appearances in the data sample. A comparison of the relative frequencies of the three criteria (central character, distinctiveness, and enduring) that define organizational identity and the groups of hypotheses evidence that the criteria are important aspects among all *aggregated dimensions*, as these are mentioned in more than half of the papers. Among the groups of hypotheses, the internal perception of organizational identity is distinctive, as slightly more than half of all papers (52.99%) refer to it. In particular, the aggregated dimension *organizational development* frequently refers to the topic of hypotheses. Most (90.95%) of the papers assigned to this aggregated dimension refer to the internal perception of organizational identity. In particular, the papers refer to struggles, challenges and discontinuity in organizations and how internal sense-making and coping strategies are used to overcome them (e.g., Hahn et al. 2014; Kump 2019; Ravasi et al. 2011; Wenzel et al. 2020); the papers also reference Albert and Whetten (1985) as a concept that emphasizes the member perspective. Moreover, the group of hypotheses that concern the mono and dual identities is also considered in research, but less frequently. Thereby, it is most frequently used in the aggregated dimensions *organizational development* (20.60%) and *organizational theory research* (23.00%). The majority of the papers categorized into these aggregate dimensions investigate the construction and framing of identities in organizations (Corley 2004; Fraser and Ansari 2021; Heckert et al. 2020) and cite Albert and Whetten (1985) with reference to their concept of holographic and ideographic organizations to differentiate between these forms of mono and dual identities. Further, less than one-fifth of the papers refer to the two remaining groups of hypotheses (organization’s life-cycle and comparison of organizations) and the metaphor analysis as methodological approach. Thus, these groups of hypotheses appear to have less impact on research than the basic criteria and the other groups of hypotheses introduced by Albert and Whetten (1985). Although the frequency of occurrence for these two groups of hypotheses is generally low, our coding results of the keyword-in-context analysis illustrate that the aggregated dimension *organizational development* (14.57%) is most likely to appear in the hypothesis group organization’s life-cycle, and the aggregated dimension *organizational field* (10.43%) is most likely to appear in the hypothesis group comparison of organization types. This appears to be appropriate because the former aggregated dimension considers papers that focus on how organizations change and develop over the course of their existence. The latter aggregated dimension investigates different organizational fields, whereby it is also considered whether organizations can be either utilitarian or normative in nature.

An examination of how Albert and Whetten (1985) are referenced demonstrates that most citations (77%) in our keyword-in-context search refer to them indirectly by listing them among other studies in the field to support an argument. Following a further examination, six of 953 (< 1%) papers included in the keyword-in-context analysis cite one of Albert and Whetten’s (1985) hypotheses directly. More specifically, a direct reference is made to three of the four groups of hypotheses, with no direct reference made to the hypotheses categorized to the group *comparison of organization types*. Furthermore, based on our KWIC, we found that 35 papers cite Albert and Whetten’s (1985) seminal paper to note differences in contrast to the seminal work or specific parts therein and to distinguish themselves from it.

**Fig. 3:**
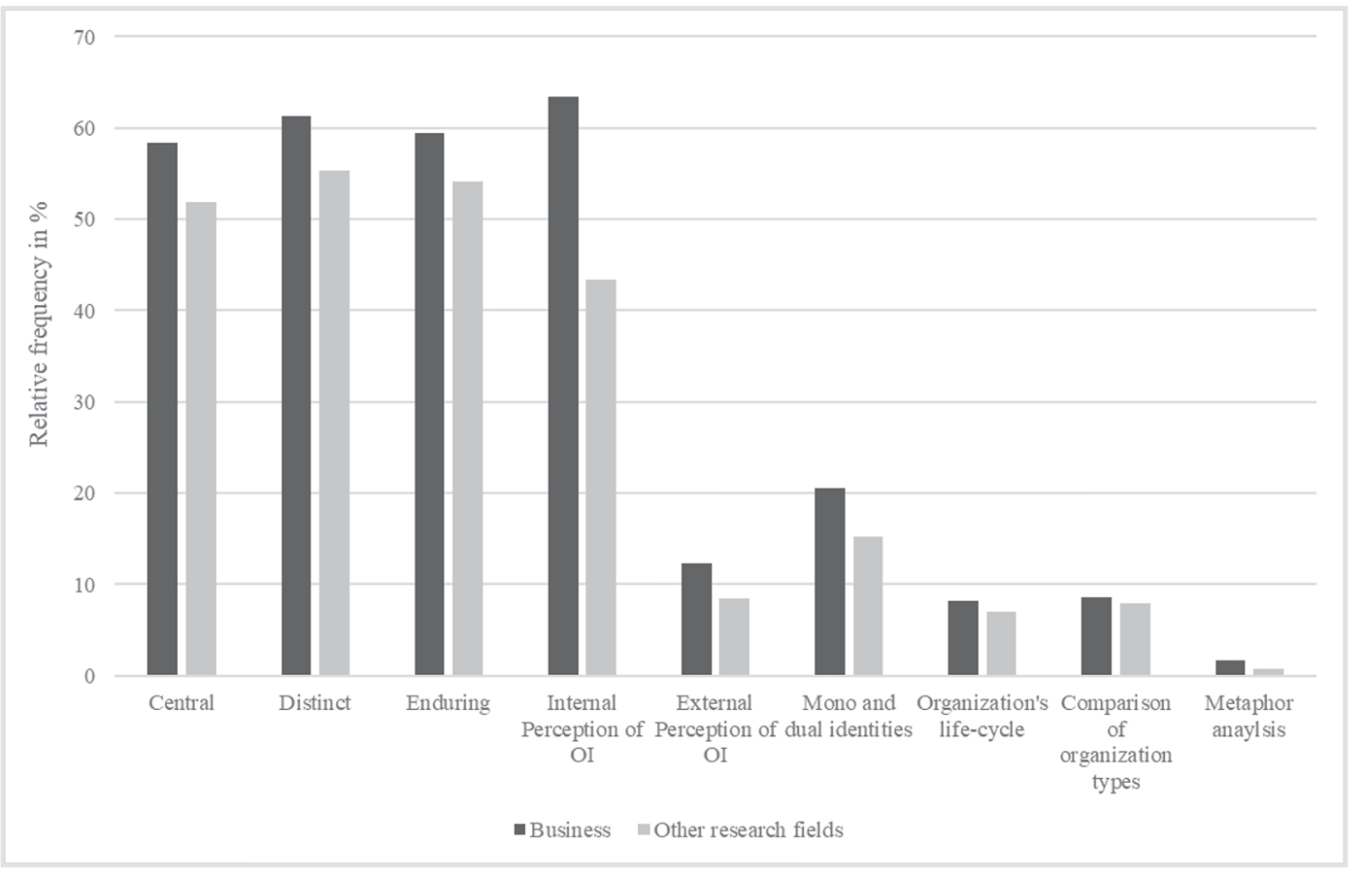
Distribution of Basic Criteria, Groups of Hypotheses and Methodological Approach In- and Outside of Business Research

With regard to Albert and Whetten’s (1985) dissemination of their three criteria of the basic definition, groups of hypotheses, or metaphor analysis in business and in other research fields, our results demonstrate that they are slightly more referenced in the business research (Fig. 3). Further, there are no major contextual differences between citations within and outside business research with one exception. A more significant difference can be observed in the group of hypothesis that consider the internal perception of organizations; 63% of the papers from the business field refer to this, whereas only 43% from other research fields do so.

**Table 3:** Absolute Frequency of **C**oding Results of the Keyword-In-Context Search per Aggregated Dimension

		All documents (relative)	Organizational field (relative)	Organizational development (relative)	Strategic management (relative)	Organizational theory research (relative)	Methodology/ Research approach (relative)
**Basic criteria of OI**	**Central**	513 (53.83%)	56 (48.70%)	123 (61.81%)	119 (51.51%)	108 (54.00%)	107 (51.69%)
	**Distinct**	542 (56.87%)	57 (49.57%)	125 (62.81%)	131 (56.71%)	122 (61.00%)	107 (51.69%)
	**Enduring**	527 (55.30%)	61 (53.04%)	123 (61.81%)	127 (54.98%)	110 (55.00%)	106 (51.21%)
**Groups of hypotheses**	**Internal perception of OI**	505 (52.99%)	57 (49.57%)	181 (90.95%)	104 (45.02%)	85 (42.50%)	78 (37.68%)
	**External perception of OI**	98 (10.28%)	13 (11.30%)	33 (16.58%)	21 (9.09%)	11 (5.50%)	20 (9.66%)
	**Mono & dual identities**	168 (17.63%)	19 (16.52%)	41 (20.60%)	38 (16.45%)	46 (23.00%)	24 (11.59%)
	**Organization’s life cycle**	71 (7.45%)	7 (6.08%)	29 (14.57%)	8 (3.46%)	15 (7.50%)	12 (5.80%)
	**Comparison of organization types**	77 (8.08%)	12 (10.43%)	18 (9.05%)	19 (8.23%)	15 (7.50%)	13 (6.28%)
**Methodological approach**	**Metaphor analysis**	12 (1.26%)	3 (2.61%)	2 (1.01%)	0 (0%)	6 (3.00%)	1 (0.40%)
*Note*. The table presents the absolute (and relative frequency in brackets) of keyword appearances of our keyword-in-context analysis. Due to formatting styles (footnotes, tables, etc.), not all 1,040 papers that reference Albert and Whetten (1985) could be included, which resulted in a *n* for the analysis of 953.							

#### Journal publication over the years

**Fig. 4:**
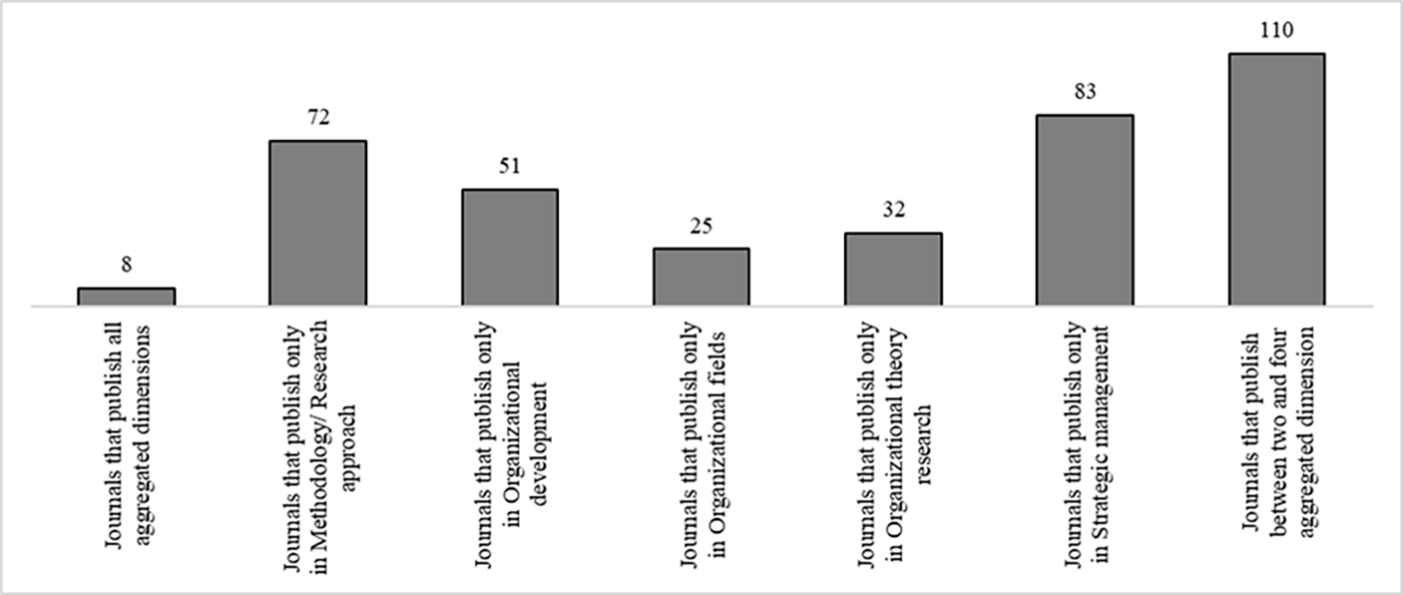
Journal Publication Patterns of Aggregated Dimension (absolute numbers)

An examination of the years in which studies that referenced Albert and Whetten (1985) were published illustrates that some (6%) were published in the 1985–2001 period. To evaluate the journals in which these studies were published and examine their significance to the scientific community, we used the SCImago Journal Rank (SJR) indicator, which considers “not only the prestige of the citing scientific journal but also its closeness to the cited journal using the cosine of the angle between the vectors of the two journals’ cocitation profiles” (Guerrero-Bote and Moya-Anegón 2012, p. 674). Thus, we observe that high-ranked journals,^7^ such as the *Strategic Management Journal* (9.4), *Academy of Management Journal* (10.9), and *Administrative Science Quarterly* (17.4), recognized the importance of organizational identity before 2000, and thus, before many other journals (Appendix 4). Lower-ranked journals that belong to respective scientific publication outlets in the Web of Science Core Databank generally published studies on Albert and Whetten’s (1985) organizational identity concept only after 2000. A closer investigation of the assignment of journal publications and aggregated dimensions demonstrates a strong prevalence of *strategic management*, with more than 25% of all journals publishing articles under this aggregated dimension (Fig. 4). Thus, the publication of papers that thematically focus on identity and strategy is particularly popular, especially in recent years (Fig. 2).

Further, it is less common for journals to publish articles that investigate only the aggregated dimension *organizational fields*. Nearly all high-ranked journals publish articles with at least three out of the five aggregated dimensions (Appendix 4).

**Fig. 5:**
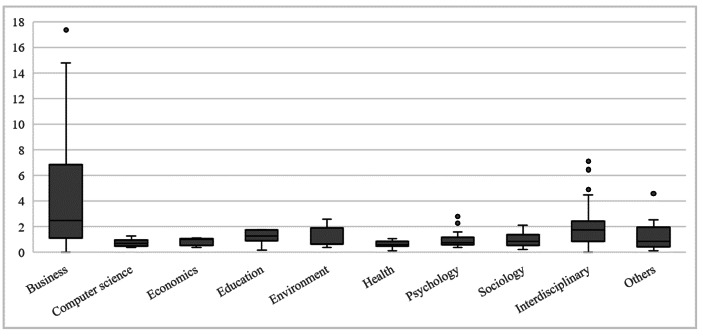
SCImago Journal Ranking per Research Field

With regard to the various fields of research that reference Albert and Whetten (1985), studies published in the business field are published in high-ranking journals (Fig. 5), which indicates organizational identity’s high relevance and interest among business researchers. Figure 5 demonstrates the other seven research fields and their journal rankings. Two additional boxplots incorporate interdisciplinary work and unspecified research fields (labeled as “Others” in Fig. 5). Relevant studies published in other research fields were often published in specific journals as one-time publications. While the highest SCImago business journal rank score was 17.36, the closest highest score of journals outside the business field was 7.12.

#### Network analysis

**Fig. 6:**
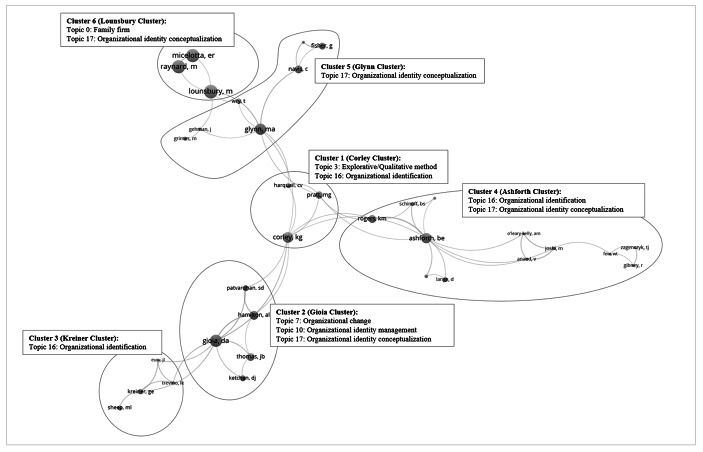
Sub-Author-Network in Organizational Identity Research

A contextualization of the inductive content analysis of the occurring topics with author network data reveal that from the 2,134 authors, 264 meet the threshold of having published at least 2 documents with other authors (the network plot is automatically reduced to 33 authors). Thus, we investigated subnetworks that resulted in six clusters that could be categorized for a more detailed overview of author networks (Fig. 6). Notably, one author dominates the individual clusters. We call them “central authors” to better differentiate the clusters^8^. Central authors often connect various subnetworks. Thus, five subnetworks are related via publications with two other subnetworks. Only the Corley Cluster is related to all other five subnetworks, which exhibits high diversity. The subnetwork with the most publications is the Glynn Cluster (22), followed by the Corley Cluster (18), the Ashforth Cluster (18), and the Gioia Cluster (18). The Kreiner (7) and Lounsbury (4) Clusters appear to have been less pronounced until recently. They were established in 2006 and later, whereas the others were established in the late ‘80s or early ‘90s. Following the contextualization of the subnetworks with the text mining results, it can be observed that the aggregated dimension *organizational theory research* is the only aggregated dimension that is referred to by all clusters. Furthermore, it is also the dominant dimension in all the clusters, with the exception of the Gioia Cluster, wherein the aggregated dimension *organizational development* occurs more frequently. In the Lounsbury Cluster the *organizational theory research* occurs as frequently as the aggregated dimension of the *organizational field*. Moreover, our network analysis demonstrates that Topic 17 is by far the most recurrent topic. Thus, central authors who reference Albert and Whetten (1985) concentrate on fundamental organizational identity research and how it can be characterized.

Further, the author network and dominant topic visualization highlight that next to Topic 17, authors frequently refer to Topic 16, which focuses on *organizational identification* and is also categorized to the *organizational theory research* aggregated dimension. Topic 16 is most frequently used by the Ashforth Cluster. Thus, within the Ashforth and Glynn Clusters, authors especially focus on the basic constructs of organizational identity in their research. Thereby, the Glynn Cluster is more focused on the *organizational identity conceptualization* and the Ashforth Cluster on the *organizational identification*.

What is remarkable is that only half of the clusters focuses on the aggregated dimension *strategic management*, which has a low number of references overall, although it is the most dominant and thus also the most frequently mentioned aggregated dimension in 247 papers (Fig. 2). However, this is possibly due to the fact that the topics of the aggregated dimension *strategic management* have received more attention only in recent years and that no fixed groups of authors have yet been established here.

With regard to the research fields, the network analysis crystallizes six subnetworks of authors with a high density of publications in organizational identity research from various research fields. Each subnetwork includes authors from the business research field. Moreover, all, except the Lounsbury Cluster include publications from research fields outside the business literature.

## Directions for future research

With regard to the keyword-in-context analysis, most studies that reference Albert and Whetten (1985) cite them for the three basic criteria that characterize organizational identity, while the 32 hypotheses introduced in the seminal work are usually ignored. Hence, most organizational identity studies overlook Albert and Whetten’s (1985) proposition of the evolving character during the life-cycle, and the comparison of organization types, and fail to mention the metaphor analysis as a methodological approach. Although the results of our keyword-in-context analysis reveal that the hypotheses group *comparison of organizations types* has not been frequently used so far, it can be stated that it has already been considered in research (e.g., Moss et al. 2011; Scherer 2017). However, researchers commonly do not use the terms “utilitarian and normative” by Albert and Whetten (1985), which results in citations of their seminal paper being missed. Accordingly, three key research gaps emerge from the ignored organizational identity subjects and text mining results (Appendix 5). The following discussion presents the research gaps by embedding them in the current research context.

### The evolving character of Organizational Identity (life-cycle of organizations)

The results indicate that little reference is made to Albert and Whetten’s (1985) life-cycle process. Their seminal paper notes that this identity framework “examine[s] how new roles come into existence, how organizations choose (or back into) one role rather than another, and how that action affects the organization’s internal and external identity” (Albert and Whetten 1985, p. 273). Thus, they formulate various time-dependent hypotheses with regard to organizational identity, some of which have been considered in the literature (e.g., Hypothesis 11, where a takeover or acquisition impacts the organization’s mission, values, and identity). Clark et al. (2010) investigate this concern and note that an organization requires a “transitional identity” in a merger or acquisition. Further, studies have considered Albert and Whetten’s (1985) Hypothesis 8 (the loss of an identity sustaining element). For instance, Basque and Langley (2018) reveal different methods by which organizations invoke founders long after they have passed away. Boers and Ljungkvist (2019) also illustrate that a founder’s legacy remains despite new owners, which results in the maintenance of the “former” organizational identity. Organizations continue to adhere to central figures because their loss could induce a diminished sense of collective identity among diverse stakeholders (Hyde and Thomas 2003). However, Albert and Whetten (1985) note that a new element as a suitable successor is required after the loss of an identity sustaining element. Extant research has focused only on how the spirit of identity sustaining elements has survived, and not on what organizations do when the maintenance or the persistence of identity sustaining organizational elements does not work, and how suitable alternatives or successors (e.g., new personalities, artifacts or rituals that can be characteristics of organizational identity (Watkiss and Glynn 2016)) are found in such situations. Thus, we suggest that future research should analyze cases where new suitable identity creating successor elements are sought and implemented in the organizational identity, and investigate the influence of such implementation. Additionally, it is crucial to investigate how equivalent identity-creating succession elements are selected. This would improve the understanding of change processes, such as mergers and crises, and aid the successful, strategic implementation of successors.

Hypothesis 10, wherein organizations consider issues of their organizational identity when their profits or other resources exceed the normal use or growth, has also received little consideration. Hence, Dörrenbächer et al. (2017, p. 7) note that research on the link between organizational identity and firm growth remains in its infancy. This gap exists because organizational growth and identity cannot be determined simultaneously (Davidsson and Wiklund 2017). More precisely, organizational identity can be determined relatively easily at a given point in time (e.g., at the time of founding). However, an organization can change so much over time that it is no longer the organization it used to be, and the definition of its organizational identity determined at one point in time is no longer the same (Davidsson and Wiklund 2017). Thus, an investigation of organizational identity and its change during rapid growth in profit or other resources is a promising avenue for future research. Organizational identity during slow growth (Hypothesis 12), which has also received scant attention, can also be focused on.

### Metaphor analysis as methodological approach to organizational identity

The keyword-in-context analysis indicates that studies referencing Albert and Whetten (1985) infrequently consider and mention the metaphor analysis methodological approach (Table 3). In total, 17.63% of studies reference mono or dual identities, but only 1.26% consider metaphor analysis, even though this is recommended by Albert and Whetten (1985) to analyze dual and hybrid identities.

Further, studies on organizational identity and metaphor analysis employ a theoretical perspective rather than an empirical investigation. For example, Cornelissen (2002) offers a method of metaphor analysis to construct an organizational identity theory. Moreover, different metaphors are employed to deconstruct and analyze common meta-theoretical perspectives in the field. Thus, a framing metaphor can be found in the social constructionist perspective; a categorization metaphor is used within the social identity and ecology scholarship; and the personification metaphor is part of the social actor perspective (Cornelissen et al. 2016). Ashforth et al. (2020) discuss the personification metaphor as anthropomorphism. Further, studies offer different propositions on using anthropomorphism to construct an organizational identity. Haslam et al. (2003) argue that organizational identity cannot simply be a metaphor but also a specific psychological construct that influences organizational behavior. Oswick et al. (2002) highlight that the investigation of metaphors contributes to organizational analysis. Rather than an examination of how organizations use metaphors to construct their organizational identity, Oswick and Oswick (2020), more recently, highlighted elements for which metaphors have been used with respect to the organizational identity concept over time. König et al. (2018) conducted a study that examined how CEOs’ use of metaphorical communication affects perceptions of their organization. Their results reveal that the effect of metaphorical communication depends on the recipients of the message. Thereby, the more CEOs use metaphorical communication, the greater is the instance of positive (negative) reports about the organization by journalists (analysts). Although König et al. (2018) have addressed the empirical evidence of metaphors and made preliminary findings on their use, the authors also state that it would be interesting to analyze the shareholder and stakeholder groups who also contribute to the construction of organizational identity. Based on this study, it can be argued that metaphor analysis^9^ should continue to be used to examine different significant effects, especially in light of the fact that few studies concentrate on how metaphors are strategically used in organizations to construct an organizational identity. Therefore, future research should focus less on the theoretical aspect of using metaphors in identity construction, and rather conduct extended metaphor analysis, to pay more attention to the empirical evidence of metaphors used by organizations as actors and organizational members.

### Demand for a better operationalization

The systematic analysis demonstrates that studies focusing on the *organizational theory* aggregated dimension often investigate organizational identity claims and their construction qualitatively. For example, studies analyze organizational web pages (Oertel and Thommes 2018; Sillince and Brown 2009), interviews (Alvesson and Empson 2008; Aracı 2019), archival data (Anteby and Molnár 2012; Schultz and Hernes 2013), or a qualitative combination and triangulation of the data (Ravasi and Schultz 2006; Törmälä and Gyrd-Jones 2017).^10^

Further, studies often focus on specific industries by the investigation of how and by which claims organizational identity is constructed; examples include the wine (Giuliani et al. 2015; Zamparini and Lurati 2013), beer brewing (Kroezen and Heugens 2012), watchmaking (Oertel and Thommes 2018; Raffaelli 2019), or hospitality (Martínez et al. 2014) industries. Evidently, there are only a few exceptions. First, Akerlof and Kranton (2005) employ an economic approach to demonstrate that employees whose identities match their organizations accept lower pay than those without a matching identity. They also note that the alignment of the identity and goals of employees with their organizations can increase profits. Thus, future research can quantitatively examine the effect of organizational identity on other individuals and organizational aspects. Second, Glynn and Abzug (2002) employ regression analysis to show how names of organizations changed, and argue that they are an important aspect of organizational identity. They illustrate, for example, that shorter organizational names are more comprehensible. However, their quantitative approach is not sufficient to operationalize organizational identity, as the authors consider only one part of organizational identity by reference to the organization’s name. Moreover, Dukerich et al. (2002, p. 515) note that “there is no established measure of organizational identity that can be used in survey research.” Thus, they use the attributes of the organizations that have been mentioned in their sample. In contrast, other researchers (e.g., Milliken 1990) quantitatively investigate organizational identity strength, which is distinguishable from the construct of organizational identity (Cole and Bruch 2006). Thus, further research on the organizational identity concept and its construction through the use of quantitative data can enable the obtainment of new approaches for strategic management. Hence, the claims found and investigated could be combined and validated to create items. Therefore, new (economic) insights and links between organizational identity and other important organizational elements can be found and investigated further. Furthermore, the items that define organizational identity can be used to analyze and compare organizational identity in large-scale inter and intra organizational surveys, or even in different industries. Due to a higher degree of standardization, these surveys offer a higher degree of comparability, which not just opens new research questions but also provides a framework for testing qualitative results on a larger sample.

## Conclusion

Two limitations were encountered in the analysis of our dataset, which we would like to discuss in the interest of transparency. Despite the use of the assigned research fields of the Web of Science databank, it was observed that these cannot always be clearly differentiated.^11^ Thus, the use of other research field differentiations may lead to different findings with regard to RQ1b. Second, rather than making a systematic literature review of the entire field of organizational identity research, we analyzed how the work of Albert and Whetten (1985) has been disseminated. Hence, our findings and the research gaps observed cannot be generalized without further investigation.

Thus, this study provides an overview of how parts of Albert and Whetten’s (1985) organizational identity conceptualization have been incorporated into subsequent research and how they have influenced research. In this manner, the paper highlights research on organizational identity per the basic criteria, groups of hypotheses, and methodological approach introduced by Albert and Whetten (1985) that require further investigation. Accordingly, even though the basic criteria (central character, distinctiveness, and temporal continuity) of organizational identity have gained popularity in the last two decades, other aspects, especially the many hypotheses in the seminal work, have not been considered over the past 37 years, or have been mentioned without explicit reference to Albert and Whetten (1985). Therefore, this study highlights research areas in organizational identity that offer scope for future studies.

## Electronic supplementary material

Below is the link to the electronic supplementary material.

Supplementary Material 1

## Data Availability

The datasets generated and analyzed during the current study are not publicly available due to legal access restrictions of the papers but are available from the corresponding author on reasonable request.

## Footnotes

1 Based on a Google Scholar search for “organization* ident*”.

2 We refer to 32 hypotheses even though the last-mentioned hypothesis is named H33 because of a missing hypothesis (i.e., H21).

3 Note that this expression is standard. The pipe symbol is used to match “and” and “&” in our dataset.

4 We used the assigned research fields to each text per the Web of Science databank.

5 Although the *aggregated dimensions* include different numbers of topics, we can exclude a bias because the number of assigned papers to the *aggregated dimensions* is almost equally distributed (see Fig. 2 for the manually coded *aggregated dimensions* by their appearance as the main topic and year).

6 Using the categorization system of Web of Science.

7 SCImago, (n.d.). SJR—SCImago Journal & Country Rank [Portal]. Retrieved from http://www.scimagojr.com.

8 Cluster 1 relates to publications around Corley, K. and is therefore the Corley Cluster. Cluster 2 relates to publications around Gioia, D. and is therefore the Gioia Cluster. Cluster 3 relates to publications around Kreiner, G. and is therefore the Kreiner Cluster. Cluster 4 relates to publications around Ashforth, B. and is therefore the Ashforth Cluster. Cluster 5 relates to publications around Glynn, M. and is therefore the Glynn Cluster. Cluster 6 relates to publications around Lounsbury, M. and is therefore the Lounsbury Cluster.

9 Compared to the past, there are other data analysis methods used today that have developed due to faster data collection or Big Data, such as sentiment analysis, which can also be used for analysis in the organizational identity research field. However, it can be stated that the use of the metaphor analysis proposed by Albert and Whetten (1985) can still yield new and significant results.

10 Our results reveal an aggregated dimension *methodology/research approach* representing the different research designs implemented in the papers. However, the used research designs represented in the papers are not only used for investigating organizational identity but may also refer to other theoretical concepts since we use all papers referring to Albert and Whetten (1985). Nevertheless, in this case, there are also more qualitative papers than quantitative papers.

11 For example, *health care science & services* and *health policy & service*; *psychology applied*, *psychology clinical*, and *psychology educational;* or *public administration*, *business*, and *management*.
