# Supplementary material for: Since Albert and Whetten: the dissemination of Albert and Whetten’s conceptualization of organizational identity
Source: Manag Rev Q. 2022 Dec 2:1–29. Online ahead of print. doi: 10.1007/s11301-022-00311-7 (PMC9716518; doi:10.1007/s11301-022-00311-7)
Supplement: Supplementary file 1 — Supplementary Material 1 [file 11301_2022_311_MOESM1_ESM.docx]

**Appendix 1** Full list of papers included in dataset

Albert, S.; Whetten, D. (1985). Organizational identity. *Research in Organizational Behavior*, 7, 263-295.

Abemethy, M.; Jiang, L.; Kuang, Y. (2019). Can organizational identification mitigate the CEO horizon problem? *Accounting Organizations and Society*, 78, 101056.

Abolafia, M.; Hatmaker, D. (2013). Fine-tuning the signal: image and identity at the federal reserve. *International Public Management Journal*, 16(4), 532-556.

Abrahamsson, G.; Englund, H.; Gerdin, J. (2011). Organizational identity and management accounting change. *Accounting Auditing & Accountability Journal*, 24(3), 345-376.

Abratt, R.; Kleyn, N. (2012). Corporate identity, corporate branding and corporate reputations Reconciliation and integration. *European Journal of Marketing*, 46(7-8), 1048-1063.

Acharya, A..; Gras, D.; Krause, R. (2022). Socially Oriented Shareholder Activism Targets: Explaining Activists' Corporate Target Selection Using Corporate Opportunity Structures. *Journal of Business Ethics,* 178(2), 307-323.

Achrol, R. (1997). Changes in the theory of interorganizational relations in marketing: Toward a network paradigm. *Journal of the Academy of Marketing Science*, 25(1), 56-71.

Adla, L.; Eyquem-Renault, M.; Gallego-Roquelaure, V. (2020). From the Leader's Values to Organizational Values: Toward a Dynamic and Experimental View on Value Work in SMEs. *Management,* 23(1), 81-101.

Afshari, L.; Young, S.; Gibson, P.; Karimi, L. (2020). Organizational commitment: exploring the role of identity. *Personnel Review*, 49(3), 774-790.

Aglargoz, O. (2017). We are at this campus, there is nothing in this campus ... ': Socio-spatial analysis of a university campus. *Tertiary Education and Management*, 23(1), 1-15.

Aguilera, R.; Judge, W.; Terjesen, S. (2018). Corporate governance deviance. *Academy of Management Review*, 43(1), 87-109.

Ahearne, M.; Bhattacharya, C.; Gruen, T. (2005). Antecedents and consequences of customer-company identification: Expanding the role of relationship marketing. *Journal of Applied Psychology*, 90(3), 574-585.

Albert, S. (1990). Mindfulness, an important concept for organizations - a book review essay on the work of Ellen Langer. *Academy of Management Review*, 15(1), 154-159.

Alfoldi, E.; McGaughey, S.; Clegg, L. (2017). Firm Bosses or Helpful Neighbours? The Ambiguity and Co-Construction of MNE Regional Management Mandates. *Journal of Management Studies*, 54(8), 1170-1205.

Allen, M.; Ericksen, J.; Collins, C. (2013). Human Resource Management, Employee Exchange Relationships, and Performance in Small Businesses. *Human Resource Management*, 52(2), 153-173.

Almeida do Monte, A. L.; Pompeu, R. M.; Holanda, M. M. (2022). Corporate museum as a strategic cultural asset to strengthen the brazil brand. *Revista Brasileira de Marketing,* 21(1), 185-215.

Altman, Y.; Baruch, Y. (2010). The organizational lunch. *Culture and Organization,* 16(2), 127-143.

Alvesson, M. (1994). Talking in organizations - managing identity and impressions in an advertising agency. *Organization Studies,* 15(4), 535-563.

Alvesson, M.; Robertson, M. (2006). The best and the brightest: The construction, significance and effects of elite identities in consulting firms. *Organization,* 13(2), 195-224.

Alvesson, M.; Karreman, D. (2007). Unraveling HRM: Identity, ceremony, and control in a management consulting firm. *Organization Science*, 18(4), 711-723.

Amdam, R.; Lunnan, R.; Bjamar, O.; Halse, L. (2020). Keeping up with the neighbors: The role of cluster identity in internationalization. *Journal of World Business,* 55(5), 101125.

Amemic, J.; Craig, R. (2022). Evaluating assertions by a Wells Fargo CEO of a 'return to ethical conduct'. *Leadership.* 18(3), 400-426.

Amis, J. (2018). Understanding Organization Change and Innovation: A Conversation with Mike Tushman. *Journal of Change Management*, 18(1), 23-34.

Anand, V.; Joshi, M.; O'Leary-Kelly, A. (2013). An Organizational Identity Approach to Strategic Groups. *Organization Science*, 24(2), 571-590.

Anderson, J.; Hakansson, H.; Johanson, J. (1994). Dyadic business relationships within a business network context. *Journal of Marketing*, 58(4), 1-15.

Anderson, L.; Guo, J. (2020). Paradoxical Timelines in Wells Fargo's Crisis Discourse: Expanding the Discourse of Renewal Theory. *International Journal of Business Communication,* 57(2), 212-226.

Anisimova, T.; Mavondo, F. (2014). Aligning Company and Dealer Perspectives in Corporate Branding: Implications for Dealer Satisfaction and Commitment. *Journal of Business-To-Business Marketing*, 21(1), 35-56.

Anisimova, T.; Mavondo, F. (2010). The performance implications of company-salesperson corporate brand misalignment. *European Journal of Marketing*, 44(6), 771-795.

Ann Glynn, M.; Watkiss, L. (2020). Of Organizing and Sensemaking: From Action to Meaning and Back Again in a Half-Century of Weick's Theorizing. *Journal of Management Studies.* 57(7), 1331-1354.

Annosi, M.; Foss, N.; Brunetta, F.; Magnusson, M. (2017). The Interaction of Control Systems and Stakeholder Networks in Shaping the Identities of Self-Managed Teams. *Organization Studies,* 38(5), 619-645.

Anohina-Naumeca, A.; Tauginiene, L.; Odineca, T. (2018). Academic integrity policies of Baltic state-financed universities in online public spaces. *International Journal for Educational Integrity*, 14(1), 1-14

Anteby, M. (2008). Identity incentives as an engaging form of control: Revisiting leniencies in an aeronautic plant. *Organization Science*, 19(2), 202-220.

Anteby, M.; Molnar, V. (2012). Collective memory meets organizational identity: remembering to forget in a firm's rhetorical history. *Academy of Management Journal*, 55(3), 515-540.

Appleby, K.; Bullinger, B.; Schneider, A. (2018). STEM selves: Women's identity projects and their assessment of future employers in technical fields. *Scandinavian Journal of Management,* 34(4), 311-325.

Araci, O. (2019). Using content analysis to reveal organizational identity orientation Evidence from the newspaper industry. *Management Research Review,* 42(3), 314-331.

Araci, O.; Boal, K.; Gurbuz, G. (2020). An approach to gain more with less concessions: Transitive strategic responses of organizations under the effect of organizational identity and relations with environment. *Journal of East European Management Studies,* 25(2), 384-407.

Asencio, H.; Sun, R. (2020). The Effects of Leadership on Employee Trust: A Longitudinal Study of United States Federal Agencies. *International Journal of Public Administration,* 43(14), 1237-1251.

Ashforth, B.; Humphrey, R. (1997). The ubiquity and potency of labeling in organizations. *Organization Science*, 8(1), 43-58.

Ashforth, B.; Mael, F. (1989). Social identity theory and the organization. *Academy of Management Review*, 14(1), 20-39.

Ashforth, B.; Mael, F. (1996). Organizational identity and strategy as a context for the individual. *Advances in Strategic Management*, 13, 19-64.

Ashforth, B.; Vaidyanath, D. (2002). Work organizations as secular religions*. Journal of Management Inquiry*, 11(4), 359-370.

Ashforth, B.; Joshi, M.; Anand, V.; O'Leary-Kelly, A. (2013). Extending the expanded model of organizational identification to occupations. *Journal of Applied Social Psychology,* 43(12), 2426-2448.

Ashforth, B.; Reingen, P. (2014). Functions of Dysfunction: Managing the Dynamics of an Organizational Duality in a Natural Food Cooperative*. Administrative Science Quarterly,* 59(3), 474-516.

Ashforth, B.; Rogers, K.; Corley, K. (2011). Identity in Organizations: Exploring Cross-Level Dynamics. *Organization Science*, 22(5), 1144-1156.

Ashforth, B.; Rogers, K.; Pratt, M.; Pradies, C. (2014). Ambivalence in Organizations: A Multilevel Approach. *Organization Science*, 25(5), 1453-1478.

Ashforth, B.; Schinoff, B.; Brickson, S. (2020). My company is friendly, mine’s a rebel: anthropomorphism and shifting organizational identity from what to who. *Academy of Management Review,* 45(1), 29-57.

Ashforth, B..; Schinoff, B.; Rogers, K. (2016). I identify with her, i identify with him: unpacking the dynamics of personal identification in organizations. *Academy of Management Review*, 41(1), 28-60.

Ashmos Plowman, D.; Baker, L.; Beck, T.; Kulkarni, M.; Thomas Solansky, S.; Villarreal Travis, D. (2007). Radical change accidentally: The emergence and amplification of small change. *Academy of Management Journal*, 50(3), 515-543.

Aspara, J.; Tikkanen, H. (2011). Corporate marketing in the stock market The impact of company identification on individuals' investment behaviour. *European Journal of Marketing*, 45(9-10), 1446-1469.

Astrachan, C.; Botero, I. (2018). We are a family firm: An exploration of the motives for communicating the family business brand. *Journal of Family Business Management*, 8(1), 2-21.

Atakan-Duman, S.; Pasamehmetoglu, A.; Bozaykut-Buk, T. (2019). The Challenge of Constructing a Unique Online Identity Through an Isomorphic Social Media Presence. *International Journal of Communication,* 13, 160-180.

Atienza, C. (2017). Building Organizational Identity: an Insider Action Research from a Founder's Viewpoint. *Systemic Practice and Action Research,* 30(6), 569-592.

Avidar, R. (2017). Public relations and social businesses: The importance of enhancing engagement. *Public Relations Review*, 43(5), 955-962.

Avila, L.; Amorim, M. (2021). Organisational Identity of Social Enterprises: A Taxonomic Approach. *Voluntas,* 32(1), 13-27.

Baba, S.; Heimissi, O.; Hafsi, T. (2021). National Identity and Organizational Identity in Algeria: Interactions and Influences. *Management,* 24(2), 66-85.

Backer, L. (2008). Narrating organisational identities by way of evolutionary tales - Talking Shell from an oil to an energy company. *Scandinavian Journal of Management,* 24(1), 33-43.

Balmer, J. (2009). Corporate marketing: apocalypse, advent and epiphany. *Management Decision,* 47(4), 544-572.

Balmer, J. (2017). The corporate identity, total corporate communications, stakeholders' attributed identities, identifications and behaviours continuum. *European Journal of Marketing*, 51(9-10), 1472-1502.

Balmer, J.; Burghausen, M. (2015). Introducing organisational heritage: Linking corporate heritage, organisational identity and organisational memory. *Journal of Brand Management*, 22(5), 385-411.

Balmer, J.; Chen, W. (2015). Corporate heritage brands in China. Consumer engagement with China's most celebrated corporate heritage brand - Tong Ren Tang. *Journal of Brand Management*, 22(3), 194-210.

Balmer, J.; Chen, W. (2016). Corporate heritage tourism brand attractiveness and national identity. *Journal of Product and Brand Management*, 25(3), 223-238.

Balmer, J.; Stuart, H.; Greyser, S. (2009). Aligning Identity and Strategy: Corporate Branding at British Airways in the Late 20th Century. *California Management Review*, 51(3), 6-23.

Balser, D.; Carmin, J. (2009). Leadership Succession and the Emergence of an Organizational Identity Threat. *Nonprofit Management & Leadership,* 20(2), 185-201.

Ban, Z. (2017). Laboring Under the Cross: An Analysis of Discursive Tension and Identity in the Context of a Chinese House Church. *Management Communication Quarterly,* 31(2), 230-257.

Bankins, S.; Waterhouse, J. (2019). Organizational Identity, Image, and Reputation: Examining the Influence on Perceptions of Employer Attractiveness in Public Sector Organizations. *International Journal of Public Administration,* 42(3), 218-229.

Barge, J.; Lee, M.; Maddux, K.; Nabring, R.; Townsend, B. (2008). Managing dualities in planned change initiatives. *Journal of Applied Communication Research*, 36(4), 364-390.

Barnes, V.; Newton, L. (2018). Visualizing organizational identity: the history of a capitalist enterprise. *Management and Organizational History,* 13(1), 24-53.

Barnett, C.; Pratt, M. (2000). From threat-rigidity to flexibility - Toward a learning model of autogenic crisis in organizations. *Journal of Organizational Change Management*, 13(1), 74-88.

Barnett, M. (2008). An attention-based view of real options reasoning. *Academy of Management Review*, 33(3), 606-628.

Barnett, T.; Long, R.; Marler, L. (2012). Vision and Exchange in Intra-Family Succession: Effects on Procedural Justice Climate Among Nonfamily Managers. *Entrepreneurship: Theory and Practice,* 36(6), 1207-1225.

Barney, J. (2000). Context is crucial - Commonalities, differences, and subtle differences. *Advances in Strategic Management,* 17, 261-269.

Barraquier, A. (2013). A Group Identity Analysis of Organizations and Their Stakeholders: Porosity of Identity and Mobility of Attributes. *Journal of Business Ethics*, 115(1), 45-62.

Barros, M. (2010). Emancipatory Management: The Contradiction Between Practice and Discourse. *Journal of Management Inquiry*, 19(2), 166-184.

Bartel, C.; Wiesenfeld, B. (2013). The social negotiation of group prototype ambiguity in dynamic organizational contexts. *Academy of Management Review*, 38(4), 503-524.

Bartels, J.; Douwes, R.; de Jong, M.; Pruyn, A. (2006). Organizational identification during a merger: Determinants of employees' expected identification with the new organization. *British Journal of Management, 17*, S49-S67.

Bartels, J.; Peters, O.; de Jong, M.; Pruyn, A.; van der Molen, M. (2010). Horizontal and vertical communication as determinants of professional and organisational identification. *Personnel Review*, 39(1-2), 210-226.

Bartels, J.; Pruyn, A.; De Jong, M.; Joustra, I. (2007). Multiple organizational identification levels and the impact of perceived external prestige and communication climate. *Journal of Organizational Behavior,* 28(2), 173-190.

Barth-Farkas, F.; Vera, A. (2019). Leader Prototypicality and Displayed Power in the Police: An Empirical Analysis of the Impact on Leader Endorsement and Trust. *Policing,* 13(4), 483-497.

Basile, G.; Andreano, S.; Martiniello, L.; Mazzitelli, A. (2021). Drivers of performance in a complex environment Holonic approach and Italian business network contracts. *Kybernetes,* 50(7), 2042-2059.

Basole, R.; Park, H.; Chao, R. (2019). Visual Analysis of Venture Similarity in Entrepreneurial Ecosystems. *IEEE Transactions on Engineering Management,* 66(4), 568-582.

Basque, J.; Langley, A. (2018). Invoking Alphonse: The founder figure as a historical resource for organizational identity work. *Organization Studies,* 39(12), 1685-1708.

Batko, R.; Baliga-Nicholson, K. (2019). Digital Innovation as the Key Factor in Changing Organizational Identity into a Digital Organizational Identity. *Problemy Zarzadzania-Management Issues,* 17(4), 39-51.

Batra, S.; Sharma, S. (2017). Stronger may not be better: organizational identity strength and performance of Indian SMEs. *Asia Pacific Journal of Human Resources*, 55(2), 234-254.

Battaglia, M; Zhuo, S.; Frey, M. (2019). Linking inside and outside: identity in crisis situations. *Journal of Organizational Change Management,* 32(4), 457-472.

Battilana, J.; Dorado, S. (2010). Building sustainable hybrid organizations: the case of commercial microfinance organizations. *Academy of Management Journal*, 53(6), 1419-1440.

Battilana, J; Lee, M. (2014). Advancing Research on Hybrid Organizing - Insights from the Study of Social Enterprises. *Academy of Management Annals,* 8(1), 397-441.

Battilana, J.; Sengul, M.; Pache, A.; Model, J. (2015). Harnessing productive tensions in hybrid organizations: the case of work integration social enterprises. *Academy of Management Journal*, 58(6), 1658-1685.

Bayle-Cordier, J.; Mirvis, P.; Moingeon, B. (2015). Projecting Different Identities: A Longitudinal Study of the Whipsaw Effects of Changing Leadership Discourse About the Triple Bottom Line. *Journal of Applied Behavioral Science*, 51(3), 336-374.

Bazin, Y.; Korica, M. (2021). Aesthetic Objects, Aesthetic Judgments and the Crafting of Organizational Style in Creative Industries. *Journal of Management Inquiry, 30(3), 312-330.*

Beck, T.; Plowman, D. (2014). Temporary, Emergent Interorganizational Collaboration in Unexpected Circumstances: A Study of the Columbia Space Shuttle Response Effort. *Organization Science*, 25(4), 1234-1252.

Bednar, J.; Galvin, B.; Ashforth, B.; Hafermalz, E. (2020). Putting Identification in Motion: A Dynamic View of Organizational Identification. *Organization Science,* 31(1), 200-222.

Beebe, C.; Haque, F.; Jarvis, C.; Kenney, M.; Patton, D. (2013). Identity creation and cluster construction: the case of the Paso Robles wine region. *Journal of Economic Geography*, 13(5), 711-740.

Ben-Asher, S. (2019). Teaching and Research: Identity Representations Among Teacher-Education Faculty Members, Decades After an Institutional Change. *Journal of Experimental Education,* 87(4), 680-695*.*

Benbasat, I.; Zmud, R. (2003). The identity crisis within the is discipline: Defining and communicating the discipline's core properties. *MIS Quarterly: Management Information Systems,* 27(2), 183-194.

Bergami, M.; Morandin, G. (2019). Relationship between perceived justice and identification The mediating role of organizational images. *Employee Relations,* 41(1), 176-192.

Besharov, M. (2014). The relational ecology of identification: how organizational identification emerges when individuals hold divergent values. *Academy of Management Journal*, 57(5), 1485-1512.

Beus, J.; Lucianetti, L.; Arthur, W. (2020). Clash of the climates: Examining the paradoxical effects of climates for promotion and prevention. *Personnel Psychology,* 73(2), 241-269.

Bhattacharya, C.; Korschun, D.; Sen, S. (2009). Strengthening stakeholder-company relationships through mutually beneficial corporate social responsibility initiatives. *Journal of Business Ethics*, 85(2), 257-272.

Bhattacharya, C.; Sen, S. (2003). Consumer-company identification: A framework for understanding consumers' relationships with companies. *Journal of Marketing*, 67(2), 76-88.

Bick, G.; Abratt, R.; Bergman, A. (2008). Perceptions of the corporate identity management process in South Africa. *South African Journal of Business Management*, 39(3), 11-20.

Bingham, J.; Dyer, W. Smith, I.; Adams, G. (2011). A Stakeholder Identity Orientation Approach to Corporate Social Performance in Family Firms. *Journal of Business Ethics*, 99(4), 565-585.

Birnholtz, J.; Cohen, M.; Hoch, S. (2007). Organizational character: On the regeneration of Camp Poplar Grove. *Organization Science*, 18(2), 315-332.

Bishop, D.; Eury, J.; Gioia, D.; Trevino, L.; Kreiner, G. (2021). In the heart of a storm: leveraging personal relevance through inside-out research. *Academy of Management Perspectives,* 35(3), 435-460.

Bjorklund, T.; Keipi, T.; Maula, H. (2020). Crafters, explorers, innovators, and co-creators: Narratives in designers' identity work. *Design Studies, 68, 82-112.*

Bligh, M. (2006). Surviving Post-merger 'Culture Clash': Can Cultural Leadership Lessen the Casualties?. *Leadership,* 2(4), 395-426.

Block, J.; Wagner, M. (2014). The Effect of Family Ownership on Different Dimensions of Corporate Social Responsibility: Evidence from Large US Firms. *Business Strategy and The Environment*, 23(7), 475-492.

Bloom, P. (2016). Back to the capitalist future: Fantasy and the paradox of crisis. *Culture and Organization,* 22(2), 158-177.

Boehm, S.; Dwertmann, D.; Bruch, H.; Shamir, B. (2015). The missing link? Investigating organizational identity strength and transformational leadership climate as mechanisms that connect CEO charisma with firm performance. *Leadership Quarterly*, 26(2), 156-171.

Boivie, S.; Lange, D.; McDonald, M.; Westphal, J. (2011). Me or we: the effects of ceo organizational identification on agency costs. *Academy of Management Journal,* 54(3), 551-576.

Bommaraju, R.; Ahearne, M.; Hall, Z.; Tirunillai, S.; Lam, S. (2018). The Impact of Mergers and Acquisitions on the Sales Force. *Journal of Marketing Research,* 55(2), 254-264.

Botero, I.; Thomas, J.; Graves, C.; Fediuk, T. (2013). Understanding multiple family firm identities: An exploration of the communicated identity in official websites. *Journal of Family Business Strategy,* 4(1), 12-21.

Botschen, G.; Promberger, K.; Bernhart, J. (2017). Brand-driven identity development of places. *Journal of Place Management and Development*, 10(2), 152-172.

Botschen, G.; Wegerer, P. (2017). Brand-driven retail format innovation: a conceptual framework. *International Journal of Retail and Distribution Management,* 45(7-8), 874-891.

Bouchikhi, H.; Kimberly, J. (2003). Escaping the identity trap. *MIT Sloan Management Review*, 44(3), 20-26.

Bouchikhi, H.; Kimberly, J. (2017). Paradigmatic warfare: the struggle for the soul of economics at the University of Notre Dame. *Industrial and Corporate Change,* 26(6), 1109-1124.

Boudreau, M.; Serrano, C.; Larson, K. (2014). IT-driven identity work: Creating a group identity in a digital environment. *Information and Organization,* 24(1), 1-24.

Bovers, J.; Hoon, C. (2021). Surviving disruptive change: The role of history in aligning strategy and identity in family businesses. *Journal of Family Business Strategy,* 12(4).

Bowen, S. (2004). Organizational factors encouraging ethical decision making: An exploration into the case of an exemplar. *Journal of Business Ethics*, 52(4), 311-324.

Boxenbaum, E.; Rouleau, L. (2011). New knowledge products as bricolage: metaphors and scripts in organizational theory. *Academy of Management Review,* 36(2), 272-296.

Brannan, M.; Parsons, E.; Priola, V. (2015). Brands at Work: The Search for Meaning in Mundane Work. *Organization Studies,* 36(1), 29-53.

Brannon, D.; Wiklund, J.; Haynie, J. (2013). The Varying Effects of Family Relationships in Entrepreneurial Teams. *Entrepreneurship: Theory and Practice,* 37(1), 107-132.

Brashears, M.; Genkin, M.; Suh, C. (2017). In the Organization's Shadow: How Individual Behavior Is Shaped by Organizational Leakage. *American Journal of Sociology*, 123(3), 787-849.

Bravo, R.; Buil, I.; de Chernatony, L.; Martinez, E. (2017). Managing brand identity: effects on the employees. *International Journal of Bank Marketing,* 35(1), 2-23.

Bravo, R.; Matute, J.; Pina, J. (2016). Corporate identity management in the banking sector: effects on employees' identification, identity attractiveness, and job satisfaction. *Service Business*, 10(4), 687-714.

Bres, L.; Raufflet, E.; Boghossian, J. (2018). Pluralism in Organizations: Learning from Unconventional Forms of Organizations. *International Journal of Management Reviews*, 20(2), 364-386.

Brickson, S. (2013). Athletes, Best Friends, and Social Activists: An Integrative Model Accounting for the Role of Identity in Organizational Identification. *Organization Science,* 24(1), 226-245.

Brilliant, E.; Young, D. (2004). The changing identity of federated community service organizations. *Administration in Social Work,* 28(3-4), 23-46.

Brinkerhoff, J. (2002). Assessing and improving partnership relationships and outcomes: a proposed framework. *Evaluation and Program Planning*, 25(3), 215-231.

Brinkerink, J.; Rondi, E.; Benedetti, C.; Arzubiaga, U. (2020). Family business or business family? Organizational identity elasticity and strategic responses to disruptive innovation. *Journal of Family Business Strategy,* 11(4), 100360.

Brocklehurst, M.; Grey, C.; Sturdy, A. (2010). Management: The work that dares not speak its name. *Management Learning,* 41(1), 7-19.

Brockner, J.; Houser, R.; Birnbaum, G.; Lloyd, K.; Deichter, J.; Nathanson, S.; Rubin, J. (1986). Escalation of commitment to an ineffective course of action - the effect of feedback having negative implications for self-identity. *Administrative Science Quarterly,* 31(1), 109-126.

Bromley, D. (2001). Relationships between personal and corporate reputation. *European Journal of Marketing,* 35(44624), 316-334.

Brown, A. (2001). Organization studies and identity: Towards a research agenda. *Human Relations*, 54(1), 113-121.

Brown, A.; Humphreys, M.; Gurney, P. (2005). Narrative, identity and change: a case study of Laskarina Holidays. *Journal of Organizational Change Management*, 18(4), 312-326.

Brown, M.; Gioia, D. (2002). Making things click - Distributive leadership in an online division of an offline organization. *Leadership Quarterly,* 13(4), 397-419.

Brown, T.; Dacin, P.; Pratt, M.; Whetten, D. (2006). Identity, intended image, construed image, and reputation: An interdisciplinary framework and suggested terminology. *Journal of the Academy of Marketing Science*, 34(2), 99-106.

Browne, L.; Rayner, S. (2015). Managing leadership in university reform: Data-led decision-making, the cost of learning and deja vu?. *Educational Management Administration and Leadership,* 43(2), 290-307.

Brunninge, O. (2009). Using history in organizations How managers make purposeful reference to history in strategy processes. *Journal of Organizational Change Management*, 22(1), 8-26.

Brunton, M. (2018). Managing Change Communication in Contested Professional Spaces*. Journal of Change Management*, 18(1), 35-53.

Brusoni, S.; Rosenkranz, N. (2014). Reading between the lines: Learning as a process between organizational context and individuals' proclivities. *European Management Journal,* 32(1), 147-154.

Bryant, P. (2014). Imprinting by Design: The Microfoundations of Entrepreneurial Adaptation. *Entrepreneurship: Theory and Practice,* 38(5), 1081-1102.

Budych, K.; Helms, T.; Schultz, C. (2012). How do patients with rare diseases experience the medical encounter? Exploring role behavior and its impact on patient-physician interaction. *Health Policy*, 105(2-3), 154-164.

Buffat, A. (2014). Public on the outside, private on the inside': the organizational hybridization, sense of belonging and identity strategies of the employees of a public unemployment insurance fund in Switzerland. *International Review of Administrative Sciences,* 80(1), 70-88.

Bundy, J.; Shropshire, C.; Buchholtz, A. (2013). Strategic cognition and issue salience: toward an explanation of firm responsiveness to stakeholder concerns. *Academy of Management Review*, 38(3), 352-376.

Bush, J. (2020). Win-Win-Lose? Sustainable HRM and the promotion of unsustainable employee outcomes. *Human Resource Management Review,* 30(3), 100676.

Byrd, M. (2018). Diversity Branding Strategy: Concealing Implicit Stereotypes and Biased Behaviors. *Advances in Developing Human Resources*, 20(3), 299-312.

Byun, H.; Kim, T. (2017). Identity Claims and Diffusion of Sustainability Report: Evidence from Korean Listed Companies, 2003-2010. *Journal of Business Ethics*, 140(3), 551-565.

Cabral, C.; Sasidharan, A. (2021). Do regulatory mechanisms affect corporate social performance? Evidence from emerging economies. *Journal of Cleaner Production*, 326, 129383.

Cachon-Rodriguez, G.; Prado-Roman, C.; Blanco-Gonzalez, A. (2021). The relationship between corporate identity and university loyalty: The moderating effect of brand identification in managing an institutional crisis. *Journal of Contingencies and Crisis Management,* 29(3), 265-280.

Cakmakli, A.; Boone, C.; can Witteloostujin, A. (2020). Identity realization, multiple logics and legitimacy: Organizational foundings during the emergence of the Dutch accounting industry. *Accounting, Organizations and Society,* 83.

Caldwell, C. (2009). Identity, Self-Awareness, and Self-Deception: Ethical Implications for Leaders and Organizations. *Journal of Business Ethics*, 90(3), 393-406.

Camillus, J.; Ramanadhan, S.; Ganapathy, K. (2021). Strategy in the time of pandemics, climate change and the Kurzweil Singularity. *Journal of Strategy and Management*, 14(3), 300-314.

Campbell, J. (2015). Identification and Performance Management: An Assessment of Change-Oriented Behavior in Public Organizations. *Public Personnel Management*, 44(1), 46-69.

Canato, A.; Ravasi, D. (2015). Managing long-lasting cultural changes. *Organizational Dynamics*, 44(1), 75-82.

Cannella, A.; Jones, C.; Withers, M. (2015). Family- versus lone-founder-controlled public corporations: social identity theory and boards of directors. *Academy of Management Journal,* 58(2), 436-459.

Canziani, B.; Welsh, D.; Dana, L.; Ramadani, V. (2020). Claiming a family brand identity: The role of website storytelling. *Canadian Journal of Administrative Sciences,* 37(1), 68-81.

Capelos, T.; Provost, C.; Parouti, M.; Barnett, J.; Chenoweth, J.; Fife-Schaw, C.; Kelay, T. (2016). Ingredients of institutional reputations and citizen engagement with regulators. *Regulation and Governance*, 10(4), 350-367.

Cappelen, S.; Pedersen, J. (2021). Hijacked by hope: dynamics of mission drift and identity dilution in a nonprofit organization. *RAE Revista de Administracao de Empresas,* 61(1).

Cardador, M.; Pratt, M. (2018). Becoming who we serve: a model of multi-layered employee-customer identification. *Academy of Management Journal*, 61(6), 2053-2080.

Carlsen, A. (2016). On the tacit side of organizational identity: Narrative unconscious and figured practice. *Culture and Organization,* 22(2), 107-135.

Carmeli, A.; Brammer, S.; Gomes, E.; Tarba, S. (2017). An organizational ethic of care and employee involvement in sustainability-related behaviors: A social identity perspective. *Journal of Organizational Behavior,* 38(9), 1380-1395.

Carmeli, A.; Dothan, A.; Boojihawon, D. (2020). Resilience of sustainability-oriented and financially-driven organizations. *Business Strategy and the Environment,* 29(1), 154-169.

Carmeli, A.; Gilat, G.; Waldman, D. (2007). The role of perceived organizational performance in organizational identification, adjustment and job performance*. *Journal of Management Studies,* 44(6), 972-992.

Carmeli, A.; Shteigman, A. (2010). Top Management Team Behavioral Integration in Small-Sized Firms: A Social Identity Perspective. *Group Dynamics-Theory Research and Practice,* 14(4), 318-331.

Caruso, R.; Di Domizio, M.; Savage, D. (2017). Differences in National Identity, Violence and Conflict in International Sport Tournaments: Hic Sunt Leones!. *Kyklos*, 70(4), 511-545.

Cassar, V.; Bezzina, F.; Buttigieg, S. (2017). The relationship between transformational leadership and work attitudes Comparing mediating influences of social identity and the psychological contract. *Leadership and Organization Development Journal*, 38(5), 646-661.

Cattani, G.; Dunbar, R.; Shapira, Z. (2017). How Commitment to Craftsmanship Leads to Unique Value: Steinway & Sons' Differentiation Strategy. *Strategy Science,* 2(1), 13-38.

Cayla, J.; Penaloza, L. (2012). Mapping the Play of Organizational Identity in Foreign Market Adaptation. *Journal of Marketing*, 76(6), 38-54.

Cechin, A.; Bijman, J.; Pascucci, S.; Omta, O. (2013). Decomposing the Member Relationship in Agricultural Cooperatives: Implications for Commitment. *Agribusiness*, 29(1), 39-61.

Cek, K.; Eyupoglu, S. (2019). Does teachers' perceived corporate social responsibility lead to organisational citizenship behaviour? The mediating roles of job satisfaction and organisational identification. *South African Journal of Business Management,* 50(1), 1-11.

Cetindamar, D. (2018). Designed by law: Purpose, accountability, and transparency at benefit corporations. *Cogent Business and Management*, 5(1), 1423787.

Cetindamar, D.; Ozkazanc-Pan, B. (2017). Assessing mission drift at venture capital impact investors*. Business Ethics,* 26(3), 257-270.

Chang, C.; Chen, Y. (2013). Green organizational identity and green innovation. *Management Decision,* 51(5), 1056-1070.

Chatterjee, D.; Sankaran, B. (2015). Commercializing academic research in emerging economies: Do organizational identities matter?. *Science and Public Policy*, 42(5), 599-613.

Chen, M.; Eweje, G.; Kennedy, J. (2021). Managerial sensemaking of tensions in sustainability: Empirical evidence from Chinese and New Zealand business partnerships. *Journal of Cleaner Production,* 319, 128699.

Chen, N.; Tjosvold, D.; Huang, X.; Xu, D. (2011). Newcomer socialization in China: effects of team values and goal interdependence. *International Journal of Human Resource Management*, 22(16), 3317-3337.

Chen, Y.; Nakazawa, M. (2017). Emotions and Pan-Asian Organizing in the US Southwest: Analyzing Interview Discourses via Sentiment Analysis. *Voluntas*, 28(6), 2785-2806.

Chen, Y.; Gao, L.; Zhang, Y. (2022). The Impact of Green Organizational Identity on Green Competitive Advantage: The Role of Green Ambidexterity Innovation and Organizational Flexibility. *Mathematical Problems in Engineering,* 2022.

Chen, Y. (2011). Green organizational identity: sources and consequence. *Management Decision,* 49(3-4), 384-404.

Cho, S. (2014). An International Organization's Identity Crisis. *Northwestern Journal of International Law and Business,* 34(3), 359-393.

Chorev, N. (2013). Restructuring neoliberalism at the World Health Organization. *Review of International Political Economy*, 20(4), 627-666.

Chreim, S.; Langley, A.; Reay, T.; Comeau-Vallee, M.; Huq, J.-L. (2020). Constructing and sustaining counter-institutional identities. *Academy of Management Journal*, 63(3), 935-964.

Christensen, L. (1995). Buffering organizational identity in the marketing culture. *Organization Studies,* 16(4), 651-672.

Christianson, M.; Farkas, M.; Sutcliffe, K.; Weick, K. (2009). Learning Through Rare Events: Significant Interruptions at the Baltimore & Ohio Railroad Museum. *Organization Science*, 20(5), 846-860.

Ciuchta, M.; Miner, A.; Kim, J.; O'Toole, J. (2018). Founding logics, technology validation, and the path to commercialization. *International Small Business Journal*, 36(3), 307-330.

Ciuchta, M.; O'Toole, J. (2018). Buy local? Organizational Identity in the Localism Movement. *Business and Society,* 57(7), 1481-1497.

Ciuk, S.; Kostera, M. (2010). Drinking from the waters of Lethe: A tale of organizational oblivion. *Management Learning,* 41(2), 187-204.

Clark, E.; Geppert, M. (2011). Subsidiary Integration as Identity Construction and Institution Building: A Political Sensemaking Approach. *Journal of Management Studies*, 48(2), 395-416.

Clark, S.; Gioia, D.; Ketchen, D.; Thomas, J. (2010). Transitional Identity as a Facilitator of Organizational Identity Change during a Merger. *Administrative Science Quarterly,* 55(3), 397-438.

Clegg, S.; Rhodes, C.; Kornberger, M. (2007). Desperately seeking legitimacy: Organizational identity and emerging industries. *Organization Studies,* 28(4), 495-513.

Cloutier, C.; Ravasi, D. (2020). Identity trajectories: explaining long-term patterns of continuity and change in organizational identities. *Academy of Management Journal,* 63(4), 1196-1235.

Cohen-Meitar, R.; Carmeli, A.; Waldman, D. (2009). Linking Meaningfulness in the Workplace to Employee Creativity: The Intervening Role of Organizational Identification and Positive Psychological Experiences. *Creativity Research Journal*, 21(4), 361-375.

Cole, M.; Bruch, H. (2006). Organizational identity strength, identification, and commitment and their relationships to turnover intention: Does organizational hierarchy matter?. *Journal of Organizational Behavior,* 27(5), 585-605.

Coman, C.; Bularca, M.; Repanovici, A. (2021). Constructing and Communicating the Visual Identity of a University. Case Study: Visual Identity of Transilvania University of Brasov. *Sustainability,* 13(13), 7145.

Combes-Joret, M.; Lethielleux, L. (2019). The features and management of identity threats within a nonprofit organization The case of the French Red Cross. *Society and Business Review,* 14(1), 43-62.

Combs, J.; Gentry, R.; Lux, S.; Jaskiewicz, P.; Crook, R. (2020). Corporate Political Activity and Sensitivity to Social Attacks: The Case of Family-Managed Firms. *Family Business Review*, 33(2), 152-174.

Connolly, C.; Hyndman, N.; Liguori, M. (2021). Legitimating accounting change in charities: when values count more than regulation. *Journal of Accounting and Organizational Change,* 17(1), 23-49.

Cooper, K.; Maktoufi, R. (2019). Identity and integration: The roles of relationship and retention in nonprofit mergers. *Nonprofit Management & Leadership,* 30(2), 299-319.

Corley, K.; Harquail, C.; Pratt, M.; Glynn, M.; Fiol, C.; Hatch, M. (2006). Guiding organizational identity through aged adolescence. *Journal of Management Inquiry*, 15(2), 85-99.

Corley, K. (2004). Defined by our strategy or our culture? Hierarchical differences in perceptions of organizational identity and change. *Human Relations*, 57(9), 1145-1177.

Corley, K.; Gioia, D. (2004). Identity ambiguity and change in the wake of a corporate spin-off. *Administrative Science Quarterly,* 49(2), 173-208.

Cornelissen, J.; Akemu, O.; Jonkman, J.; Werner, M. (2021). Building Character: The Formation of a Hybrid Organizational Identity in a Social Enterprise. *Journal of Management Studies,* 58(5), 1294-1330.

Cornelissen, J.; Kafouros, M. (2008). Metaphors and Theory Building in Organization Theory: What Determines the Impact of a Metaphor on Theory?. *British Journal of Management,* 19(4), 365-379.

Cornelissen, J. (2005). Beyond compare: Metaphor in organization theory. *Academy of Management Review*, 30(4), 751-764.

Cornelissen, J. (2002). On the 'Organizational identity' metaphor. *British Journal of Management,* 13(3), 259-268.

Cornelissen, J. (2003). Metaphor as a method in the domain of marketing. *Psychology and Marketing*, 20(3), 209-225.

Cornelius, N.; Wallace, J.; Tassabehji, R. (2007). An analysis of corporate social responsibility, corporate identity and ethics teaching in business schools. *Journal of Business Ethics*, 76(1), 117-135.

Corsten, D.; Gruen, T.; Peyinghaus, M. (2011). The effects of supplier-to-buyer identification on operational performance-An empirical investigation of inter-organizational identification in automotive relationships. *Journal of Operations Management*, 29(6), 549-560.

Corvino, C.; De Leo, A.; Parise, M.; Buscicchio, G. (2022). Organizational Well-Being of Italian Doctoral Students: Is Academia Sustainable When It Comes to Gender Equality? *Sustainability,* 14(11), 6425.

Coskuner, S.; Costur, R.; Bayhan-Karapinar, P.; Metin-Camgoz, S.; Ceylan, S.; Demirtas-Zorbaz, S.; Aktas, E.; Ciffiliz, G. (2018). Mobbing, Organizational Identification, and Perceived Support: Evidence from a *Higher Education* Institution. *Eurasian Journal of Educational Research*, 18(73), 19-40.

Costa, T.; Duarte, H.; Palermo, O. (2014). Control mechanisms and perceived organizational support Exploring the relationship between new and traditional forms of control. *Journal of Organizational Change Management*, 27(3), 407-429.

Costanzo, L.; Vurro, C.; Foster, D.; Servato, F.; Perrini, F. (2014). Dual-Mission Management in Social Entrepreneurship: Qualitative Evidence from Social Firms in the United Kingdom. *Journal of Small Business Management*, 52(4), 655-677.

Coupland, C.; Brown, A. (2004). Constructing organizational identities on the web: A case study of Royal Dutch/Shell. *Journal of Management Studies*, 41(8), 1325-1347.

Cowen, M.; Hodgson, D. (2015). Damaged identities: Examining identity regulation and identity work of Gulf project managers. *International Journal of Project Management*, 33(7), 1523-1533.

Cox, M.; Dickson, G.; Cox, B. (2017). Lifting the veil on allowing headscarves in football: A co-constructed and analytical autoethnography. *Sport Management Review*, 20(5), 522-534.

Cuijpers, M.; Uitdewilligen, S.; Guenter, H. (2016). Effects of dual identification and interteam conflict on multiteam system performance. *Journal of Occupational and Organizational Psychology*, 89(1), 141-171.

Cullinan, J.; Abratt, R.; Mingione, M. (2021). Challenges of corporate brand building and management in a state owned enterprise. *Journal of Product and Brand Management,* 30(2), 293-305.

Cummings, M.; Gamlen, A. (2019). Diaspora engagement institutions and venture investment activity in developing countries. *Journal of International Business Policy,* 2(4), 289-313.

Cunha, M.; Giustiniano, L.; Rego, A.; Clegg, S. (2019). Heaven or Las Vegas: Competing institutional logics and individual experience. *European Management Review,* 16(3), 781-798.

Cunningham, J. (2020). Identifying as an outsider: implications for nonfamily in small family firms. *International Journal of Human Resource Management,* 31(22), 2785-2807.

Cunningham, S.; Cornwell, T.; Coote, L. (2009). Expressing Identity and Shaping Image: The Relationship Between Corporate Mission and Corporate Sponsorship. *Journal of Sport Management*, 23(1), 65-86.

Currie, G.; Finn, R.; Martin, G. (2010). Role Transition and the Interaction of Relational and Social Identity: New Nursing Roles in the English NHS. *Organization Studies,* 31(7), 941-961.

Czinkota, M.; Kaufmann, H.; Basile, G.; Ferri, M. (2020). For-Benefit Company (fBComp): An innovative social-business model. The Italian case. *Journal of Business Research,* 119, 377-387.

Czinkota, M.; Kaufmann, H.; Basile, G. (2014). The relationship between legitimacy, reputation, sustainability and branding for companies and their supply chains. *Industrial Marketing Management*, 43(1), 91-101.

da Silveira, C.; Lages, C.; Simoes, C. (2013). Reconceptualizing brand identity in a dynamic environment. *Journal of Business Research*, 66(1), 28-36.

Dailey, S.; Browning, L. (2014). Retelling stories in organizations: understanding the functions of narrative repetition. *Academy of Management Review*, 39(1), 22-43.

Daniel, J.; Eckerd, A. (2019). Organizational sensegiving: Indicators and nonprofit signaling. *Nonprofit Management & Leadership,* 30(2), 213-231.

Daniel, J.; Galasso, M. (2019). Revenue Embeddedness and Competing Institutional Logics: How Nonprofit Leaders Connect Earned Revenue to Mission and Organizational Identity. *Journal of Social Entrepreneurship,* 10(1), 84-107.

Daniel, J.; Moulton, S. (2017). Beyond Cans and Capacity Nonprofit Roles and Service Network Objectives in an Emergency Food Network. *Nonprofit Management & Leadership,* 28(1), 47-64.

Darmody, M.; Smyth, E. (2018). Religion and primary school choice in Ireland: school institutional identities and student profile. *Irish Educational Studies*, 37(1), 1-17.

Das, G.; Agarwal, J.; Malhotra, N.; Varshneya, G. (2019). Does brand experience translate into brand commitment?: A mediated-moderation model of brand passion and perceived brand ethicality. *Journal of Business Research,* 95, 479-490.

Daskalaki, M (2010). Building 'Bonds' and 'Bridges': Linking Tie Evolution and Network Identity in the Creative Industries. *Organization Studies,* 31(12), 1649-1666.

Davenport, S.; Daellenbach, U. (2011). 'Belonging' to a Virtual Research Centre: Exploring the Influence of Social Capital Formation Processes on Member Identification in a Virtual Organization. *British Journal of Management,* 22(1), 54-76.

Davies, G.; Chun, R.; Kamins, M. (2010). Reputation gaps and the performance of service organizations. *Strategic Management Journal,* 31(5), 530-546.

Dawson, A.; Sharma, P.; Irving, P.; Marcus, J.; Chirico, F. (2015). Predictors of Later-Generation Family Members' Commitment to Family Enterprises. *Entrepreneurship: Theory and Practice,* 39(3), 545-569.

De Bernardis, L.; Giustiniano, L. (2015). Evolution of multiple organisational identities after an M&A event A case study from Europe. *Journal of Organizational Change Management*, 28(3), 333-355.

De Blas, M.; Bourgeon-Renault, D.; Jarrier, E. (2015). Can Interactive Mediation Tools Bridge the Identity Gap Between the Public and the Art Museum?. *International Journal of Arts Management,* 18(1), 52-64.

de Castro Casa Nova, S.; Costa Lourenco, I.; LeitaoAzevedo, R. (2018). The misalignment between accounting faculty perceptions of success and organizational image during a process of institutional change. *International Journal of Organizational Analysis*, 26(5), 812-841.

de la Cruz Deniz-Deniz, M.; Katiuska Cabrera-Suarez, M.; Martin-Santana, J. (2020). Orientation Toward Key Non-family Stakeholders and Economic Performance in Family Firms: The Role of Family Identification with the Firm. *Journal of Business Ethics,* 163(2), 329-345.

de Lange, D. (2013). How do Universities Make Progress? Stakeholder-Related Mechanisms Affecting Adoption of Sustainability in University Curricula. *Journal of Business Ethics*, 118(1), 103-116.

De Massis, A.; Kotlar, J.; Mazzola, P.; Minola, T.; Sciascia, S. (2018). Conflicting Selves: Family Owners' Multiple Goals and Self-Control Agency Problems in Private Firms. *Entrepreneurship: Theory and Practice,* 42(3), 362-389.

De Roeck, K.; Delobbe, N. (2012). Do Environmental CSR Initiatives Serve Organizations' Legitimacy in the Oil Industry? Exploring employees' reactions through organizational identification theory. *Journal of Business Ethics*, 110(4), 397-412.

De Roeck, K.; Maon, F.; Lejeune, C. (2013). Taking Up the Challenge of Corporate Branding: An Integrative Framework. *European Management Review,* 10(3), 137-151.

Degn, L. (2018). Academic sensemaking and behavioural responses - exploring how academics perceive and respond to identity threats in times of turmoil. *Studies In Higher Education*, 43(2), 305-321.

Dejnaka, A.; Kulig-Moskwa, K.; Lobos, K.; Nogiec, J.; Szewczyk, M. (2016). Students' perception of attributes of independent colleges of business profile in the market of higher education in Poland. *Management-Poland*, 20(2), 340-359.

Del Giudice, M.; Della Peruta, M.; Maggioni, V. (2013). One man company or managed succession The transfer of the family dream in southern-Italian firms. *Journal of Organizational Change Management*, 26(4), 703-719.

Demers, C.; Giroux, N.; Chreim, S. (2003). Merger and acquisition announcements as corporate wedding narratives. *Journal of Organizational Change Management*, 16(2), 223-242.

Demil, B. (2020). Reintroducing public actors in entrepreneurial dynamics: A co-evolutionary approach to categorization. *Strategic Entrepreneurship Journal,* 14(1), 43-65.

D'Enbeau, S.; Buzzanell, P. (2013). Constructing a feminist organization's identity in a competitive marketplace: The intersection of ideology, image, and culture. *Human Relations*, 66(11), 1447-1470.

Desai, P. (2017). Values Practices and Identity Sustenance in Dual-identity Organizations. *Journal of Human Values*, 23(1), 1-12.

Devereux, L.; Melewar, T.; Dinnie, K.; Lange, T. (2020). Corporate identity orientation and disorientation: A complexity theory perspective. *Journal of Business Research,* 109, 413-424.

Devinney, T.; Dowling, G.; Perm-Ajchariyawong, N. (2008). The Financial Times business schools ranking: What quality is this signal of quality?. *European Management Review,* 5(4), 195-208.

Dhalla, R.; Oliver, C. (2013). Industry Identity in an Oligopolistic Market and Firms' Responses to Institutional Pressures. *Organization Studies,* 34(12), 1803-1834.

Di Domenico, M. (2015). Evolving Museum Identities and Paradoxical Response Strategies to Identity Challenges and Ambiguities: Changing Ethical Understandings in the Handling of Human Remains. *Journal of Management Inquiry*, 24(3), 300-317.

Dibrell, C.; Marshall, D.; Palar, J.; Gentry, R. (2019). New director selection during growth in family-influenced and lone founder firms: An identity fit perspective. *Journal of Business Research,* 101, 1-11.

Dickel, P. (2018). Exploring the role of entrepreneurial orientation in clean technology ventures. *International Journal of Entrepreneurial Venturing*, 10(1), 56-82.

Dobusch, L.; Schoeneborn, D. (2015). Fluidity, Identity, and Organizationality: The Communicative Constitution of Anonymous. *Journal of Management Studies*, 52(8), 1005-1035.

Doering, H.; Downe, J.; Elraz, H.; Martin, S. (2021). Organizational identity threats and aspirations in reputation management. *Public Management Studies,* 23(3), 376-396.

Dolfsma, W.; Chong-Simandjuntak, L.; Geurts, A. (2017). Reproducing the Firm: Routines, Networks, and Identity. *Journal of Economic Issues*, 51(2), 297-304.

Donia, M..; Sirsly, C.; Ronen, S. (2017). Employee Attributions of Corporate Social Responsibility as Substantive or Symbolic: Validation of a Measure. *Applied Psychology*, 66(1), 103-142.

Dorado, S.; Chen, Y.; Prado, A.; Simon, V. (2022). Attuned HRM Systems for Social Enterprises. *Journal of Business Ethics,* 178(3), 829-848.

Dou, J.; Su, E.; Wang, S. (2019). When Does Family Ownership Promote Proactive Environmental Strategy?. The Role of the Firm's Long-Term Orientation. *Journal of Business Ethics,* 158(1), 81-95.

Dougherty, K..; Huyser, K. (2008). Racially diverse congregations: Organizational identity and the accommodation of differences. *Journal for the Scientific Study of Religion*, 47(1), 23-43.

Dowling, G. (2016). Defining and Measuring Corporate Reputations. *European Management Review,* 13(3), 207-223.

Driver, M. (2009). Struggling with Lack: A Lacanian Perspective on Organizational Identity. *Organization Studies,* 30(1), 55-72.

Droli, M.; Chang, T.; Iseppi, L.; Piccinini, L. (2014). Managing trade contacts in HotRest intermediate markets: a resource-based view analysis in EU countries. *Tourism Economics*, 20(4), 757-778.

Drori, G.; Delmestri, G.; Oberg, A. (2016). The iconography of universities as institutional narratives. *Higher Education,* 71(2), 163-180.

Drori, I.; Wrzesniewski, A.; Ellis, S. (2013). One Out of Many? Boundary Negotiation and Identity Formation in Postmerger Integration. *Organization Science*, 24(6), 1717-1741.

Drumwright, M. (1996). Company advertising with a social dimension: The role of noneconomic criteria. *Journal of Marketing*, 60(4), 71-87.

Duarte, F. (2010). Working with Corporate Social Responsibility in Brazilian Companies: The Role of Managers' Values in the Maintenance of CSR Cultures. *Journal of Business Ethics*, 96(3), 355-368.

Dukerich, J.; Golden, B.; Shortell, S. (2002). Beauty is in the eye of the beholder: The impact of organizational identification identity and image on the cooperative behaviors of physicians. *Administrative Science Quarterly,* 47(3), 507-533.

Dutta, K. (2021). Exploring dynamic capabilities: A systems thinking approach. *Systems Research and Behavioral Science,* 38(1), 125-136.

Dutton, J.; Dukerich, J. (1991). Keeping an eye on the mirror - image and identity in organizational adaptation. *Academy of Management Journal*, 34(3), 517-554.

Dutton, J.; Dukerich, J.; Harquail, C. (1994). Organizational images and member identification. *Administrative Science Quarterly,* 39(2), 239-263.

Ebrahimi, E. (2021). Work-Family Enrichment as a Silver Lining of Work-Family Interface in the Academic Society: Causes and Consequences. *Iranian Journal of Management Studies,* 14(4), 777-798.

Eddleston, K.; Mulki, J. (2021). Differences in Family-Owned SMEs' Ethical Behavior: A Mixed Gamble Perspective of Family Firm Tax Evasion. *Entrepreneurship Theory and Practice,* 45(4), 767-791.

Edman, J. (2016). Cultivating Foreignness: How Organizations Maintain and Leverage Minority Identities. *Journal of Management Studies*, 53(1), 55-88.

Edwards, M. (2009). HR, perceived organisational support and organisational identification: an analysis after organisational formation. *Human Resource Management Journal*, 19(1), 91-115.

Edwards, M.; Edwards, T. (2013). Employee Responses to Changing Aspects of the Employer Brand Following a Multinational Acquisition: A Longitudinal Study. *Human Resource Management*, 52(1), 27-54.

Eger, E. (2021). Co-Constructing Organizational Identity and Culture With Those We Serve: An Ethnography of a Transgender Nonprofit Organization Communicating Family Identity and Identification. *International Journal of Business Communication,* 58(2), 254-281.

Eggers, J.; Park, K. (2018). Incumbent adaptation to technological change: the past, present, and future of research on heterogeneous incumbent response. *Academy of Management Annals,* 12(1), 357-389.

El Ghoul, S.; Guedhami, O.; Wang, H.; Kwok, C. (2016). Family control and corporate social responsibility. *Journal of Banking and Finance*, 73, 131-146.

Elfving, J; Howard, P. (2018). Opportunity identification and identity creation in non-profit organizations. *Journal of Enterprising Communities*, 12(5), 566-581.

Elken, M.; Hovdhaugen, E.; Stensaker, B. (2016). Global rankings in the Nordic region: challenging the identity of research-intensive universities?. *Higher Education*, 72(6), 781-795.

Ellen, P.; Webb, D.; Mohr, L. (2006). Building corporate associations: Consumer attributions for corporate socially responsible programs. *Journal of the Academy of Marketing Science*, 34(2), 147-157.

Elsbach, K. (2003). Organizational perception management. *Research in Organizational Behavior*, 25, 297-332.

Elsbach, K.; Kramer, R. (1996). Members' responses to organizational identity threats: Encountering and countering the Business Week rankings. *Administrative Science Quarterly,* 41(3), 442-476.

Elsbach, K.; Breitsohl, H. (2016). A dual-mode framework of organizational categorization and momentary perception. *Human Relations*, 69(10), 2011-2039.

Elsbach, K.; Cable, D. (2019). Explaining Stakeholder Identification with Moderate Prestige Collectives: A Study of NASCAR Fans. *Organization Studies,* 40(9), 1279-1305.

Elstak, M.; Bhatt, M.; Van Riel, C.; Pratt, M.; Berens, G. (2015). Organizational Identification during a Merger: The Role of Self-Enhancement and Uncertainty Reduction Motives during a Major Organizational Change. *Journal of Management Studies*, 52(1), 32-62.

Empson, L. (2001). Fear of exploitation and fear of contamination: Impediments to knowledge transfer in mergers between professional service firms. *Human Relations*, 54(7), 839-862.

Empson, L. (2004). Organizational identity change: managerial regulation and member identification in an accounting firm acquisition. *Accounting, Organizations and Society*, 29(8), 759-781.

Erat, S.; Kitapci, H.; Akcin, K. (2020). Managerial Perception and Organizational Identity: A Comparative Analysis. *Sustainability,* 12(6), 2278.

Ernst, J.; Schleiter, A. (2021). Organizational Identity Struggles and Reconstruction During Organizational Change: Narratives as symbolic, emotional and practical glue. *Organization Studies,* 42(6), 891-910.

Espino, G. (2022). Rediscovering Epistemic Coalitions Twenty Years Later: Using the International Olympic Committee to Build toward A Literature on Epistemic Institutionalism. *International Studies Review,* 24(3).

Eury, J.; Kreiner, G.; Trevino, L.; Gioia, D. (2018). The past is not dead: legacy identification and alumni ambivalence in the wake of the sandusky scandal at penn state. *Academy of Management Journal*, 61(3), 826-856.

Evans, S. (2015). Defining Distinctiveness: The Connections Between Organizational Identity, Competition, and Strategy in Public Radio Organizations. *International Journal of Business Communication*, 52(1), 42-67.

Ewalt, J.; Ohl, J. (2013). We are still in the desert': Diaspora and the (de)territorialization of identity in discursive representations of the US soldier. *Culture and Organization,* 19(3), 209-226.

Fahrenbach, F.; Kragulj, F. (2020). The ever-changing personality: revisiting the concept of triple-loop learning. *Learning Organization,* 27(6), 499-512.

Fan, D.; Lo, C.; Yeung, A.; Cheng, T. (2018). The impact of corporate label change on long-term labor productivity. *Journal of Business Research*, 86, 96-108.

Fanelli, A.; Misangyi, V. (2006). Bringing out charisma: CEO charisma and external stakeholders. *Academy of Management Review*, 31(4), 1049-1061.

Farrell, C.; Harrison, C.; Coburn, C. (2019). What the Hell Is This, and Who the Hell Are You? Role and Identity Negotiation in Research-Practice Partnerships. *Area Open,* 5(2).

Fauchart, E.; Gruber, M. (2011). Darwinians, communitarians, and missionaries: the role of founder identity in entrepreneurship. *Academy of Management Journal*, 54(5), 935-957.

Fayezi, S.; Zomorrodi, M.; Bals, L. (2018). Procurement sustainability tensions: an integrative perspective. *International Journal of Physical Distribution and Logistics Management*, 48(6), 586-609.

Feldman, M.; Rafaeli, A. (2002). Organizational routines as sources of connections and understandings. *Journal of Management Studies*, 39(3), 309-331.

Felix, B. (2020). Analyzing the formation of a paradoxical organizational identity. *International Journal of Organizational Analysis,* 28(6), 1227-1241.

Ferguson, T.; Deephouse, D.; Ferguson, W. (2000). Do strategic groups differ in reputation?. *Strategic Management Journal,* 21(12), 1195-1214.

Ferrari, F. (2017). The tragedy of having a daughter construction and reproduction of gender stereotypes in italian family SMEs. *Reti, Saperi, Linguaggi: Italian Journal of Cognitive Sciences*, 4(1), 133-151.

Few, W.; Joshi, M. (2013). Top management team decision making: the role of functional and organisational identities on the outcomes of TMT diversity*. European Journal of International Management*, 7(1), 56-73.

Fiol, C. (1991). Managing culture as a competitive resource - an identity-based view of sustainable competitive advantage. *Journal of Management,* 17(1), 191-211.

Fiol, C; Huff, A. (1992). Maps for managers - where are we - where do we go from here. *Journal of Management Studies*, 29(3), 267-285.

Fiol, C.; O'Connor, E. (2002). When hot and cold collide in radical change processes: Lessons from community development. *Organization Science*, 13(5), 532-546.

Fisher, G.; Aguinis, H. (2017). Using Theory Elaboration to Make Theoretical Advancements. *Organizational Research Methods,* 20(3), 438-464.

Fisher, G.; Kotha, S.; Lahiri, A. (2016). Changing with the times: an integrated view of identity, legitimacy, and new venture life cycles*. Academy of Management Review*, 41(3), 383-409.

Fisher, G.; Neubert, E.; Bumell, D. (2021). Resourcefulness narratives: Transforming actions into stories to mobilize support. *Journal of Business Venturing,* 36(4), 106122.

Flammer, C.; Luo, J. (2017). Corporate social responsibility as an employee governance tool: evidence from a quasi-experiment. *Strategic Management Journal,* 38(2), 163-183.

Ford, K.; Cate, L. (2020). The discursive construction of international students in the USA: prestige, diversity, and economic gain. *Higher Education,* 80(6), 1195- 1211.

Foreman, P.; Whetten, D. (2002). Members' identification with multiple-identity organizations. *Organization Science*, 13(6), 618-635.

Foroudi, M.; Balmer, J.; Chen, W. (2020). Explicating place identity attitudes, place architecture attitudes, and identification triad theory. *Journal of Business Research,* 109, 321-336.

Fortwengel, J. (2021). The formation of an MNE identity over the course of internationalization. *Journal of International Business Studies,* 52(6), 1069-1095.

Fosfuri, A.; Giarratana, M.; Roca, E. (2011). Community-focused strategies. *Strategic Organization,* 9(3), 222-239.

Fossestol, K.; Breit, E.; Andreassen, T.; Klemsdal, L. (2015). Managing institutional complexity in public sector reform: hybridization in front-line service organizations. *Public Administration*, 93(2), 290-306.

Fox, S. (2021). Accessing Active Inference Theory through Its Implicit and Deliberative Practice in Human Organizations. *Entropy,* 23(11), 1521.

Fox-Wolfgramm, S.; Boal, K.; Hunt, J. (1998). Organizational adaptation to institutional change: A comparative study of first-order change in prospector and defender banks. *Administrative Science Quarterly,* 43(1), 87-126.

Francis, J.; Zheng, C.; Mukherji, A. (2009). An Institutional Perspective on Foreign Direct Investment. *Management International Review*, 49(5), 565-583.

Frangi, L.; Zhang, T. (2022). Global union federations on affiliates' websites: Forces shaping unions' global organisational identity. *British Journal of Industrial Relations,* 60(2), 444-466.

Frankwick, G.; Ward, J.; Hutt, M.; Reingen, P. (1994). Evolving patterns of organizational beliefs in the formation of strategy. *Journal of Marketing*, 58(2), 96-110.

Fraser, J.; Ansari, S. (2021). Pluralist perspectives and diverse responses: Exploring multiplexed framing in incumbent responses to digital disruption. *Long Range Planning,* 54(5), 102016.

Fritz, J.; Arnett, R.; Conkel, M. (1999). Organizational ethical standards and organizational commitment. *Journal of Business Ethics*, 20(4), 289-299.

Fryzel, B.; Seppala, N. (2016). The effect of CSR evaluations on affective attachment to CSR in different identity orientation firms*. Business Ethics,* 25(3), 310-326.

Fu, J.; Cooper, K. (2022). Multiple Identities in Faith-based Organizations: Exploring Status and Value Homophily in Idealized Partnerships. *Communication Research,* 49(5), 733-759.

Galvin, B.; Lange, D.; Ashforth, B. (2015). Narcissistic organizational identification: seeing oneself as central to the organization's identity. *Academy of Management Review*, 40(2), 163-181.

Garcia, G. (2016). Complicating a Latina/o-serving Identity at a Hispanic Serving Institution. *Review of Higher Education*, 40(1), 117-143.

Garcia, G.; Ramirez, J.; Patron, O.; Cristobal, N. (2019). Constructing an HSI Organizational Identity at Three Hispanic-Serving Institutions in the Midwest: Ideal Versus Current Identity. *Journal of Higher Education*, 90(4), 513-538.

Garcia, P.; Hardy, C. (2007). Positioning, similarity and difference: Narratives of individual and organizational identities in an Australian university. *Scandinavian Journal of Management,* 23(4), 363-383.

Garcia-Lorenzo, L. (2020). Organizational remembering as a trigger for cultural change: Exploring the episodic memories of a financial scandal. *Scandinavian Journal of Management,* 36(1), 101089.

Gehman, J.; Grimes, M. (2017). Hidden badge of honor: how contextual distinctiveness affects category promotion among certified b corporations. *Academy of Management Journal*, 60(6), 2294-2320.

Georgallis, P.; Lee, B. (2020). Toward a theory of entry in moral markets: The role of social movements and organizational identity. *Strategic Organization,* 18(1), 50-74.

Gibney, R.; Zagenczyk, T.; Fuller, J.; Hester, K.; Caner, T. (2011). Exploring Organizational Obstruction and the Expanded Model of Organizational Identification. *Journal of Applied Social Psychology,* 41(5), 1083-1109.

Gibson, M. (2019). The Role of Pride, Shame, Guilt, and Humiliation in Social Service Organizations: A Conceptual Framework from a Qualitative Case Study. *Journal of Social Service Research,* 45(1), 112-128.

Gilad, S. (2011). Institutionalizing fairness in financial markets: Mission impossible?. *Regulation and Governance*, 5(3), 309-332.

Gilad, S. (2015). Political pressures, organizational identity, and attention to tasks: illustrations from pre-crisis financial regulation. *Public Administration*, 93(3), 593-608.

Gilad, S. (2010). Why the Haves Do Not Necessarily Come Out Ahead in Informal Dispute Resolution*. Law and Policy*, 32(3), 283-312.

Gill, M. (2014). The Possibilities of Phenomenology for Organizational Research. *Organizational Research Methods,* 17(2), 118-137.

Gillespie, N.; Dietz, G.; Lockey, S. (2014). Organizational Reintegration and Trust Repair after an Integrity Violation: A Case Study. *Business Ethics Quarterly*, 24(3), 371-410.

Gilpin, D. (2010). Organizational Image Construction in a Fragmented Online Media Environment. *Journal of Public Relations Research,* 22(3), 265-287.

Gilpin, D. (2008). Narrating the organizational self: Reframing the role of the news release. *Public Relations Review*, 34(1), 9-18.

Gilpin, D.; Miller, N. (2013). Exploring Complex Organizational Communities: Identity as Emergent Perceptions, Boundaries, and Relationships. *Communication Theory*, 23(2), 148-169.

Ginsberg, A.; Buchholtz, A. (1990). Converting to for-profit status - corporate responsiveness to radical change. *Academy of Management Journal*, 33(3), 445-477.

Gioia, D.; Chittipeddi, K. (1991). Sensemaking and sensegiving in strategic change initiation. *Strategic Management Journal,* 12(6), 433-448.

Gioia, D.; Schultz, M.; Corley, K. (2000). Organizational identity, image, and adaptive instability. *Academy of Management Review*, 25(1), 63-81.

Gioia, D.; Thomas, J. (1996). Identity, image, and issue interpretation: Sensemaking during strategic change in academia. *Administrative Science Quarterly,* 41(3), 370-403.

Gioia, D.; Patvardhan, S.; Hamilton, A.; Corley, K. (2013). Organizational Identity Formation and Change. *Academy of Management Annals*, 7(1), 123-193.

Gioia, D.; Price, K.; Hamilton, A.; Thomas, J. (2010). Forging an Identity: An Insider-outsider Study of Processes Involved in the Formation of Organizational Identity. *Administrative Science Quarterly,* 55(1), 1-46.

Giorgi, S.; Lockwood, C.; Glynn, M. (2015). The Many Faces of Culture: Making Sense of 30 Years of Research on Culture in Organization Studies. *Academy of Management Annals,* 9(1), 1-54.

Glavas, A.; Godwin, L. (2013). Is the Perception of 'Goodness' Good Enough? Exploring the Relationship Between Perceived Corporate Social Responsibility and Employee Organizational Identification. *Journal of Business Ethics*, 114(1), 15-27.

Glynn, M. (2000). When cymbals become symbols: Conflict over organizational identity within a symphony orchestra. *Organization Science*, 11(3), 285-298.

Glynn, M. (2002). Chord and discord: Organizational crisis, institutional shifts, and the musical canon of the symphony. *Poetics,* 30(1-2), 63-85.

Glynn, M.; Abzug, R. (2002). Institutionalizing identity: Symbolic isomorphism and organizational names. *Academy of Management Journal*, 45(1), 267-280.

Glynn, M.; Barr, P.; Dacin, M. (2000). Pluralism and the problem of variety. *Academy of Management Review*, 25(4), 726-734.

Glynn, M.; Navis, C. (2013). Categories, Identities, and Cultural Classification: Moving Beyond a Model of Categorical Constraint. *Journal of Management Studies*, 50(6), 1124-1137.

Godart, F. (2015). Trend Networks: Multidimensional Proximity and the Formation of Aesthetic Choices in the Creative Economy. *Regional Studies*, 49(6), 973-984.

Golant, B.; Sillince, J.; Harvey, C.; Maclean, M. (2015). Rhetoric of stability and change: The organizational identity work of institutional leadership. *Human Relations*, 68(4), 607-631.

Golden-Biddle, K.; Rao, H. (1997). Breaches in the boardroom: Organizational identity and conflicts of commitment in a nonprofit organization. *Organization Science*, 8(6), 593-611.

Goldring, D. (2015). Reputation orientation Improving marketing performance through corporate reputation building. Marketing Intelligence and Planning, 33(5), 784-803.

Gonzalez, J.; Gulbrandsen, M. (2022). Innovation in established industries undergoing digital transformation: the role of collective identity and public values. *Innovation-Organization & Management,* 24(1), 201-230.

Gonzalez, J.; Chakraborty, S. (2012). Image and similarity: an identity orientation perspective to organizational identification. Leadership and Organization Development Journal, 33(1-2), 51-65.

Goss, K.; Heaney, M. (2010). Organizing Women as Women: Hybridity and Grassroots Collective Action in the 21(st) Century. Perspectives On Politics, 8(1), 27-52.

Grandy, G.; Mavin, S. (2012). Occupational image, organizational image and identity in dirty work: Intersections of organizational efforts and media accounts. *Organization,* 19(6), 765-786.

Grant, A. (2012). Giving time, time after time: work design and sustained employee participation in corporate volunteering. *Academy of Management Review*, 37(4), 589-615.

Greco, A.; Long, T.; de Jong, G. (2021). Identity reflexivity: a framework of heuristics for strategy change in hybrid organizations. *Management Decision,* 59(7), 1684-1705.

Greenwood, R.; Raynard, M.; Kodeih, F.; Micelotta, E.; Lounsbury, M. (2011). Institutional Complexity and Organizational Responses. *Academy of Management Annals*, 5(1), 317-371.

Gregersen, Magnus Kristian; Johansen, Trine Susanne (2018). Corporate visual identity: exploring the dogma of consistency. *Corporate Communications*, 23(3), 342-356.

Greve, W; Strobl, R (2004). Social and individual coping with threats: Outlines of an interdisciplinary approach. Review Of General Psychology, 8(3), 194-207.

Griepentrog, B.; Harold, C..; Holtz, B.; Klimoski, R.; Marsh, S. (2012). Integrating Social Identity and the Theory of Planned Behavior: Predicting Withdrawal from an Organizational Recruitment Process. *Personnel Psychology*, 65(4), 723-753.

Grimes, M. (2010). Strategic Sensemaking Within Funding Relationships: The Effects of Performance Measurement on Organizational Identity in the Social Sector. *Entrepreneurship: Theory and Practice,* 34(4), 763-783.

Grimes, M.; Williams, T.; Zhao, E. (2019). Anchors aweigh: the sources, variety, and challnges of mission drift. *Academy of Management Review,* 44(4), 818-844.

Groggins, A.; Ryan, A. (2013). Embracing uniqueness: The underpinnings of a positive climate for diversity. Journal of Occupational and Organizational Psychology, 86(2), 264-282.

Grunberg, L.; Matei, S. (2020). Why the paradigm of work-family conflict is no longer sustainable: Towards more empowering social imaginaries to understand women's identities. *Gender, Work and Organization*, 27(3), 289-309.

Guiette, A.; Vandenbempt, K. (2013). Exploring team mental model dynamics during strategic change implementation in professional service organizations. A sensemaking perspective. *European Management Journal,* 31(6), 728-744.

Guimaraes-Costa, N.; Pina e Cunha, M. (2008). The Atrium Effect of Website Openness on the Communication of Corporate Social Responsibility. *Corporate Social Responsibility and Environmental Management,* 15(1), 43-51.

Gumport, P. (1993). The contested terrain of academic program reduction. Journal of *Higher Education*, 64(3), 283-311.

Gumz, E. (2008). A Delicate Dance: Identity Issues in a Religious Nonprofit Umbrella Organization. F*amilies in Society-The Journal of Contemporary Social Services*, 89(2), 274-281.

Gunia, B.; Kim, S. (2016). The behavioral benefits of other people's deviance. Group Processes and Intergroup Relations, 19(5), 653-675.

Habemacher, A. (2020). Understanding the Ongoing Dialogues on Indigenous Issues in Canadian Legal Education Through the Lens of Institutional Cultures (Case Studies at UQAM, UAlberta, and UMoncton). *Osgoode Hall Law Journal,* 57(1), 37-97.

Ha-Brookshire, J.; Lu, S. (2010). Organizational Identities and Their Economic Performance: An Analysis of US Textile and Apparel Firms. *Clothing and Textiles Research Journal*, 28(3), 174-188.

Hadida, A.; Paris, T. (2014). Managerial cognition and the value chain in the digital music industry. *Technological Forecasting and Social Change,* 83, 84-97.

Hahl, O.; Ha, J. (2020). Committed Diversification: Why Authenticity Insulates Against Penalties for Diversification. *Organization Science,* 31(1), 1-22.

Hahn, T.; Preuss, L.; Pinkse, J.; Figge, F. (2014). Cognitive frames in corporate sustainability: managerial sensemaking with paradoxical and business case frames. *Academy of Management Review*, 39(4), 463-487.

Hameed, I.; Ijaz, M.; Sabharwal, M. (2022). The Impact of Human Resources Environment and Organizational Identification on Employees' Psychological Well-Being. *Public Personnel Management,* 51(1), 71-96.

Hampel, C.; Tracey, P.; Weber, K. (2020). The art of the pivot: how new ventures manage identification relationships with stakeholders as they change direction. *Academy of Management Journal,* 63(2), 440-471.

Han, E.; Kim, K.; Lee, A. (2019). Contributors to exchange structures and their effects on community solidarity in online communities. *Internet Research,* 29(6), 1410-1442.

Han, Y.; Zheng, E. (2019). Organizational imprinting and the welfare practice of Chinese state-owned enterprises. *Journal of Chinese Sociology,* 6(1), 1-18.

Hanisch, D.; Rau, S. (2014). Application of metric conjoint analysis in family business research. *Journal of Family Business Strategy*, 5(1), 72-84.

Hardy, C.; Lawrence, T.; Grant, D. (2005). Discourse and collaboration: The role of conversations and collective identity. *Academy of Management Review*, 30(1), 58-77.

Harikkala-Laihinen, R. (2022). Hooked on a feeling? An interpretive study of organizational identity (dis) continuity during strategic change programmes. *International Journal of Project Management,* 40(3), 262-277.

Harmel, R.; Tan, A.; Janda, K.; Smith, J. (2018). Manifestos and the two faces of parties: Addressing both members and voters with one document. *Party Politics*, 24(3), 278-288.

Harquail, C.; King, A. (2010). Construing Organizational Identity: The Role of Embodied Cognition. *Organization Studies,* 31(12), 1619-1648.

Harris, M. (2011). Strategic Planning in an International Nongovernmental Development Organization: The Creation of a Meta-Identity. *Administration & Society,* 43(2), 216-247.

Harrison, J.; Wicks, A. (2021). Harmful Stakeholder Strategies. *Journal of Business Ethics,* 169(3), 405-419.

Harvey, W.; Morris, T.; Santos, M. (2017). Reputation and identity conflict in management consulting. *Human Relations*, 70(1), 92-118.

Haslam, S.; Cornelissen, J.; Werner, M. (2017). Metatheories and Metaphors of Organizational Identity: Integrating Social Constructionist, Social Identity, and Social Actor Perspectives within a Social Interactionist Model. *International Journal of Management Reviews*, 19(3), 318-336.

Haslam, S.; Postmes, T.; Ellemers, N. (2003). More than a metaphor: Organizational identity makes organizational life possible. *British Journal of Management,* 14(4), 357-369.

Hatch, M.; Schultz, M. (2002). The dynamics of organizational identity. *Human Relations*, 55(8), 989-1018.

He, H.; Balmer, J. (2013). A grounded theory of the corporate identity and corporate strategy dynamic A corporate marketing perspective. *European Journal of Marketing*, 47(3-4), 401-430.

He, H.; Baruch, Y. (2009). Transforming organizational identity under institutional change. *Journal of Organizational Change Management*, 22(6), 575-599.

He, H.; Baruch, Y. (2010). Organizational Identity and Legitimacy under Major Environmental Changes: Tales of Two UK Building Societies. *British Journal of Management,* 21(1), 44-62.

He, W. (2018). A Fuzzy Evaluation Model for Sustainable Modular Supplier. *Information (Switzerland)*, 9(12), 330.

Heaney, M.; Rojas, F. (2014). Hybrid Activism: Social Movement Mobilization in a Multimovement Environment. *American Journal of Sociology*, 119(4), 1047-1103.

Heckert, R. (2019). Challenges for a Multiple Identity Organization: A Case Study of the Dutch Blood Supply Foundation. *Corporate Reputation Review,* 22(3), 101-119.

Heckert, R.; Boumans, J.; Vliegenthart, R. (2020). How to Nail the Multiple Identities of an Organization? A Content Analysis of Projected Identity. *Voluntas,* 31(1), 129-141.

Heckert, R.; Boumans, J.; Vliegenthart, R. (2021). How Do Media Portray Multiple Identity Organizations?. *International Journal of Communication,* 15, 3025-3046.

Heere, B.; James, J. (2007). Sports teams and their communities: Examining the influence of external group identities on team identity. *Journal of Sport Management*, 21(3), 319-337.

Heide, J.; Wathne, K. (2006). Friends, businesspeople, and relationship roles: A conceptual framework and a research agenda. *Journal of Marketing*, 70(3), 90-103.

Heinzelmann, R. (2018). Occupational identities of management accountants: the role of the IT system. *Journal of Applied Accounting Research*, 19(4), 465-482.

Helms, W.; Patterson, K. (2014). Eliciting acceptance for illicit organizations: the positive implications of stigma for mma organizations. *Academy of Management Journal*, 57(5), 1453-1484.

Hennessy, J.; West, M. (1999). Intergroup behavior in organizations - A field test of social identity theory. *Small Group Research*, 30(3), 361-382.

Heracleous, L.; Jacobs, C. (2008). Understanding organizations through embodied metaphors. *Organization Studies,* 29(1), 45-78.

Hernandez-Linares, R.; Concepcion Lopez-Fernandez, M. (2018). Entrepreneurial Orientation and the Family Firm: Mapping the Field and Tracing a Path for Future Research. *Family Business Review*, 31(3), 318-351.

Herriot, R.; Scott-Jackson, W. (2002). Globalization, social identities and employment. *British Journal of Management,* 13(3), 249-257.

Hertel, C.; Bacq, S.; Belz, F.-M. (2019). It takes a village to sustain a village: a social identity perspective on successful community-based enterprise creation. *Academy of Management Discoveries,* 5(4), 438-464.

Hesar, A.; Abbaszadeh, M.; Ghalei, A.; Ghalavandi, H. (2019). Investigating the relationship between transformational leadership style and organizational identity of faculty members in the state universities of west azerbaijan. *Serbian Journal of Management,* 14(1), 157-176.

Hietajarvi, A.; Aaltonen, K. (2018). The formation of a collaborative project identity in an infrastructure alliance project. *Construction Management and Economics*, 36(1), 1-21.

Himanka, J. (2015). On the Aristotelian origins of higher education. *Higher Education*, 69(1), 117-128.

Himanka, J. (2012). The University as a community of selves: Johan Vilhelm Snellman's On Academic Studies. *Higher Education*, 64(4), 517-528.

Hitt, M.; Keats, B. Harback, H.; Nixon, R. (1994). Rightsizing - building and maintaining strategic leadership and long-term competitiveness. *Organizational Dynamics*, 23(2), 18-32.

Hoffman, A.; Ocasio, W. (2001). Not all events are attended equally: Toward a middle-range theory of industry attention to external events. *Organization Science*, 12(4), 414-434.

Hoffmann, J.; Ramirez, R.; Lecamp, L. (2018). Right on time - Socioecological strategy and implications of turbulence in the Swiss watchmaking field. *Technological Forecasting and Social Change,* 137, 101-117.

Hogstrom, C.; Gustafsson, A.; Tronvoll, B. (2015). Strategic brand management: Archetypes for managing brands through paradoxes. *Journal of Business Research*, 68(2), 391-404.

Hong, J.; Fiona, K. (2009). Conflicting Identities and Power Between Communities of Practice: The Case of IT Outsourcing. *Management Learning,* 40(3), 311-326.

Hoobler, J. (2007). On-site or out-of-sight? - Family-friendly child care provisions and the status of working mothers. *Journal of Management Inquiry*, 16(4), 372-380.

Hoon, C.; Jacobs, C. (2014). Beyond belief: Strategic taboos and organizational identity in strategic agenda setting. *Strategic Organization,* 12(4), 244-273.

Horng, C.; Huarng, F. (2002). TQM adoption by hospitals in Taiwan. *Total Quality Management and Business Excellence*, 13(4), 441-463.

Horng, C.; Chen, W. (2008). From contract manufacturing to own brand management: The role of learning and cultural heritage identity. *Management and Organization Review*, 4(1), 109-133.

Horst, M. (2013). A Field of Expertise, the *Organization,* or Science Itself? Scientists' Perception of Representing Research in Public Communication. *Science Communication,* 35(6), 758-779.

Horton, K.; Bayerl, P.; Jacobs, G. (2014). Identity conflicts at work: An integrative framework. *Journal of Organizational Behavior,* 35(1), S6-S22.

Horton, K.; Wanderley, C. (2018). Identity conflict and the paradox of embedded agency in the management accounting profession: Adding a new piece to the theoretical jigsaw. *Management Accounting Research*, 38, 39-50.

Hoskins, J.; Brown, B. (2017). Hold firm or adapt? An empirical examination of the institutional appeal implications of maintaining the Liberal Arts College identity. *Journal of Marketing* for *Higher Education*, 27(2), 188-212.

Hou, Z.; Zhu, Y. (2012). An institutional perspective of public relations practices in the Chinese cultural contexts. *Public Relations Review*, 38(5), 916-925.

Hou, Z.; Zhu, Y.; Bromley, M. (2013). Understanding Public Relations in China: Multiple Logics and Identities. *Journal of Business and Technical Communication,* 27(3), 308-328.

Houghton, S.; Gabel, J.; Williams, D. (2009). Connecting the Two Faces of CSR: Does Employee Volunteerism Improve Compliance?. *Journal of Business Ethics*, 87(4), 477-494.

Howard-Grenville, J.; Metzger, M.; Meyer, A. (2013). Rekindling the flame: processes of identity resurrection. *Academy of Management Journal*, 56(1), 113-136.

Hsieh, Y.; Weng, J.; Lin, T. (2018). How social enterprises manage their organizational identification: a theoretical framework of identity management approach through attraction, selection, and socialization. *International Journal of Human Resource Management*, 29(20), 2880-2904.

Hsu, G.; Hannan, M. (2005). Identities, genres, and organizational forms. *Organization Science*, 16(5), 474-490.

Hsu, G. (2006). Jacks of all trades and masters of none: Audiences' reactions to spanning genres in feature film production. *Administrative Science Quarterly,* 51(3), 420-450.

Hsu, G.; Elsbach, K. (2013). Explaining Variation in Organizational Identity Categorization. *Organization Science*, 24(4), 996-1013.

Hsu, G.; Kocak, O.; Kovacs, B. (2018). Co-Opt or Coexist? A Study of Medical Cannabis Dispensaries' Identity-Based Responses to Recreational-Use Legalization in Colorado and Washington. *Organization Science*, 29(1), 172-190.

Huang, M.; Chang, B. (2019). Sleeping with the enemy: The moderating effect of cooperation on competitive dynamics from collective identity perspective. *Management Decision,* 57(1), 211-228.

Huang-Horowitz, N. (2015). Public relations in the small business environment: Creating identity and building reputation. *Public Relations Review*, 41(3), 345-353.

Huang- Horowitz, N.; Evans, S. (2020). Communicating Organizational Identity as Part of the Legitimation Process: A Case Study of Small Firms in an Emerging Field. *International Journal of Business Communication,* 57(3), 327-351.

Huber, K.; Schormair, M. (2021). Progressive and Conservative Firms in Multistakeholder Initiatives: Tracing the Construction of Political CSR Identities Within the Accord on Fire and Building Safety in Bangladesh. *Business and Society,* 60(2), 454-495.

Hudson, B. (2008). Against all odds: A consideration of core-stigmatized organizations. *Academy of Management Review*, 33(1), 252-266.

Huemer, L. (2010). Corporate Social Responsibility and Multinational Corporation Identity: Norwegian Strategies in the Chilean Aquaculture Industry. *Journal of Business Ethics*, 91(2), 265-277.

Huemer, L. (2013). When in Rome, be(come) a Roman? Building organizational identitites in networks. *Industrial Marketing Management*, 42(7), 1112-1120.

Huettermann, H.; Doering, S.; Boerner, S. (2017). Understanding the Development of Team Identification: A Qualitative Study in UN Peacebuilding Teams. *Journal of Business and Psychology*, 32(2), 217-234.

Huhtala, M; Tolvanen, A.; Mauno, S.; Feldt, T. (2015). The Associations between Ethical Organizational Culture, Burnout, and Engagement: A Multilevel Study. *Journal of Business and Psychology*, 30(2), 399-414.

Huikkola, T.; Raetino, R.; Kohtamaki, M.; Gebauer, H. (2020). Firm boundaries in servitization: Interplay and repositioning practices. *Industrial Marketing Management,* 90, 90-105.

Hurst, R.; Pattath, P. (2019). Organizational identity in acculturation in cross-border acquisitions: Implications for HRD practitioners in global M&A. *Human Resource Development International,* 22(1), 44-67.

Husmann, I.; Kleinaltenkamp, M.; Hanmer-Lloyd, S. (2020). Aligning resource integration and organizational identities in project networks. *Journal of Business and Industrial Marketing,* 35(10), 1581-1589.

Hussenot, A. (2021). All for One, One for All! From Events to Organizational Dynamics in Fluid Organization. *Management,* 24(2), 1-22.

Hussenot, A.; Missonier, S. (2016). Encompassing Stability and Novelty in Organization Studies: An Events-based Approach. *Organization Studies,* 37(4), 523-546.

Huy, Q. (1999). Emotional capability, emotional intelligence, and radical change. *Academy of Management Review*, 24(2), 325-345.

Huzzard, T.; Hellstrom, A.; Lifvergren, S. (2018). Whole System in the Room: Toward Systems Integration in Healthcare. *Health Communication*, 33(7), 800-808.

Hwang, J.; Han, H. (2015). Understanding Other Customer Perceptions in the Private Country Club Industry. *Asia Pacific Journal of Tourism Research*, 20(8), 875-896.

Hwang, J.; Han, H. (2017). Are other customer perceptions important at casino table games? Their impact on emotional responses and word-of-mouth by gender. *Journal of Travel and Tourism Marketing*, 34(4), 544-555.

Iannone, F.; Izzo, F. (2017). Salvatore Ferragamo: An Italian heritage brand and its museum*. Place Branding and Public Diplomacy*, 13(2), 163-175.

Ibanez, M.; Alonso Dos Santos, M.; Llanos-Contreras, O. (2022). Transmission of family identity and consumer response: do consumers recognize family firms?. *International Journal of Entrepreneurial Behaviour and Research,* 28(1), 6-25.

Illia, L. (2009). Exploring How to Diagnose Members' Concerns About Changes in Core Elements of Organizations. *Journal of Applied Behavioral Science*, 45(4), 550-580.

Irwin, J.; Lahneman, B.; Parmigiani, A. (2018). Nested identities as cognitive drivers of strategy. *Strategic Management Journal,* 39(2), 269-294.

Islam, G. (2014). Identities and ideals: Psychoanalytic dialogues of self and leadership. *Leadership,* 10(3), 344-360.

Ismail, A.; Johnson, B. (2019). Managing Organizational Paradoxes in Social Enterprises: Case Studies from the MENA Region. *Voluntas,* 30(3), 516-534.

Jabri, M. (2009). Promoting exchange between East and West management cultures: The role of dialogue. *Journal of Management and Organization,* 15(4), 514-525.

Jack, G.; Lorbiecki, A. (2007). National identity, globalization and the discursive construction of organizational identity. *British Journal of Management,* 18(1), S79-S94.

Jacobides, M.; Winter, S. (2005). The co-evolution of capabilities and transaction costs: Explaining the institutional structure of production. *Strategic Management Journal,* 26(5), 395-413.

Jacobs, C.; Kreutzer, K.; Vaara, E. (2021). Political dynamics in organizational identity breach and reconstruction: findings from the crisis in UNICEF germany. *Academy of Management Journal,* 64(3), 948-980.

Jacobs, C.; Oliver, D.; Heracleous, L. (2013). Diagnosing Organizational Identity Beliefs by Eliciting Complex, Multimodal Metaphors. *Journal of Applied Behavioral Science*, 49(4), 485-507.

Jaeger, U.; Schroeer, A. (2014). Integrated Organizational Identity: A Definition of Hybrid Organizations and a Research Agenda. *Voluntas,* 25(5), 1281-1306.

Jancenelle, V., Javalgi, R.; Cavusgil, E. (2018). The role of economic and normative signals in international prosocial crowdfunding: An illustration using market orientation and psychological capital. *International Business Review,* 27(1), 208-217.

Janssen, A.; Beers, P.; van Mierlo, B. (2022). Identity in sustainability transitions: The crucial role of landscape in the Green Heart. *Environmental Innovation and Societal transitions,* 42, 362-373.

Jansson, J. (2013). From movement to organization: constructing identity in Swedish trade unions. *Labor History,* 54(3), 301-320.

Jansson, J. (2022). Re-inventing the self: Implications of trade union revitalization. *Economic and Industrial Democracy,* 43(1), 450-468.

Jay, J. (2013). Navigating paradox as a mechanism of change and innovation in hybrid organizations. *Academy of Management Journal*, 56(1), 137-159.

Jeffrey, L.; Brunton, M. (2012). Professional identity: How communication management practitioners identify with their industry. *Public Relations Review*, 38(1), 156-158.

Jensen, M.; Kim, B. (2014). Great, madama butterfly again! How robust market identity shapes opera repertoires. *Organization Science*, 25(1), 109-126.

Jezierska, K. (2021). Dangling in a Vacuum: A Presentation of Polish Think Tanks in Political Life. *East European Politics and Societies,* 35(3), 812-836.

Jian, G. (2011). Articulating circumstance, identity and practice: toward a discursive framework of organizational changing. *Organization,* 18(1), 45-64.

Jiang, L.; Jiang, D.; Grover, V. (2017). The Sphere of Influence of Information Systems Journals: A Longitudinal Study. *Communications of the Association for Information Systems,* 41, 150-178.

Jing, R.; Van de Ven, A. (2016). Being versus becoming ontology of paradox management. *Cross Cultural and Strategic Management*, 23(4), 558-562.

Joachim, J.; Martin, M.; Lange, H.; Schneiker, A.; Dau, M. (2018). Twittering for talent: Private military and security companies between business and military branding. *Contemporary Security Policy*, 39(2), 298-316.

Joachim, J.; Schneiker, A. (2018). Humanitarian NGOs as Businesses and Managers: Theoretical Reflection on an Under-Explored Phenomenon. *International Studies Perspectives*, 19(2), 170-187.

Johnson, S.; Ashforth, B. (2008). Externalization of employment in a service environment: the role of organizational and customer identification. *Journal of Organizational Behavior,* 29(3), 287-309.

Johnson, T.; Jian, G. (2017). Understanding Organizational-Identity Development Across the Lifecycle in an Emerging Faith-Based Organization: A Case Analysis. *Southern Communication Journal*, 82(3), 185-197.

Johnson, V. (2012). Identity, Sustainability, and Local Setting at US Botanical Gardens. *Organization and Environment*, 25(3), 259-285.

Johnson, W.; Johnson, A.; Heimberg, F. (1999). A primary- and second-order component analysis of the organizational identification questionnaire. *Educational and Psychological Measurement*, 59(1), 159-170.

Jones, C.; Volpe, E. (2011). Organizational identification: Extending our understanding of social identities through social networks. *Journal of Organizational Behavior,* 32(3), 413-434.

Jose Parada, M.; Dawson, A. (2017). Building family business identity through transgenerational narratives. *Journal of Organizational Change Management*, 30(3), 344-356.

Kalaignanam, K.; Bahadir, S. (2013). Corporate brand name changes and business restructuring: is the relationship complementary or substitutive?. *Journal of the Academy of Marketing Science*, 41(4), 456-472.

Kaltcheva, V.; Parasuraman, A. (2009). Personality-Relatedness and Reciprocity framework for analyzing retailer-consumer interactions. *Journal of Business Research*, 62(6), 601-608.

Kammerlander, N.; Koenig, A.; Richards, M. (2018). Why Do Incumbents Respond Heterogeneously to Disruptive Innovations? The Interplay of Domain Identity and Role Identity. *Journal of Management Studies*, 55(7), 1122-1165.

Karreman, D.; Alvesson, M. (2004). Cages in tandem: Management control, social identity, and identification in a knowledge-intensive firm. *Organization,* 11(1), 149-175.

Karreman, D.; Alvesson, M. (2001). Making newsmakers: Conversational identity at work. *Organization Studies,* 22(1), 59-89.

Kartalis, N; Tsamenyi, M.; Jayasinghe, K. (2016). Accounting in new public management (NPM) and shifting organizational boundaries Evidence from the Greek Show Caves. *Accounting Auditing & Accountability Journal*, 29(2), 248-277.

Karthikeyan, S.; Jonsson, S.; Wezel, F. (2016). The Travails of Identity Change: Competitor Claims and Distinctiveness of British Political Parties, 1970-1992. *Organization Science*, 27(1), 106-122.

Kasper-Fuehrer, E.; Ashkanasy, N. (2001). Communicating trustworthiness and building trust in interorganizational virtual organizations. *Journal of Management,* 27(3), 235-254.

Keers, B.; van Fenema, P.; Zijm, H. (2017). Understanding organizational change for alliancing. *Journal of Organizational Change Management*, 30(5), 823-838.

Keller, R.; Ollig, P.; Roevekamp, P. (2022). Pathways to Developing Digital Capabilities within Entrepreneurial Initiatives in Pre-Digital Organizations A Single Case Study. *Business and Information Systems Engineering,* 64(1), 33-46.

Kemendi, T.; Tutusaus, M. (2018). The impact of pro-poor interventions on the performance indicators of a water utility: case studies of Nakuru and Kisumu. *Journal of Water Sanitation and Hygiene for Development*, 8(2), 208-216.

Ketchen, D.; Hult, G. (2011). Building theory about supply chain management: some tools from the organizational sciences. *Journal of Supply Chain Management*, 47(2), 12-18.

Ketokivi, M. (2019). Avoiding bias and fallacy in survey research: A behavioral multilevel approach. *Journal of Operations Management,* 65(4), 380-402.

Ketokivi, M.; Mantere, S.; Cornelissen, J. (2017). Reasoning by analogy and the progress of theory. *Academy of Management Review*, 42(4), 637-658.

Khessina, O.; Reis, S.; Verhaal, J. (2021). Stepping out of the Shadows: Identity Exposure as a Remedy for Stigma Transfer Concerns in the Medical Marijuana Market. *Administrative Science Quarterly,* 66(3), 569-611.

Khorshid, S.; Mehdiabadi, A. (2021). Effect of organizational identification on organizational innovativeness in universities and higher education institutions of Iran, mediated by risk-taking capability. *European Journal of Innovation Management,* 24(4), 1430-1458.

Ki, E.; Pasadeos, Y.; Ertem-Eray, T. (2019). Growth of public relations research networks: a bibliometric analysis. *Journal of Public Relations Research,* 31(1-2), 5-31.

Kiernan, M.; Repper, J.; Arthur, A. (2015). Why do they fail? A qualitative follow up study of 1000 recruits to the British Army Infantry to understand high levels of attrition. *Work-A Journal of Prevention Assessment and Rehabilitation*, 52(4), 921-934.

Kim, B.; Jensen, M. (2011). How Product Order Affects Market Identity: Repertoire Ordering in the U.S. Opera Market. *Administrative Science Quarterly,* 56(2), 238-256.

Kim, C.; Lee, S.; Kang, M. (2012). I became an attractive person in the virtual world: Users' identification with virtual communities and avatars. *Computers in Human Behavior*, 28(5), 1663-1669.

Kim, D. (2018). Examining effects of internal public relations practices on organizational social capital in the Korean context: Mediating roles of employee-organization relationships. *Corporate Communications*, 23(1), 100-116.

Kim, J.; Cameron, G. (2016). When Cousins Feud: Advancing Threat Appraisal and Contingency Theory in Situations That Question the Essential Identity of Activist Organizations. *International Journal of Communication*, 10, 1934-1949.

Kim, J.; Cameron, G. (2015). When groups in glass houses throw stones: Public expectations of how activist groups respond to identity threats. *Social Science Journal*, 52(2), 131-140.

Kim, J. (2020). Halos and Egos: Rankings and Interspecialty Deference in Multispecialty US Hospitals. *Management Science,* 66(5), 2248-2268.

Kim, J.; Song, E.; Lee, S. (2013). Organizational change and employee organizational identification: mediation of perceived uncertainty. Social Behavior and Personality, 41(6), 1019-1034.

Kim, K.; Ko, D. (2020). How to Build a Sustainable MICE Environment Based on Social Identity Theory. *Sustainability,* 12(17).

King, B.; Clemens, E.; Fry, M. (2011). Identity Realization and Organizational Forms: Differentiation and Consolidation of Identities Among Arizona's Charter Schools. *Organization Science*, 22(3), 554-572.

Kitay, J.; Wright, C. (2007). From prophets to profits: The occupational rhetoric of management consultants. *Human Relations*, 60(11), 1613-1640.

Kjaergaard, A.; Morsing, M.; Ravasi, D. (2011). Mediating Identity: A Study of Media Influence on Organizational Identity Construction in a Celebrity Firm. *Journal of Management Studies*, 48(3), 514-543.

Keimann, B. (2019), (German) Universities as multiple hybrid organizations. *Higher Education,* 77(6), 1085-1102.

Kloser, M.; Wilsey, M.; Hopkins, D.; Dallavis, J.; Lavin, E.; Comuniello, M. (2018). Dual identities: organizational negotiation in STEM-focused Catholic schools. *Cultural Studies of Science Education*, 13(2), 549-579.

Knight, C.; Haslam, S. (2010). Your Place or Mine? Organizational Identification and Comfort as Mediators of Relationships Between the Managerial Control of Workspace and Employees' Satisfaction and Well-being. *British Journal of Management,* 21(3), 717-735.

Knox, S.; Freeman, C. (2006). Measuring and Managing Employer Brand Image in the Service Industry. *Journal of Marketing* Management, 22(7-8), 695-716.

Ko, S.; Moon, T.; Hur, W. (2018). Bridging Service Employees' Perceptions of CSR and Organizational Citizenship Behavior: The Moderated Mediation Effects of Personal Traits. *Current Psychology*, 37(4), 816-831.

Kociatkiewicz, J.; Kostera, M. (2010). Experiencing the Shadow: Organizational Exclusion and Denial within Experience Economy. *Organization,* 17(2), 257-282.

Koenig, A.; Graf-Vlachy, L.; Schoeberl, M. (2021). Opportunity/Threat Perception and Inertia in Response to Discontinuous Change: Replicating and Extending Gilbert (2005)*. Journal of Management,* 47(3), 771-816.

Koenig, A.; Schulte, M.; Enders, A. (2012). Inertia in response to non-paradigmatic change: The case of meta-organizations. *Research Policy*, 41(8), 1325-1343.

Kohtamaki, M.; Thorgren, S.; Wincent, J. (2016). Organizational identity and behaviors in strategic networks. *Journal of Business and Industrial Marketing*, 31(1), 36-46.

Kopaneva, I. (2021), Discursive Strategies of Organizational Identity Formation in Benefit Corporations: Coping with a Meanings Void and Assimilating External Feedback. *Western Journal of Communication,* 85(1), 22-41.

Koritos, C.; Koronios, K.; Stathakopoulos, V. (2014). Functional vs relational benefits: what matters most in affinity marketing?. *Journal of Services Marketing*, 28(4), 265-275.

Korschun, D. (2015). Boundary-spanning employees and relationships with external stakeholders: a social identity approach. *Academy of Management Review*, 40(4), 611-629.

Koschmann, M. (2013). The Communicative Constitution of Collective Identity in Interorganizational Collaboration. *Management Communication Quarterly,* 27(1), 61-89.

Koschmann, M.; Kuhn, T.; Pfarrer, M. (2012). A communicative framework of value in cross-sector partnerships. *Academy of Management Review*, 37(3), 332-354.

Koskela-Huotari, K.; Siltaloppi, J. (2020). Rethinking the actor in service research: toward a processual view of identity dynamics. *Journal of Service Theory and Practice,* 30(4-5), 437-457.

Kotarba, J.; Wooten, K. (2017). The innovation scorecard for continuous improvement applied to translational science. *Journal of Clinical and Translational Science,* 1(5), 296-300.

Kourti, I. (2021). Managing the identity paradox in inter-organisational collaborations. *European Management Review,* 18(4), 445-459.

Kovoor-Misra, S. (2002). Boxed-in: Top managers' propensities during crisis issue diagnosis. *Technology Forecasting and Social Change,* 69(8), 803-817.

Kovoor-Misra, S. (2009). Understanding perceived organizational identity during crisis and change A threat/opportunity framework. *Journal of Organizational Change Management*, 22(5), 494-510.

Kovoor-Misra, S.; Smith, M. (2008). In the Aftermath of an Acquisition Triggers and Effects on Perceived Organizational Identity. *Journal of Applied Behavioral Science*, 44(4), 422-444.

Kreiner, G.; Hollensbe, E.; Sheep, M. (2006). On the edge of identity: Boundary dynamics at the interface of individual and organizational identities. *Human Relations*, 59(10), 1315-1341.

Kreiner, G.; Hollensbe, E.; Sheep, M.; Smith, B.; Kataria, N. (2015). Elasticity and the dialectic tensions of organizational identity: how can we hold together while we are pulling apart?. *Academy of Management Journal*, 58(4), 981-1011.

Krishnan, G.; Peytcheva, M. (2019). The Risk of Fraud in Family Firms: Assessments of External Auditors. *Journal of Business Ethics,* 157(1), 261-278.

Kroon, D.; Noorderhaven, N. (2018). The Role of Occupational Identification During Post-Merger Integration. *Group and Organization Management,* 43(2), 207-244.

Kryscynski, D.; Coff, R.; Campbell, B. (2021). Charting a path between firm-specific incentives and human capital-based competitive advantage. *Strategic Management Journal,* 42(2), 386-412.

Kumar, R.; Usunier, J. (2001). Management education in a globalizing world - Lessons from the French experience. *Management Learning,* 32(3), 363-391.

Kump, B. (2019). Beyond Power Struggles: A Multilevel Perspective on Incongruences at the Interface of Practice, Knowledge, and Identity in Radical Organizational Change. *Journal of Applied Behavioral Science,* 55(1), 5-26.

Kuppelwieser, V.; Klaus, P; Baruch, Y.; Manthiou, A. (2018). The missing link: Fairness as the ultimate determinant of service profitability?!. *Recherche et Applications en Marketing*, 33(2), 46-74.

Laari-Salmela, S.; Mainela, T.; Puhakka, V. (2019). Resolving the start-up identity crisis: Strategizing in a network context. *Industrial Marketing Management,* 80, 201-213.

Labianca, G.; Fairbank, J.; Thomas, J.; Gioia, D.; Umphress, E. (2001). Emulation in academia: Balancing structure and identity. *Organization Science*, 12(3), 312-330.

Labianca, G.; Gray, B.; Brass, D. (2000). A grounded model of organizational schema change during empowerment. *Organization Science*, 11(2), 235-257.

Labianca, G.; Fairbank, J. (2005). Interorganizational monitoring: Process, choices, and outcomes. Strategy Process: Advances in Strategic Management, 22, 117-150.

Lahdesmaki, M.; Siltaoja, M. (2010). Towards a Variety of Meanings - Multiple Representations of Reputation in the Small Business Context. *British Journal of Management,* 21(1), 207-222.

Lahiri, M.; Bhandarker, A.; Behrens, A. (2021). Organizational memory and institution theory: A postcolonial perspective. *Thunderbird International Business Review,* 63(4), 487-501.

Lai, J.; Chan, K.; Lam, L. (2013). Defining who you are not: The roles of moral dirtiness and occupational and organizational disidentification in affecting casino employee turnover intention. *Journal of Business Research*, 66(9), 1659-1666.

Lai, K.; Morgan, G.; Morris, J. (2020). Eating Bitterness' in a Chinese Multinational: Identity Regulation in Context. *Organizational Studies,* 41(5), 661-680.

Lamertz, K.; Heugens, P.; Calmet, L. (2005). The configuration of organizational images among firms in the Canadian beer brewing industry. *Journal of Management Studies*, 42(4), 817-843.

Lamertz, K. (2022). Brewing a Craft Impression: A Multilevel Study About the Orchestration of Organizational Impression Management Through Authenticity. *Group and Organization Management,* 47(1), 3-40.

Lamertz, K.; Foster, W.; Coraiola, D.; Kroezen, J. (2016). New identities from remnants of the past: an examination of the history of beer brewing in Ontario and the recent emergence of craft breweries. *Business History,* 58(5), 796-828.

Landau, D.; Drori, I.; Porras, J. (2006). Vision Change in a Governmental R&D Organization The Pioneering Legacy as an Enduring Element. *Journal of Applied Behavioral Science*, 42(2), 145-171.

Langer, C.; Anderson, D.; Furman, R.; Blue, J. (2006). Building and marketing an image in child welfare. *Journal of Organizational Change Management*, 19(3), 307-317.

Langer, J. (2022). Understanding the Spirit of the Sectors: Exploring Identity in a New Era of Organizing. *Administration & Society,* 54(5), 792-827.

Langley, A.; Denis, J.; Lamothe, L. (2003). Process research in healthcare: towards three-dimensional learning. Policy and Politics, 31(2), 195-206.

Langner, B.; Seidel, V. (2015). Sustaining the Flow of External Ideas: The Role of Dual Social Identity across Communities and Organizations. *Journal of Product Innovation Management,* 32(4), 522-538.

Lauring, J.; Thomsen, C. (2009). Collective Ideals and Practices in Sustainable Development: Managing Corporate Identity. *Corporate Social Responsibility and Environmental Management,* 16(1), 38-47.

Lausch, D.; Teman, E.; Perry, C. (2017). Scholastics, Pabulum, Clans, Transformation: A Journey into Otherness. *Journal of International Students*, 7(3), 893-917.

Laux, T. (2019). How do think tanks qualify their expertise? Exploring the field of scientific policy advice in France. *Journal of Science Communication,* 18(3).

Laviolette, E.; Arcand, S.; Cloutier, L.; Renard, L. (2022). Same but Different: Meta-Organization and Collective Identity Dynamics. *Management,* 25(2), 45-59.

Lawrence, T. (2004). Rituals and resistance: Membership dynamics in professional fields. *Human Relations*, 57(2), 115-143.

Lee, L.; Zhong, W. (2020). Opportunism, Identification Asymmetry, and Firm Performance in Chinese Interorganizational Relationships. *Management Organization Review,* 16(4), 825-865.

Lee, L.; Zhong, W. (2020). Run away or stick together: the impact of firm misbehaviour on alliance partners' defection in China. *Asia Pacific Business Review,* 26(5), 663-689.

Lee, P. (2001). What's in a name.com?: The effects of '.com' name changes on stock prices and trading activity. *Strategic Management Journal,* 22(8), 793-804.

Lee, P.; Lau, K. (2019). From an idea generator to a solution facilitator A study of the changing roles of advertising professionals in the social media marketing era. *Career Development International,* 24(1), 2-17.

Lee, S.; Lee, T.; Lum, C. (2008). The effects of employee services on organizational commitment and intentions to quit. *Personnel Review*, 37(1-2), 222-237.

Lee, S.; Phan, P.; Ding, H. (2016). A theory of family employee involvement during resource paucity. *Journal of Family Business Strategy*, 7(3), 160-166.

Lee, Z.; Bourne, H. (2017). Managing Dual Identities in Nonprofit Rebranding: An Exploratory Study. *Nonprofit and Voluntary Sector Quarterly*, 46(4), 794-816.

Lee, Z.; Davies, I. (2021), Navigating relative invariance: Perspectives on corporate heritage identity and organizational heritage identity in an evolving nonprofit institution. *Journal of Business Research,* 129, 813-825.

Leenders, M.; Farrell, M.; van der Wurff, R. (2017). Market or society? Dual orientations and the impact on innovativeness in media organizations. *Journal of Strategic Marketing*, 25(5-6), 439-453.

Leijerholt, U. (2021). What about context in internal brand management? Understanding employee brand commitment in the public sector. *Journal of Marketing Management,* 37(13-14), 1243-1266.

Leitch, C.; Harrison, R. (2016). Identity, identity formation and identity work in entrepreneurship: conceptual developments and empirical applications. Entrepreneurship and Regional Development, 28(3-4), 177-190.

Leitch, S.; Davenport, S. (2011). Corporate identity as an enabler and constraint on the pursuit of corporate objectives. *European Journal of Marketing,* 45(44814), 1501-1520.

Lenartowicz, M. (2015). The nature of the university. *Higher Education*, 69(6), 947-961.

Lenberg, P.; Feldt, R.; Tengberg, L. (2019). Misaligned values in software engineering organizations. *Journal of software: Evolution and Process*, 31(3).

Leone, P.; Mantere, S.; Farah, S. (2021). Open theorizing in management and organization studies. *Academy of Management Review,* 46(4), 725-749.

Levin, J.; Damian, A.; Martin, M.; Vazquez, E. (2018). New universities' organizational identities through presidential lenses. Canadian Journal of *Higher Education*, 48(2), 20-38.

Levin, J. (2004). The community college as a baccalaureate-granting institution. *Review of Higher Education*, 28(1), 1-22.

Levine Daniel, J. (2021). All Earned Revenue is Not Created Equal: Revenue Embeddedness as a Framework for Exploring Crowding-In/Crowding-Out Effects. *Voluntas,* 32(5), 1027-1041.

Li, J.; Tang, G.; Chen, Y. (2012). Firms' human resource in information system and sustainable performance: does their organizational identity matter?. *International Journal of Human Resource Management*, 23(18), 3838-3855.

Li, J.; Hambrick, D. (2005). Factional groups: A new vantage on demographic faultlines, conflict, and disintegration in work teams. *Academy of Management Journal*, 48(5), 794-813.

Li, J.; Xin, K.; Pillutla, M. (2002). Multi-cultural leadership teams and organizational identification in international joint ventures. *International Journal of Human Resource Management*, 13(2), 320-337.

Li, Y.; Zhang, G.; Yang, X.; Li, J. (2015). The influence of collectivist human resource management practices on team-level identification. *International Journal of Human Resource Management*, 26(14), 1791-1806.

Liang, S.; Lupina-Wegener, A.; Ullrich, J.; van Dick, R. (2022). Change is Our Continuity': Chinese Managers' Construction of Post-Merger Identification After an Acquisition in Europe. *Journal of Change Management,* 22(1), 59-78.

Liang, X.; Xiu, L.; Wu, S.; Zhang, S. (2017). In search of sustainable legitimacy of private firms in China. *Chinese Management Studies*, 11(3), 555-578.

Liao, Y.; Liu, X.; Kwan, H.; Li, J. (2015). Work-Family Effects of Ethical Leadership. *Journal of Business Ethics*, 128(3), 535-545.

Liao, Y.; Lin, B.; Zhuo, H.; Yang, X. (2021). The Power of Unrequited Love: The Parasocial Relationship, Trust, and Organizational Identification Between Middle-Level Managers and CEOs. *Frontiers in Psychology,* 12, 3743.

Libaers, D.; Wang, T. (2012). Foreign-born academic scientists: entrepreneurial academics or academic entrepreneurs?. *R&D Management*, 42(3), 254-272.

Lin, C.; Chen, S. (2011). Understanding Purchase Intention During Product-Harm Crises: Moderating Effects of Perceived Corporate Ability and Corporate Social Responsibility. *Journal of Business Ethics,* 102(3), 455-471.

Lindqvist, K. (2017). Art ventures as hybrid organisations: tensions and conflicts relating to organisational identity. *International Journal of Entrepreneurial Venturing,* 9(3), 242-259.

Liou, R.; Rao-Nicholson, R. (2019). Corporate name change: Investigating South African multinational corporations' postacquisition performance. *Thunderbird International Business Review,* 61(6), 929-941.

Liou, R.; Rao-Nicholson, R. (2021). Multinational enterprises and Sustainable Development Goals: A foreign subsidiary perspective on tackling wicked problems. *Journal of International Business Policy,* 4(1), 136-151.

Liou, R.; Rao-Nicholson, R.; Sarpong, D. (2018). What is in a name? Cross-national distances and subsidiary's corporate visual identity change in emerging-market firms' cross-border acquisitions. *International Marketing Review,* 35(2), 301-319.

Litchfield, R.; Hirst, G.; Van Knippenberg, D. (2021). Professional network identification: searching for stability in transient knowledge work. *Academy of Management Review,* 46(2), 320-340.

Litrico, J.; Besharov, M. (2019). Unpacking Variation in Hybrid Organizational Forms: Changing Models of Social Enterprise Among Nonprofits, 2000-2013. *Journal of Business Ethics,* 159(2), 343-360.

Liu, Y.; Lam, L.; Loi, R. (2014). Examining professionals' identification in the workplace: The roles of organizational prestige, work-unit prestige, and professional status. Asia Pacific *Journal of Management,* 31(3), 789-810.

Liu, Y.; Li, H.; Goncalves, J.; Kostakos, V.; Xiao, B. (2016). Fragmentation or cohesion? Visualizing the process and consequences of information system diversity, 1993-2012. *European Journal of Information Systems*, 25(6), 509-533.

Liu, Z.; Mei, S.; Guo, Y. (2021). Green human resource management, green organization identity and organizational citizenship behavior for the environment: the moderating effect of environmental values. *Chinese Management Studies,* 15(2), 290-304.

Lobo, S. (2018). Best value': when history, identity and economics clash. Proceedings of the Institution of Civil Engineers: Urban Design and Planning, 171(6), 268-274.

Logue, D.; Clegg, S. (2015). Wikileaks and The News of the World: The Political Circuitry of Labeling. *Journal of Management Inquiry*, 24(4), 394-404.

Lopez, D.; Alejandro Jaramillo, D.; Susaeta Arango, D. (2019). Innovation in Corporate Organizational Culture: Diversity, Motivation and Organizational Pressure as Possible Realities. *Revista,* 22(2), 63-85.

Lopez-Fernandez, B.; Perrigot, R. (2018). Using Websites to Recruit Franchisee Candidates. *Journal of Interactive Marketing*, 42, 80-94.

Lounsbury, M.; Gehman, J.; Glynn, M. (2019). Beyond Homo Entrepreneurus: Judgment and the Theory of Cultural Entrepreneurship. *Journal of Management Studies,* 56(6), 1214-1236.

Lovelace, J.; Bundy, J.; Hambrick, D.; Pollock, T. (2018). The shackles of ceo celebrity: sociocognitive and behavioral role constraints on star leaders. *Academy of Management Review*, 43(3), 419-444.

Lowe, M.; George, G.; Alexy, O. (2012). Organizational identity and capability development in internationalization: transference, splicing and enhanced imitation in Tesco's US market entry. *Journal of Economic Geography*, 12(5), 1021-1054.

Lu, J.; Ren, L.; He, Y.; Lin, W.; Streimikis, J. (2019). Linking corporate social responsibility with reputation and brand of the firm. *Amfiteatru Economic,* 21(51), 442-460.

Lupina-Wegener, A.; Schneider, S.; van Dick, R. (2015). The Role of Outgroups in Constructing a Shared Identity: A Longitudinal Study of a Subsidiary Merger in Mexico. *Management International Review*, 55(5), 677-705.

MacDonald, G. (2013). Theorizing university identity development: multiple perspectives and common goals. *Higher Education*, 65(2), 153-166.

Maclean, M.; Harvey, C.; Sillince, J.; Golant, B. (2018). Intertextuality, Rhetorical History and the Uses of the Past in Organizational Transition. *Organization Studies,* 39(12), 1733-1755.

MacLean, T.; Behnam, M. (2010). The dangers of decoupling: the relationship between compliance programs, legitimacy perceptions, and institutionalized misconduct. *Academy of Management Journal*, 53(6), 1499-1520.

MacLean, T.; Webber, S. (2015). Navigating Multiple Identities Across Multiple Boundaries: A Cross-Level Model of Organizational Identification. *Journal of Management Inquiry*, 24(2), 156-173.

MacLean, T.; Litzky, B.; Holderness, D. (2015). When Organizations Don't Walk Their Talk: A Cross-Level Examination of How Decoupling Formal Ethics Programs Affects Organizational Members. *Journal of Business Ethics*, 128(2), 351-368.

Madsen, V. (2016). Constructing Organizational Identity on Internal Social Media: A Case Study of Coworker Communication in Jyske Bank. *International Journal of Business Communication*, 53(2), 200-223.

Magalhaes, R. (2018). Human-centred organization design. Design Journal, 21(2), 227-246.

Maier, C.; Andersen, M. (2017). Strategic internal communication of corporate heritage identity in a hypermodal context. *Corporate Communications*, 22(1), 36-59.

Manuti, A.; Mininni, G. (2013). Narrating organizational change: an applied psycholinguistic perspective on organizational identity. *Text and Talk*, 33(2), 213-232.

Manz, C.; Anand, V.; Joshi, M.; Manz, K. (2008). Emerging paradoxes in executive leadership: A theoretical interpretation of the tensions between corruption and virtuous values. *Leadership Quarterly*, 19(3), 385-392.

Maor, M. (2020). International competition in the academia: The European challenge. *Public Administration,* 98(4), 1044-1055.

Marafioti, El.; Perretti, F. (2006). International competition in the academia: The European challenge. *Journal of Management Inquiry*, 15(3), 318-326.

Marais, M.; Reynaud, E.; Vilanova, L. (2020). CSR Dynamics in the Midst of Competing Injunctions: The case of Danone. *European Management Review,* 17(1), 19-39.

Mariconda, S.; Zamparini, A.; Lurati, F. (2019). Identity matters How the relevance of a crisis to organizational and stakeholder identities influences reputation damage. *Corporate Communications,* 24(1), 115-127.

Marin, L.; Ruiz, S.; Rubio, A. (2009). The Role of Identity Salience in the Effects of Corporate Social Responsibility on Consumer Behavior. *Journal of Business Ethics*, 84(1), 65-78.

Martin, F. (2022). Organizational Virtues and Organizational Anthropomorphism. *Journal of Business Ethics,* 177(1), 1-17.

Martin, G.; Siebert, S.; Robson, I. (2018). Conformist innovation: an institutional logics perspective on how HR executives construct business school reputations. *International Journal of Human Resource Management*, 29(13), 2027-2053.

Martin, K.; Johnson, J. (2008). A framework for ethical conformity in marketing. *Journal of Business Ethics*, 80(1), 103-109.

Martin K.; Phillips, R. (2022). Stakeholder Friction. *Journal of Business Ethics,* 177(3), 519-531.

Martins, L. (2005). A model of the effects of reputational rankings on organizational change. *Organization Science*, 16(6), 701-720.

Massa, F.; Helms, W.; Voronov, M.; Wang, L. (2017). Emotions uncorked: inspiring evangelism for the emerging practice of cool-climate winemaking in ontario. *Academy of Management Journal*, 60(2), 461-499.

Matherne, C.; Ring, J.; Farmer, S. (2018). Organizational Moral Identity Centrality: Relationships with Citizenship Behaviors and Unethical Prosocial Behaviors. *Journal of Business and Psychology*, 33(6), 711-726.

Matherne, C.; Waterwall, B.; Ring, J.; Credo, K. (2017). Beyond organizational identification: The legitimization and robustness of family identification in the family firm. *Journal of Family Business Strategy*, 8(3), 170-184.

Mathison, K. (2015). Effects of the performance management context on Australian academics' engagement with the scholarship of teaching and learning: a pilot study. *Australian Educational Researcher*, 42(1), 97-116.

Mattarelli, E.; Tagliaventi, M. (2015). How Offshore Professionals' Job Dissatisfaction Can Promote Further Offshoring: Organizational Outcomes of Job Crafting. *Journal of Management Studies*, 52(5), 585-620.

Mattarelli, E.; Tagliaventi, M. (2010). Work-Related Identities, Virtual Work Acceptance and the Development of Glocalized Work Practices in Globally Distributed Teams. *Industry and Innovation*, 17(4), 415-443.

Mattarelli, E.; Tagliaventi, M.; Carli, G.; Gupta, A. (2017). The Role of Brokers and Social Identities in the Development of Capabilities in Global Virtual Teams. *Journal of International Management*, 23(4), 382-398.

Maynard, L.; Adams, A.; Jacobson, S.; Monroe, M. (2021). Evaluating Organizational Identity of Zoos to Enhance Conservation. *Curator,* 64(3), 549-565.

Maynard, L.; Jacobson, S.; Monroe, M.; Savage, A. (2020). Mission impossible or mission accomplished: Do zoo organizational missions influence conservation practices?. *Zoo Biology,* 39(5), 304-314.

Mazutis, D.; Slawinski, N. (2015). Reconnecting Business and Society: Perceptions of Authenticity in Corporate Social Responsibility. *Journal of Business Ethics*, 131(1), 137-150.

Mazzei, M.; Ketchen, D.; Shook, C. (2017). Understanding strategic entrepreneurship: a theoretical toolbox approach. *International Entrepreneurship and Management Journal*, 13(2), 631-663.

McAdam, M.; Brophy, M.; Harrison, R. (2021). Anointed or appointed? Father-daughter succession within the family business. *International Small Business Journal*, 39(6), 576-600.

McClerking, H.; McDaniel, E. (2005). Belonging and doing: Political churches and black political participation. *Political Psychology,* 26(5), 721-733.

McKenny, A.; Short, J.; Zachary, M.; Payne, G. (2012). Assessing Espoused Goals in Private Family Firms Using Content Analysis. *Family Business Review*, 25(3), 298-317.

McLaren, P.; Mills, A. (2013). Internal Cohesion in Response to Institutional Plurality: The Administrative Sciences Association of Canada. *Canadian Journal of Administrative Sciences,* 30(1), 40-55.

McLeod, M.; Moore, C.; Payne, G.; Sexton, J.; Evert, R. (2018). Organizational Virtue and Stakeholder Interdependence: An Empirical Examination of Financial Intermediaries and IPO Firms. *Journal of Business Ethics*, 149(4), 785-798.

McMillan, C. (2016). Old wine in new bottles: docility, attention scarcity and knowledge management. *Journal of Knowledge Management*, 20(6), 1353-1372.

McNamara, G.; Deephouse, D.; Luce, R. (2003). Competitive positioning within and across a strategic group structure: The performance of core, secondary and solitary firms. *Strategic Management Journal,* 24(2), 161-181.

Medina-Craven, M.; Cooper, D.; Penney, C.; Caldas, M. (2021). Family firm employees: identification, stewardship practices and citizenship behaviors. *Journal of Family Business Management,* 11(4), 538-554.

Mejia, C. (2019). Influencing green technology use behavior in the hospitality industry and the role of the green champion. *Journal of Hospitality Marketing and Management,* 28(5), 538-557.

Melewar, T.; Foroudi, P.; Gupta, S.; Kitchen, P.; Foroudi, M. (2017). Integrating identity, strategy and communications for trust, loyalty and commitment. *European Journal of Marketing*, 51(3), 572-604.

Men, L.; Tsai, W. (2014). Perceptual, Attitudinal, and Behavioral Outcomes of Organization-Public Engagement on Corporate Social Networking Sites. *Journal of Public Relations Research,* 26(5), 417-435.

Micelotta, E.; Raynard, M. (2011). Concealing or Revealing the Family? Corporate Brand Identity Strategies in Family Firms. *Family Business Review*, 24(3), 197-216.

Michailova, S.; Piekkari, R.; Storgaard, M.; Tienari, J. (2017). Rethinking Ethnocentrism in International Business Research. *Global Strategy Journal*, 7(4), 335-353.

Michel, S.; Ben-Slimane, K. (2021). The Interplay between Internal and External Identity Work when Institutional Change Threatens the Collective Identity: The Case of a Wholesaler Faced with the Rise of Central Purchasing. *Management,* 24(3), 1-18.

Middleton, S. (2009). Reputation Management in the Salvation Army A Narrative Study. *Journal of Management Inquiry*, 18(2), 145-157.

Miller, G. (2019). Choose Your Friends Wisely: How Organizational Identity Influences Behavior at US Colleges and Universities. *Review of Higher Education,* 42(3), 1185-1206.

Miller, G. (2020). I'll Know One When I See It: Using Social Network Analysis to Define Comprehensive Institutions Through Organizational Identity. *Research in Higher Education,* 61(1), 51-87.

Mills, M.; Bettis, P.; Miller, J.; Nolan, R. (2005). Experiences of academic unit reorganization: Organizational identity and identification in organizational change. *Review of Higher Education*, 28(4), 597-619.

Millward, L.; Haslam, S. (2013). Who are we made to think we are? Contextual variation in organizational, workgroup and career foci of identification. Journal of Occupational and Organizational Psychology, 86(1), 50-66.

Min, N.; Shen, R.; Berlan, D.; Lee, K. (2020). How Organizational Identity Affects Hospital Performance: Comparing Predictive Power of Mission Statements and Sector Affiliation. *Public Performance & Management Review,* 43(4), 845-870.

Miner, K.; Costa, P.; He, Y.; Wooderson, R. (2021). Your Politics Are Making me Sick! Political Identity-Based Workplace Incivility and Physical Health Complaints during Two US Presidential Elections. *Occupational Health Science,* 5(3), 361-389.

Mitchell, R.; Agle, B.; Chrisman, J.; Spence, L. (2011). Toward a Theory of Stakeholder Salience in Family Firms. *Business Ethics Quarterly*, 21(2), 235-255.

Mittal, S.; Dhar, R. (2016). Effect of green transformational leadership on green creativity: A study of tourist hotels. Tourism Management, 57, 118-127.

Mizrahi-Shtelman, R. (2021). Looking for their voice: the formation of identity in the new Israeli principals' organization. *Globalisation, Societies and Education,* 19(1), 81-97.

Mizrahi-Shtelman, R.; Drori, G. (2021). World-Rank and/or Locally Relevant? Organizational Identity in the Mission Statements of Higher Education Organizations in Israel, 2008-2018. *Minerva,* 59(1), 1-25.

Moccia, S.; Zhao, S.; Flanagan, P. (2019). Innovation, dynamic capabilities, leadership, and action plan. *Journal of Enterprising Communities,* 14(1), 113-127.

Moghadam, A.; Tehrani, M. (2011). Predicting model of organizational identity toward its effect on organizational citizenship behaviors (OCBs). *African Journal of Business Management,* 5(23), 9877-9888.

Mohamad, B.; Ismail, A.; Bidin, R. (2017). Corporate Identity Management and Employee Brand Support: Enhancing Marketisation in Higher Education Sector. *Jurnal Komunikasi: Malaysian Journal of Communication*, 33(3), 178-195.

Mohammed, I.; Guillet, B.; Law, R. (2014). Competitor set identification in the hotel industry: A case study of a full-service hotel in Hong Kong. *International Journal of Hospitality Management,* 39, 29-40.

Mohammed, I.; Guillet, B.; Schuckert, M.; Law, R. (2016). An Empirical Investigation of Corporate Identity Communication on Hong Kong Hotels' Websites. *Journal of Hospitality Marketing and Management*, 25(6), 676-705.

Moldavanova, A.; Wright, N. (2020). How Nonprofit Arts Organizations Sustain Communities: Examining the Relationship Between Organizational Strategy and Engagement in Community Sustainability. *American Review of Public Administration,* 50(3), 244-259.

Molenaers, N.; Dewachter, S.; Dellepiane, S. (2011). Moving into the new aid approach, dilemmas for ngos: the belgian case. *Public Administration and Development*, 31(3), 188-204.

Monti, A.; Salvemini, S. (2014). The Barolo brothers Organizational identity and social relationships as strategic decision-making drivers. *Management Decision,* 52(9), 1750-1781.

Moosmayer, D. (2011). Professors as value agents: a typology of management academics' value structures. *Higher Education,* 62(1), 49-67.

Moosmayer, D. (2012). A Model of Management Academics' Intentions to Influence Values. *Academy of Management Learning and Education,* 11(2), 155-173.

Morgan, S. (2011). Are you being outsourced?. *Psychologist,* 24(3), 182-185.

Morris, M.; Neumeyer, X.; Jang, Y.; Kuratko, D. (2018). Distinguishing Types of Entrepreneurial Ventures: AnIdentity-Based Perspective. *Journal of Small Business Management*, 56(3), 453-474.

Morrisette, S.; Oberman, W.; Hunt, I. (2021). Comparative suitability for promotion: ethical transgressions, culture and a third dimension of appraisal. *Management Research Review,* 44(10), 1345-1365.

Morsing, M.; Roepstorff, A. (2015). CSR as Corporate Political Activity: Observations on IKEA's CSR Identity-Image Dynamics. *Journal of Business Ethics*, 128(2), 395-409.

Moss, T.; Short, J.; Payne, G.; Lumpkin, G. (2011). Dual Identities in Social Ventures: An Exploratory Study. *Entrepreneurship: Theory and Practice,* 35(4), 805-830.

Moufahim, M.; Reedy, P.; Humphreys, M. (2015). The Vlaams Belang: The Rhetoric of Organizational Identity. *Organization Studies,* 36(1), 91-111.

Mozahem, N. (2018). Category dynamics and cluster spanning during the emergence of the Lebanese newspaper industry (1851-1879). *Heliyon*, 4(3), e00567.

Mueller, M. (2013). Lack and jouissance in hegemonic discourse of identification with the state. *Organization,* 20(2), 279-298.

Mujib, H. (2017). Organizational Identity: An Ambiguous Concept in Practical Terms. *Administrative Sciences,* 7(3), 28.

Mullens, N. (2021). Beyond the Binary: Troubling Blackness through an Exploration of Institutional and Individual Identity Construction in Ghanaian Higher Education. *Educational Studies- Aeasa,* 57(5), 496-508.

Murphy, C.; Kreiner, G. (2020). Occupational boundary play: Crafting a sense of identity legitimacy in an emerging occupation. *Journal of Organizational Behavior,* 41(9), 871-894.

Murphy, M.; Perrot, F.; Rivera-Santos, M. (2012). New perspectives on learning and innovation in cross-sector collaborations. *Journal of Business Research*, 65(12), 1700-1709.

Myers, K. (2014). Social Identity Issues for Qualitative and Mixed Methods Scholars-Mentors in a Predominantly Quantitative Environment. *Management Communication Quarterly,* 28(3), 466-473.

Nag, R.; Corley, K.; Gioia, D. (2007). The intersection of organizational identity, knowledge, and practice: Attempting strategic change via knowledge grafting. *Academy of Management Journal*, 50(4), 821-847.

Nambisan, S.; Baron, R. (2010). Different Roles, Different Strokes: Organizing Virtual Customer Environments to Promote Two Types of Customer Contributions. *Organization Science*, 21(2), 554-572.

Nason, R.; Bacq, S.; Gras, D. (2018). A behavioral theory of social performance: social identity and stakeholder expectations. *Academy of Management Review*, 43(2), 259-283.

Navis, C.; Glynn, M. (2011). Legitimate distinctiveness and the entrepreneurial identity: influence on investor judgments of new venture plausibility. *Academy of Management Review*, 36(3), 479-499.

Neckebrouck, J.; Manigart, S.; Meuleman, M. (2017). Attitudes of family firms toward outside investors: the importance of organizational identification. *Venture Capital,* 19(1-2), 29-50.

Neumerski, C.; Cohen, D. (2019). The Heart of the Matter: How Reforms Unsettle Organizational Identity. *Educational Policy,* 33(6), 882-915.

Ni, N.; Qian, C.; Crilly, D. (2014). The stakeholder enterprise: Caring for the community by attending to employees. *Strategic Organization,* 12(1), 38-61.

Nicolaou, N.; Souitaris, V. (2016). Can Perceived Support for Entrepreneurship Keep Great Faculty in the Face of Spinouts?. *Journal of Product Innovation Management,* 33(3), 298-319.

Ninan, J.; Sergeeva, N. (2021). Labyrinth of labels: Narrative constructions of promoters and protesters in megaprojects. *International Journal of Project Management,* 39(5), 496-506.

Nissen, M. (2018). The constitution and the singular identity of the collective: who, 'we'?. *Subjectivity*, 11(4), 357-377.

Nissley, N.; Casey, A. (2002). The politics of the exhibition: Viewing corporate museums through the paradigmatic lens of organizational memory. *British Journal of Management,* 13(S2), S35-S45.

Noh, S.; Tolbert, P. (2019). Organizational identities of US art museums and audience reactions. *Poetics,* 72, 94-107.

Nowacki, C.; Monk, A.; Decoster, B. (2021). Who do sovereign wealth funds say they are? Using structural topic modeling to delineate variegated capitalism in their official reports. *Environment and Planning A,* 53(4), 828-857.

Nunes, F; Anderson, J.; Martins, L.; Wiig, S. (2017). The hybrid identity of micro enterprises Contrasting the perspectives of community pharmacies' owners-managers and employees. *Journal of Small Business and Enterprise Development*, 24(1), 34-53.

Nunes, F.; Martins, L. (2018). Janusian, anomic, agent, and steward: How employees perceive the identity of healthcare organizations. International *Journal of Healthcare Management*, 11(2), 143-153.

Nwagwu, W. (2017). Social networking, identity and sexual behaviour of undergraduate students in Nigerian universities. *Electronic Library,* 35(3), 534-558.

Oakes, L.; Townley, B.; Cooper, D. (1998). Business planning as pedagogy: Language and control in a changing institutional field. *Administrative Science Quarterly,* 43(2), 257-292.

Oberg, C. (2016). What creates a collaboration-level identity?. *Journal of Business Research*, 69(9), 3220-3230.

Oberg, C.; Grundstrom, C.; Jonsson, P. (2011). Acquisitions and network identity change. *European Journal of Marketing*, 45(9-10), 1470-1500.

Ocel, H. (2013). The Relationships of Contextual Performance with Person-Organization Fit, Perceived Organizational Prestige and Organizational Identity Strength: The Mediating Role of Organizational Commitment. *Turk Psikoloji Dergisi,* 28(71), 37-56.

Oelsner, A. (2013). The Institutional Identity of Regional Organizations, Or Mercosur's Identity Crisis. *International Studies Quarterly*, 57(1), 115-127.

Oertel, S.; Thommes, K. (2018). History as a Source of Organizational Identity Creation. *Organization Studies,* 39(12), 1709-1731.

O'Kane, C.; Mangematin, V; Geoghegan, W.; Fitzgerald, C. (2015). University technology transfer offices: The search for identity to build legitimacy. *Research Policy*, 44(2), 421-437.

O'Kane, C. (2018). Technology transfer executives' backwards integration: An examination of interactions between university technology transfer executives and principal investigators. *Technovation*, 76-77, 64-77.

Oliver, D.; Roos, J. (2003). Dealing with the unexpected: Critical incidents in the LEGO Mindstorms team. *Human Relations*, 56(9), 1057-1082.

Oliver, D.; Cole, B. (2019). The interplay of product and process in skunkworks identity work: An inductive model. *Strategic Management Journal,* 40(9), 1491-1514.

Oliver, D.; Roos, J. (2007). Beyond text: Constructing organizational identity multimodally. *British Journal of Management,* 18(4), 342-358.

Oliver, D.; Statler, M.; Roos, J. (2010). A Meta-Ethical Perspective on Organizational Identity. *Journal of Business Ethics*, 94(3), 427-440.

Oliver, D.; Vough, H. (2020). Practicing identity in emergent firms: How practices shape founders' organizational identity claims. *Strategic Organization,* 18(1), 75-105.

Omanwar, S.; Agrawal, R. (2022). Servant leadership, organizational identification and turnover intention: an empirical study in hospitals. *International Journal of Organizational Analysis*, 30(2), 239-258.

Onishi, T. (2019). Venture Philanthropy and Practice Variations: The Interplay of Institutional Logics and Organizational Identities. *Nonprofit and Voluntary Sector Quarterly,* 48(2), 241-265.

Onkila, T.; Makela, M.; Jarvenpaa, M. (2018). Employee Sensemaking on the Importance of Sustainability Reporting in Sustainability Identity Change*. Sustainable Development*, 26(3), 217-228.

Ooi, C. (2002). Persuasive histories - Decentering, recentering and the emotional crafting of the past. *Journal of Organizational Change Management*, 15(6), 606-621.

Oreilly, C.; Chatman, J.; Caldwell, D. (1991). People and organizational culture - a profile comparison approach to assessing person-organization fit. *Academy of Management Journal*, 34(3), 487-516.

Orphan, C.; Broom, S. (2021). Life at the people's universities: organizational identification and commitment among regional comprehensive university faculty members in the USA. *Higher Education,* 82(1), 181-201.

Ortiz, L. (2022). Life at the people's universities: organizational identification and commitment among regional comprehensive university faculty members in the USA. *Journal of Strategy and Management,*15(2), 234-255.

Palmer, T.; Short, J. (2008). Mission Statements in US Colleges of Business: An Empirical Examination of Their Content With Linkages to Configurations and Performance. *Academy of Management Learning and Education*, 7(4), 454-470.

Pan, C.; Jiang, Y.; Wang, M.; Xu, S.; Xu, M.; Dong, Y. (2021). How Can Agricultural Corporate Build Sustainable Competitive Advantage through Green Intellectual Capital? A New Environmental Management Approach to Green Agriculture. *International Journal of Environmental Research and Public Health,* 18(15).

Pant, A.; Ramachandran, J. (2017). Navigating identity duality in multinational subsidiaries: A paradox lens on identity claims at Hindustan Unilever 1959-2015. *Journal of International Business Studies*, 48(6), 664-692.

Parent, M.; Seguin, B. (2008). Toward a model of brand creation for international large-scale sporting events: The impact of leadership, context, and nature of the event. *Journal of Sport Management*, 22(5), 526-549.

Parent, M.; Foreman, P. (2007). Organizational image and identity management in large-scale sporting events. *Journal of Sport Management*, 21(1), 15-40.

Park, J. (2014). After pain comes joy: identity gaps in employees' minds. *Personnel Review*, 43(3), 419-437.

Park, J.; Suzuki, S. (2021). Product Creativity as an Identity Issue: Through the Eyes of New Product Development Team Members. *Frontiers of Psychology,* 12, 646766.

Park, K.; Meglio, O.; Bauer, F.; Tarba, S. (2018). Managing patterns of internationalization, integration, and identity transformation: The post-acquisition metamorphosis of an Arabian Gulf EMNC. *Journal of Business Research*, 93, 122-138.

Partanen, J.; Kauppila, O.; Sepulveda, F.; Gabrielsson, M. (2020). Turning strategic network resources into performance: The mediating role of network identity of small- and medium-sized enterprises. *Strategic Entrepreneurship Journal,* 14(2), 178-197.

Paterson, T.; Welbourne, T. (2020). I am therefore I own: Implications of organization-based identity for employee stock ownership. *Human Resource Management,* 59(2), 175-183.

Patvardhan, S.; Gioia, D.; Hamilton, A. (2015). Weathering a meta-level identity crisis: forging a coherent collective identity for an emerging field. *Academy of Management Journal*, 58(2), 405-435.

Paulus, O. (2010). Museums as Serigraphs or Unique Masterpieces: Do American Art Museums Display Differentiation in Their Mission Statements?. *International Journal of Arts Management,* 13(1), 12-28.

Pedersen, J.; Dobbin, F. (2006). In search of identity and ligitimation - Bridging organizational culture and neoinstitutionalism. *American Behavioral Scientist,* 49(7), 897-907.

Pendse, M.; Ojha, A. (2017). Towards an Understanding of Organizational Identity and Organizational Self: Insights from Indian Psychology. *Journal of Human Values*, 23(1), 52-65.

Peng, G.; Beamish, P. (2019). Subnational FDI Legitimacy and Foreign Subsidiary Survival. *Journal of International Management,* 25(3).

Pepple, D.; Davies, E. (2019). Co-worker social support and organisational identification: does ethnic self-identification matter?. *Journal of Managerial Psychology,* 34(8), 573-586.

Pereira, V.; Malik, A. (2018). Identities in transition: the case of emerging market multinational corporations and its response to glocalisation. *Social Identities*, 24(5), 533-547.

Perez, A.; Rodriguez del Bosque, I. (2012). The Role of CSR in the Corporate Identity of Banking Service Providers. *Journal of Business Ethics,* 108(2), 145-166.

Perkmann, M.; Spicer, A. (2014). How Emerging Organizations Take Form: The Role of Imprinting and Values in Organizational Bricolage. *Organization Science*, 25(6), 1785-1806.

Perra, D..; Sidhu, J.; Volberda, H. (2017). How do established firms produce breakthrough innovations? Managerial Identity-Dissemination Discourse and the Creation of Novel Product-Market Solutions. *Journal of Product Innovation Management,* 34(4), 509-525.

Peteraf, M.; Shanley, M. (1997). Getting to know you: A theory of strategic group identity. *Strategic Management Journal,* 18(S1), 165-186.

Peters, K.; Haslam, S.; Ryan, M.; Fonseca, M. (2013). Working With Subgroup Identities to Build Organizational Identification and Support for Organizational Strategy: A Test of the ASPIRe Model. *Group and Organization Management,* 38(1), 128-144.

Peters, R.; Ricks, J.; Doval, C. (2017). Jesus Centered Leadership and Business Applications: An Alternative Approach: Business and Society Review. *Business and Society Review,* 122(4), 589-612.

Petkova, A.; Rindova, V.; Gupta, A. (2013). No News Is Bad News: Sensegiving Activities, Media Attention, and Venture Capital Funding of New Technology Organizations. *Organization Science*, 24(3), 865-888.

Petriglieri, G.; Petriglieri, J.; Wood, J. (2018). Fast Tracks and Inner Journeys: Crafting Portable Selves for Contemporary Careers. *Administrative Science Quarterly,* 63(3), 479-525.

Petriglieri, J. (2011). Under threat: responses to and the consequences of threats to individuals' identities. *Academy of Management Review*, 36(4), 641-662.

Petriglieri, J. (2015). Co-creating Relationship Repair: Pathways to Reconstructing Destabilized Organizational Identification. *Administrative Science Quarterly,* 60(3), 518-557.

Phillips, D.; Kim, Y. (2009). Why pseudonyms? Deception as Identity Preservation Among Jazz Record Companies, 1920-1929. *Organization Science*, 20(3), 481-499.

Piening, E.; Salge, T.; Antons, D.; Kreiner, G. (2020). Standing together or falling apart? Understanding employees’ responses to organizational identity threats. *Academy of Management Review,* 45(2), 325-351.

Pina e Cunha, M.; Neves, P.; Clegg, S.; Costa, S.; Rego, A. (2019). Paradoxes of organizational change in a merger context. *Qualitative Research in Organizations and Management,* 14(3), 217-240.

Pina e Cunha, M.; Simpson, A.; Clegg, S.; Rego, A. (2019). Speak! Paradoxical Effects of a Managerial Culture of 'Speaking Up'. *British Journal of Management,* 30(4), 829-846.

Pinto, J.; Stacey, P. (2010). What's in a Name? Just the Essence of One's Professional Identity. *Industrial and Organizational Psychology*, 3(3), 277-280.

Pitsakis, K.; Biniari, M.; Kuin, T. (2012). Resisting change: organizational decoupling through an identity construction perspective. *Journal of Organizational Change Management*, 25(6), 835-852.

Platou, R.; Aspelund, A. (2019). Managerial rationales for investing and divesting under uncertainty. *Cogent Business and Management,* 6(1), 646766.

Podnar, K.; Golob, U.; Jancic, Z. (2011). Identification with an organisation as a dual construct. *European Journal of Marketing,* 45(44814), 1399-1415.

Ponting, S. (2020). Organizational identity change: impacts on hotel leadership and employee wellbeing. *Service Industries Journal,* 40(1-2), 6-26.

Ponting, S. (2022). Responding to organizational identity change: ethnographic insights from multinational hotel subsidiaries. *Journal of Hospitality and Tourism Technology,* 13(1), 14-33.

Ponting, S.*;* Dillette, A. (2021). (Dis)connected organizational identity of multinational hotel corporations: Employee insights from a Mexican luxury hotel property. *Tourism and Hospitality Research,* 21(1), 86-98.

Porac, J.; Thomas, H. (1990). Taxonomic mental models in competitor definition. *Academy of Management Review,* 15(2), 224-240.

Prange, C. (2021). Agility as the Discovery of Slowness. *California Management Review,* 63(4), 27-50.

Pratt, M.; Rafaeli, A. (2001). Symbols as a language of organizational relationships. *Research in Organizational Behavior*, 23, 93-132.

Pratt, M.; Rockmann, K.; Kaufmann, J. (2006). Constructing professional identity: The role of work and identity learning cycles in the customization of identity among medical residents. *Academy of Management Journal*, 49(2), 235-262.

Pratt, M.; Lepisto, D.; Dane, E. (2019). The Hidden Side of Trust: Supporting and Sustaining Leaps of Faith among Firefighters. *Administrative Science Quarterly,* 64(2), 398-434.

Purchase, S.; Rosa, R.; Schepis, D. (2016). Identity construction through role and network position. *Industrial Marketing Management*, 54, 154-163.

Puusa, A.; Kuittinen, M.; Kuusela, P. (2013). Paradoxical Change and Construction of Identity in an Educational Organization. Educational Management Administration and *Leadership,* 41(2), 165-178.

Rafaeli, A.; Oliver, A. (1998). Employment ads - A configurational research agenda. *Journal of Management Inquiry*, 7(4), 342-358.

Raffaelli, R.; Glynn, M.; Tushman, M. (2019). Frame flexibility: The role of cognitive and emotional framing in innovation adoption by incumbent firms. *Stragetic Management Journal,* 40(7), 1013-1039.

Rajala, R.; Westerlund, M.; Lampikoski, T. (2016). Environmental sustainability in industrial manufacturing: re-examining the greening of Interface's business model. *Journal of Cleaner Production,* 115, 52-61.

Ran, B.; Duimering, P. (2007). Imaging the organization - Language use in organizational identity claims. *Journal of Business and Technical Communication,* 21(2), 155-187.

Ran, B.; Golden, T. (2011). Who are we? The Social Construction of Organizational Identity Through Sense-Exchanging. *Administration and Society,* 43(4), 417-445.

Randel, A.; Jaussi, K.; Standifird, S. (2009). Organizational Responses to Negative Evaluation by External Stakeholders The Role of Organizational Identity Characteristics in Organizational Response Formulation. *Business and Society,* 48(4), 438-466.

Randerson, K.; Radu-Lefebvre, M. (2021). Managing Ambivalent Emotions in Family Businesses: Governance Mechanisms for the Family, Business, and Ownership Systems. *Entrepreneurship Research Journal,* 11(3), 159-176.

Rao, H.; Davis, G.; Ward, A. (2000). Embeddedness, social identity and mobility: Why firms leave the NASDAQ and join the New York Stock Exchange. *Administrative Science Quarterly,* 45(2), 268-292.

Rao, H.; Dutta, S. (2018). Why Great Strategies Spring from Identity Movements. *Strategy Science,* 3(1), 313-322.

Rasmussen, J. (2017). Welcome to Twitter, @CIA. Better late than never': Communication professionals' views of social media humour and implications for organizational identity. *Discourse and Communication,* 11(1), 89-110.

Rau, S.; Schneider-Siebke, V.; Guenther, C. (2019). Family Firm Values Explaining Family Firm Heterogeneity. *Family Business Review,* 32(2), 195-215.

Ravasi, D.; Lojacono, G. (2005). Managing design and designers for strategic renewal*. Long Range Planning*, 38(1), 51-77.

Ravasi, D.; Phillips, N. (2011). Strategies of alignment: Organizational identity management and strategic change at Bang & Olufsen. *Strategic Organization,* 9(2), 103-135.

Ravasi, D.; Rindova, V.; Stigliani, I. (2019). The stuff of legend: history, memory and the temporality of organizational identity construction. *Academy of Management Journal,* 62(5), 1523-1555.

Ravasi, D.; Schultz, M. (2006). Responding to organizational identity threats: Exploring the role of organizational culture. *Academy of Management Journal*, 49(3), 433-458.

Ravishankar, M.; Pan, S. (2008). The influence of organizational identification on organizational knowledge management (KM). *Omega*, 36(2), 221-234.

Ray, J.; Smith, A. (2012). Using Photographs to Research Organizations: Evidence, Considerations, and Application in a Field Study. *Organizational Research Methods,* 15(2), 288-315.

Reade, C. (2001). Antecedents of organizational identification in multinational corporations: fostering psychological attachment to the local subsidiary and the global organization. *International Journal of Human Resource Management*, 12(8), 1269-1291.

Reay, T. (2009). Family-Business Meta-Identity, Institutional Pressures, and Ability to Respond to Entrepreneurial Opportunities. *Entrepreneurship: Theory and Practice,* 33(6), 1265-1270.

Reed, H. (2017). Corporations as agents of social change: A case study of diversity at Cummins Inc.. *Business History,* 59(6), 821-843.

Reed, J.; Higgins, G. (2017). Measuring complexity: a confirmatory factor analysis approach. *Journal of Contemporary Criminal Justice,* 33(4), 380-391.

Reficco, E.; Gutierrez, R.; Helena Jaen, M.; Auletta, N. (2018). Collaboration mechanisms for sustainable innovation. *Journal of Cleaner Production,* 203, 1170-1186.

Reger, R.; Gustafson, L.; Demarie, S.; Mullane, J. (1994). Reframing the organization - why implementing total quality is easier said than done. *Academy of Management Review*, 19(3), 565-584.

Reid, W.; Karambayya, R. (2009). Impact of dual executive leadership dynamics in creative organizations. *Human Relations*, 62(7), 1073-1112.

Rein, G. (2005). A reference model for designing effective reputation information systems. *Journal of Information Science,* 31(5), 365-380.

Reissner, S. (2010). Change, meaning and identity at the workplace. *Journal of Organizational Change Management*, 23(3), 287-299.

Reissner, S. (2019). We are this hybrid': Members' search for organizational identity in an institutionalized public-private partnership. *Public Administration,* 97(1), 48-63.

Renfree, G.; Kohe, G. (2019). Running the club for love: challenges for identity, accountability and governance relationships. *European Journal for Sport and Society,* 16(3), 210-228.

Reynolds, C. (2017). From transgression to tradition: Relationality, organizational absorption, and the Lascivious Costume Ball, 1970-1984. *Organization,* 24(6), 916-937.

Reynolds, S.; Dang, C. (2017). Are the Customers of Business Ethics Courses Satisfied? An Examination of One Source of Business Ethics Education Legitimacy. *Business and Society,* 56(7), 947-974.

Rho, E.; Yun, T.; Lee, K. (2015). Does organizational image matter? Image, Identification, and Employee Behaviors in Public and Nonprofit Organizations. *Public Administration Review,* 75(3), 421-431.

Riantoputra, C. (2010). Know Thyself: Examining Factors That Influence the Activation of Organizational Identity Concepts in Top Managers' Minds. *Group and Organization Management,* 35(1), 8-38.

Riemenschneider, C.; Armstrong, D. (2021). The development of the perceived distinctiveness antecedent of information systems professional identity. *MIS Quarterly: Management Information Systems*, 45(3), 1149-1186.

Riener, G.; Wiederhold, S. (2016). Team building and hidden costs of control. *Journal of Economic Behavior and Organization,* 123, 1-18.

Riketta, M.; Van Dick, R. (2005). Foci of attachment in organizations: A meta-analytic comparison of the strength and correlates of workgroup versus organizational identification and commitment. *Journal of Vocational Behavior,* 67(3), 490-510.

Ritchie, W.; Young, G.; Shahzad, A.; Kolodinsky, R; Melnyk, S. (2015). The influence of plural organizational forms on beliefs and outcomes related to new product adoption. *Management Decision,* 53(7), 1619-1641.

Rive, J.; Bonnet, M.; Parmentier, C.; Pelazzo-Plat, V.; Pignet-Fall, L. (2017). A contribution to the laying of foundations for dialogue between socially responsible management schools. *International Journal of Management Education,* 15(2), 238-248.

Rodrigues, C.; Krishnamurthy, A. (2016). Mirror, Mirror on the Wall: Identity-Image Interactions for the Sales Force in High Threat Situations. *European Management Review,* 13(4), 307-323.

Rodrigues, S.; Child, J. (2008). The development of corporate identity: A political perspective. *Journal of Management Studies*, 45(5), 885-911.

Rodriguez, C.; Belanger, E. (2014). Stories and metaphors in the sensemaking of multiple primary health care organizational identities. *BMC Family Practice,* 15(1), 1-10.

Rodriguez, C.; Pawlikowska, T.; Schweyer, F.; Lopez-Roig, S.; Belanger, E.; Burns, J.; Huge, S.; Angeles Pastor-Mira, M.; Tellier, P.; Spencer, S.; Fiquet, L.; Pereiro-Berenguer, I. (2014). Family physicians' professional identity formation: a study protocol to explore impression management processes in institutional academic contexts. *BMC Medical Education*, 14(1), 1-11.

Rodriguez, C.; Pozzebon, M. (2010). The implementation evaluation of primary care groups of practice: a focus on organizational identity. *BMC Family Practice*, 11(1), 1-10.

Rogers, K.; Ashforth, B. (2017). Respect in Organizations: Feeling Valued as We and Me. *Journal of Management,* 43(5), 1578-1608.

Romanelli, E.; Khessina, O. (2005). Regional industrial identity: Cluster configurations and economic development. *Organization Science*, 16(4), 344-358.

Romani, L.; Szkudlarek, B. (2014). The Struggles of the Interculturalists: Professional Ethical Identity and Early Stages of Codes of Ethics Development. *Journal of Business Ethics*, 119(2), 173-191.

Rondeaux, G. (2014). What are the dynamics of organizational identification in the course of modernization processesAnalysis of a Belgian administration. *International Review of Administrative Sciences,* 80(1), 110-130.

Rothman, N..; Pratt, M.; Rees, L.; Vogus, T. (2017). Understanding the dual nature of ambivalence: why and when ambivalence leads to good and bad outcomes. *Academy of Management Annals*, 11(1), 33-72.

Rothmann, W.; Koch, J. (2014). Creativity in strategic lock-ins: The newspaper industry and the digital revolution. *Technological Forecasting and Social Change,* 83, 66-83.

Rousseau, M.; Kellermanns, F.; Zellweger, T.; Beck, T. (2018). Relationship Conflict, Family Name Congruence, and Socioemotional Wealth in Family Firms. *Family Business Review*, 31(4), 397-416.

Roussin, C.; Webber, S. (2012). Impact of Organizational Identification and Psychological Safety on Initial Perceptions of Coworker Trustworthiness. *Journal of Business and Psychology*, 27(3), 317-329.

Ruef, M. (2000). The emergence of organizational forms: A community ecology approach. *American Journal of Sociology*, 106(3), 658-714.

Ruef, M. (1999). Social ontology and the dynamics of organizational forms: Creating market actors in the healthcare field, 1966-1994. *Social Forces,* 77(4), 1403-1432.

Ruef, M.; Patterson, K. (2009). Credit and Classification: The Impact of Industry Boundaries in Nineteenth-century America. *Administrative Science Quarterly,* 54(3), 486-520.

Ryan, A.; Ford, J. (2010). Organizational Psychology and the Tipping Point of Professional Identity*. Industrial and Organizational Psychology,* 3(3), 241-258.

Salonen, A.; Jaakkola, E. (2015). Firm boundary decisions in solution business: Examining internal vs. External resource integration. *Industrial Marketing Management*, 51, 171-183.

Sanchez-Chaparro, T.; Angel Soler-Vicen, M.; Gomez-Frias, V. (2022). Be good and look good: Communicating the triple bottom line through corporate websites. *Journal of Business Research,* 144, 136-145.

Sanders, M. (2015). Being Nonprofit-Like in a Market Economy: Understanding the Mission-Market Tension in Nonprofit Organizing. *Nonprofit and Voluntary Sector Quarterly*, 44(2), 205-222.

Saqib, Z. (2019). Construction of organizational identity claim and understanding in nonprofits: Evidence from Pakistan's voluntary organizations. *Nonprofit Management & Leadership,* 30(2), 233-254.

Sarasvuo, S. (2021). Are we one, or are we many? Diversity in organizational identities versus corporate identities. *Journal of Product and Brand Management,* 30(6), 788-805.

Sargolzaee, A.; Moghaddam, F.; Tanideh, H.; Shahreki, M. (2017). An analysis of the relationship between organizational identify and organizational structure in electricity company in sistan and baluchestan province. *Quid: Investigacion Ciencia Y Tecnologia*, (1), 2456-2463.

Sarkar, S.; Osiyevskyy, O.; Clegg, S. (2018). Incumbent capability enhancement in response to radical innovations. *European Management Journal,* 36(3), 353-365.

Sarma, S. (2017). Rhetorical strategies in Indian commercial microfinance. *International Journal of Sociology and Social Policy,* 37(9-10), 572-590.

Sato, I.; Haga, M.; Yamada, M. (2015). Lost and Gained in Translation: The Role of the American Model' in the Institution-Building of a Japanese University Press. Cultural Sociology, 9(3), 347-363.

Savani, K.; Zou, X. (2019). Making the Leader Identity Salient Can Be Demotivating. *Journal of Experimental Psychology: Applied,* 25(2), 245-255.

Savas, S. (2016). Factors affecting donations in U.S. Retail stores: A conceptual framework. Journal of Retailing and Consumer Services, 33, 178-185.

Scarlata, M.; Alemany, L. (2010). Deal Structuring in Philanthropic Venture Capital Investments: Financing Instrument,Valuation and Covenants. *Journal of Business Ethics*, 95(2), 121-145.

Schad, J.; Lewis, M..; Raisch, S.; Smith, W. (2016). Paradox Research in Management Science: Looking Back to Move Forward. *Academy of Management Annals*, 10(1), 5-64.

Schauerte, R.; Feiereisen, S.; Malter, A. (2021). What does it take to survive in a digital world? Resource-based theory and strategic change in the TV industry. *Journal of Cultural Economics,* 45(2), 263-293.

Scheitle, C. (2009). Identity and Government Funding in Christian Nonprofits. Social Science Quarterly, 90(4), 816-833.

Scherer, S. (2017). Organizational Identity and Philanthropic Institutions Patterns of Strategy, Structure, and Grantmaking Practices. *Nonprofit Management & Leadership,* 28(1), 105-123.

Schilke, O. (2018). A micro-institutional inquiry into resistance to environmental pressures. *Academy of Management Journal*, 61(4), 1431-1466.

Schmid, H. (2013). Nonprofit Human Services: Between Identity Blurring and Adaptation to Changing Environments. *Administration in Social Work*, 37(3), 242-256.

Schmidt, G.; Landers, R. (2010). Strengthening Shared Identity in I-O Psychology Through Online Social Networks. *Industrial and Organizational Psychology,* 3(3), 286-288.

Schneiker, A. (2019). Telling the Story of the Superhero and the Anti-Politician as President: Donald Trump's Branding on Twitter. *Political Studies Review,* 17(3), 210-223.

Schreiter, K.; Ravasi, D. (2018). Institutional Pressures and Organizational Identity: The Case of Deutsche Werkstatten Hellerau in the GDR and Beyond, 1945-1996. *Business History Review,* 92(3), 453-481.

Schultz, M.; Hernes, T. (2013). A Temporal Perspective on Organizational Identity. *Organization Science*, 24(1), 1-21.

Schultz, M.; Hernes, T. (2020). Temporal interplay between strategy and identity: Punctuated, subsumed, and sustained modes. *Strategic Organization,* 18(1), 106-135.

Schultz, M.; Hinings, B. (2012). A Comment at the Border Between Institutional and Organizational Culture Theories. *Journal of Management Inquiry*, 21(1), 107-108.

Schwarz, G.; Watson, B. (2005). The influence of perceptions of social identity on information technology-enabled change. *Group and Organization Management,* 30(3), 289-318.

Scott, S. (1997). Social identification effects in product and process development teams. *Journal of Engineering and Technology Management,* 14(2), 97-127.

Seeber, M.; Barberio, V.; Huisman, J.; Mampaey, J. (2019). Factors affecting the content of universities' mission statements: an analysis of the United Kingdom higher education system. *Studies in Higher Education,* 44(2), 230-244.

Seeber, M.; Lepori, B.; Montauti, M.; Enders, J.; de Boer, H.; Weyer, E.; Bleiklie, I.; Hope, K.; Michelsen, S.; Mathisen, G.; Frolich, N.; Scordato, L.; Stensaker, B.; Waagene, E.; Dragsic, Z.; Kretek, P.; Kruecken, G.; Magalhaes, A.; Ribeiro, F.; Sousa, S.; Veiga, A.; Santiago, R.; Marini, G.; Reale, E. (2015). European universities as complete organizations? Understanding identity, hierarchy and rationality in public organizations. *Public Management Review,* 17(10), 1444-1474.

Seiler, B.; Bortnowska, H. (2019). The identity of the largest enterprises located in Poland and communication of corporate social activities on corporate websites. *Management-Poland,* 23(2), 98-123.

Seong, S.; Godart, F. (2018). Influencing the influencers: diversification, semantic strategies, and creativity evaluations. *Academy of Management Journal*, 61(3), 966-993.

Sgourev, S. (2021). Materiality as a Basis for Valuation Entrepreneurship: Re-modeling Impressionism. *Organization Science,* 32(5), 1235-1255.

Sha, B. (2009). Exploring the Connection Between Organizational Identity and Public Relations Behaviors: How Symmetry Trumps Conservation in Engendering Organizational Identification. *Journal of Public Relations Research,* 21(3), 295-317.

Shamir, B; Zakay, E; Brainin, E; Popper, M (2000). Leadership and social identification in military units: Direct and indirect relationships. *Journal of Applied Social Psychology,* 30(3), 612-640.

Shapiro, D.; Hom, P.; Shen, W.; Agarwal, R. (2016). How do leader departures affect subordinates' organizational attachment? A 360-degree relational perspective. *Academy of Management Review*, 41(3), 479-502.

Sharma, S. (2000). Managerial interpretations and organizational context as predictors of corporate choice of environmental strategy. *Academy of Management Journal*, 43(4), 681-697.

Sharp, Z. (2018). Existential Angst and Identity Rethink: The Complexities of Competition for the Nonprofit. *Nonprofit and Voluntary Sector Quarterly*, 47(4), 767-788.

Shen, H.; Kim, J. (2012). The Authentic Enterprise: Another Buzz Word, or a True Driver of Quality Relationships?. *Journal of Public Relations Research,* 24(4), 371-389.

Shepherd, D.; Sutcliffe, K. (2015). The Use of Anthropomorphizing as a Tool for Generating Organizational Theories. *Academy of Management Annals*, 9(1), 97-142.

Shi, L.; Miles, A. (2020). Non-effectual, non-customer effectual, or customer-effectual: A conceptual exploration of the applicability of the effectuation logic in startup brand identity construction. *Journal of Business Research,* 113, 168-179.

Shih, C. (1988). National role conception as foreign-policy motivation - the psychocultural bases of chinese diplomacy. *Political Psychology,* 9(4), 599-631.

Shipilov, A.; Gulati, R.; Kilduff, M.; Li, S.; Tsai, W. (2014). Relational pluralism within and between organizations. *Academy of Management Journal*, 57(2), 449-459.

Sidorova, A.; Evangelopoulos, N.; Valacich, J.; Ramakrishnan, T. (2008). Uncovering the intellectual core of the information systems discipline. *MIS Quarterly: Management Information Systems,* 32(3), 467-482.

Sikavica, K.; Perrault, E.; Rehbein, K. (2020). Who Do They Think They Are? Identity as an Antecedent of Social Activism by Institutional Shareholders. *Business and Society,* 59(6), 1228-1268.

Sillince, J.; Golant, B. (2018). Making connections: A process model of organizational identification. *Human Relations*, 71(3), 349-374.

Sitaloppi, J.; Rajala, R.; Hietala, H. (2021). Integrating CSR with Business Strategy: A Tension Management Perspective. *Journal of Business Ethics,* 174(3), 507-527.

Simoes, C.; Sebastiani, R. (2017). The Nature of the Relationship Between Corporate Identity and Corporate Sustainability: Evidence from The Retail Industry. *Business Ethics Quarterly*, 27(3), 423-453.

Skille, E.; Syversen, T.; Hanstad, D. (2020). A one-off event and the construction of organisational identity: the case of the 2016 Lillehammer Youth Olympic Games Committee. *European Journal for Sport and Society,* 17(1), 11-25.

Skilton, P.; Purdy, J. (2017). Authenticity, Power, and Pluralism: A Framework for Understanding Stakeholder Evaluations of Corporate Social Responsibility Activities. *Business Ethics Quarterly*, 27(1), 99-123.

Skippari, M.; Laukkanen, M.; Salo, J. (2017). Cognitive barriers to collaborative innovation generation in supply chain relationships. *Industrial Marketing Management*, 62, 108-117.

Slavich, B.; Cappetta, R.; Giangreco, A. (2014). Exploring the link between human resource practices and turnover in multi-brand companies: The role of brand units' images. *European Management Journal,* 32(2), 177-189.

Smerek, R. (2013). Sensemaking and New College Presidents: A Conceptual Study of the Transition Process. *Review Of Higher Education*, 36(3), 371-403.

Smith, E. (2011). Identities as Lenses: How Organizational Identity Affects Audiences' Evaluation of Organizational Performance. *Administrative Science Quarterly,* 56(1), 61-94.

Smith, W.; Besharov, M. (2019). Bowing before Dual Gods: How Structured Flexibility Sustains Organizational Hybridity*. *Administrative Science Quarterly,* 64(1), 1-44.

Smith, W.; Besharov, M.; Wessels, A.; Chertok, M. (2012). A Paradoxical Leadership Model for Social Entrepreneurs: Challenges, Leadership Skills, and Pedagogical Tools for Managing Social and Commercial Demands. *Academy of Management Learning and Education*, 11(3), 463-478.

Smith, W.; Gonin, M.; Besharov, M. (2013). Managing Social-Business Tensions: A Review and Research Agenda for Social Enterprise. *Business Ethics Quarterly*, 23(3), 407-442.

Smith, W.; Lewis, M. (2011). Toward a theory of paradox: a dynamic equilibrium model of organizing. *Academy of Management Review*, 36(2), 381-403.

Snihur, Y. (2016). Developing optimal distinctiveness: organizational identity processes in new ventures engaged in business model innovation. *Entrepreneurship and Regional Development*, 28(3-4), 259-285.

Snihur, Y. (2018). Responding to business model innovation: organizational unlearning and firm failure. *Learning Organization,* 25(3), 190-198.

Snihur, Y. (2022). Sowing the seeds of failure: Organizational identity dynamics in new venture pivoting. *Journal of Business Venturing,* 37(1).

Soewamo, N.; Tjahjadi, B.; Fithrianti, F. (2019). Green innovation strategy and green innovation The roles of green organizational identity and environmental organizational legitimacy. *Management Decision,* 57(11), 3061-3078.

Solebello, N.; Tschirhart, M.; Leiter, J. (2016). The paradox of inclusion and exclusion in membership associations. *Human Relations*, 69(2), 439-460.

Sonenshein, S. (2005). Business ethics and internal social criticism. *Business Ethics Quarterly*, 15(3), 475-498.

Sonenshein, S.; Nault, K.; Obodaru, O. (2017). Competition of a Different Flavor: How a Strategic Group Identity Shapes Competition and Cooperation. *Administrative Science Quarterly,* 62(4), 626-656.

Song, W.; Ren, S.; Yu, J. (2019). Bridging the gap between corporate social responsibility and new green product success: The role of green organizational identity. *Business Strategy and the Environment,* 28(1), 88-97.

Song, W.; Yu, H. (2018). Green Innovation Strategy and Green Innovation: The Roles of Green Creativity and Green Organizational Identity. *Corporate Social Responsibility and Environmental Management,* 25(2), 135-150.

Sorensen, A.; Jordan, R. (2016). Impacts and Implications of Researcher Identity and Academic Practice: Future Directions for Public Engagement and Ecological Research. *Human Ecology,* 44(3), 375-391.

Sorour, M.; Boadu, M.; Soobaroyen, T. (2021). The role of Corporate Social Responsibility in Organisational Identity Communication, Co-Creation and Orientation. *Journal of Business Ethics,* 173(1), 89-108.

Sosin, M. (2012). Social Expectations, Constraints, and Their Effect on Nonprofit Strategies. *Nonprofit and Voluntary Sector Quarterly*, 41(6), 1231-1250.

Soulsby, A. (2022). Foreign direct investment and the undertow of history: Nationhood and the influence of history on the Czech-German relationship. *Business History,* 64(4), 727-754.

Spillane, J.; Seelig, J.; Blaushild, N.; Cohen, D.; Peurach, D. (2019). Educational System Building in a Changing Educational Sector: Environment, Organization, and the Technical Core. *Educational Policy,* 33(6), 846-881.

Stanczyk, S. (2019). Business ecosystem identity construct. *Transformations in Business and Economics,* 18(2B), 674-693.

Stanske, S.-; Rauch, M.; Canato, A. (2020). Anti-identity strategizing: The dynamic interplay of who we are and who we are not. *Strategic Organization,* 18(1), 136-170.

Steffens, N.; Haslam, S.; Reicher, S.; Platow, M.; Fransen, K.; Yang, J.; Ryan, M.; Jetten, J.; Peters, K.; Boen, F. (2014). Leadership as social identity management: Introducing the Identity Leadership Inventory (ILI) to assess and validate a four-dimensional model. *Leadership Quarterly*, 25(5), 1001-1024.

Steger, T. (2007). The stories metaphors tell: Metaphors as a tool to decipher tacit aspects in narratives. Field Methods, 19(1), 3-23.

Stein, M. (2015). Double Trouble: Sibling Rivalry and Twin Organizations in the 2008 Credit Crisis. *British Journal of Management,* 26(2), 182-196.

Stein, M.; Pinto, J. (2011). The Dark Side of Groups: A Gang at Work in Enron. *Group and Organization Management,* 36(6), 692-721.

Steiner, L.; Sundstrom, A.; Sammalisto, K. (2013). An analytical model for university identity and reputation strategy work. *Higher Education*, 65(4), 401-415.

Stenberg, A. (2007). Green ideas travelling across organizational boundaries. Building Research and Information, 35(5), 501-513.

Stenling, C. (2013). The Introduction of Drive-in Sport in Community Sport Organizations As an Example of Organizational Non-Change. *Journal of Sport Management*, 27(6), 497-509.

Stensaker, B. (2015). Organizational identity as a concept for understanding university dynamics. *Higher Education*, 69(1), 103-115.

Stensaker, B.; Fumasoli, T. (2017). Multi-level strategies in universities: Coordination, contestation or creolisation ?. *Higher Education Quarterly,* 71(3), 263-273.

Stevens, R.; Moray, N.; Bruneel, J. (2015). The Social and Economic Mission of Social Enterprises: Dimensions, Measurement, Validation, and Relation. *Entrepreneurship: Theory and Practice,* 39(5), 1051-1082.

Stevens, R.; Moray, N.; Bruneel, J.; Clarysse, B. (2015). Attention allocation to multiple goals: The case of for-profit social enterprises. *Strategic Management Journal,* 36(7), 1006-1016.

Stiehl, E.; Forst, L. (2018). Safety Climate Among Nontraditional Workers in Construction: Arguing for a Focus on Construed External Safety Image. *New Solutions-A Journal of Environmental and Occupational Health Policy*, 28(1), 33-54.

Stiles, D. (2011). Disorganization, disidentification and ideological fragmentation: Verbal and pictorial evidence from a British business school. *Culture and Organization,* 17(1), 5-30.

Stinchcomb, J.; Applegate, B.; Kerle, K.; Stojkovic, S. (2012). Moving Toward Utopia: Visions of Progress for American Jails. *Journal of Contemporary Criminal Justice,* 28(1), 23-41.

Storgaard, M.; Tienari, J.; Piekkari, R.; Michailova, S. (2020). Holding On While Letting Go: Neocolonialism as Organizational Identity Work in a Multinational Corporation. *Organization Studies,* 41(11), 1469-1489.

Summerville, T. (1998). Understanding the current political climate in Quebec (After the 1998 election). *Dalhousie Review*, 78(1), 119-139.

Sveningsson, S.; Larsson, M. (2006). Fantasies of Leadership: Identity Work. *Leadership,* 2(2), 203-224.

Tan, B.; Pan, S.; Chen, W.; Huang, L. (2020). Organizational sensemaking in ERP implementation: the influence of sensemaking structure. *MIS Quarterly: Management Information Systems,* 44(4), 1773-1809.

Tang, C.; Naumann, S. (2016). The impact of three kinds of identity on research and development employees' incremental and radical creativity. *Thinking Skills and Creativity,* 21, 123-131.

Tasdan, M. (2015). Elementary School Teachers' Perception of Organizational Identification. *Egitim ve Bilim-Education and Science,* 40(180), 327-342.

Tasselli, S.; Kilduff, M.; Landis, B. (2018). Personality change: implications for organizational behavior. *Academy of Management Annals*, 12(2), 467-493.

Tata, J.; Prasad, S. (2015). National cultural values, sustainability beliefs, and organizational initiatives. *International Journal of Cross Cultural Management,* 22(2), 278-296.

Temouri, Y.; Pereira, V.; Jones, C.; Malik, A.; Rowley, C. (2022). Towards a new corporate responsibility and governance? Tax haven and other identity characteristics of Asia-Pacific multinational coroporations. *Asia Pacific Business Review,* 28(2), 157-164.

Teram, E. (2010). Organizational Change Within Morally Ambiguous Contexts: A Case Study of Conflicting Postmerger Discourses. *Journal of Applied Behavioral Science*, 46(1), 38-54.

Thiele, F.; Wendt, M. (2017). Family firm identity and capital structure decisions. *Journal of Family Business Management*, 7(2), 221-239.

Thoger Christensen, L.; Askegaard, S. (2001). Corporate identity and corporate image revisited - A semiotic perspective. *European Journal of Marketing,* 35(44624), 292-315.

Thomas, L.; Ritala, P. (2022). Ecosystem Legitimacy Emergence: A Collective Action View. *Journal of Management,* 48(3), 515-541.

Tormala, M.; Gyrd-Jones, R. (2017). Development of new B2B venture corporate brand identity: A narrative performance approach. *Industrial Marketing Management*, 65, 76-85.

Touati, N.; Rodriguezm C.; Paquette, M.; Maillet, L.; Denis, J. (2019). Professional Role Identity: At the Heart of Medical Collaboration Across Organisational Boundaries. *International Journal of Integrated Care,* 19(2).

Toubiana, M. (2020). Once in orange always in orange? Identity paralysis and the enduring influence of institiutional logics on identity. *Academy of Management Journal,* 63(6), 1739-1774.

Townley, B. (2002). The role of competing rationalities in institutional change. *Academy of Management Journal*, 45(1), 163-179.

Tracey, P.; Phillips, N. (2016). Managing the consequences of organizational stigmatization: identity work in a social enterprise. *Academy of Management Journal*, 59(3), 740-765.

Tracy, S.; Myers, K.; Scott, C. (2006). Cracking jokes and crafting selves: Sensemaking and identity management among human service workers. *Communication Monographs*, 73(3), 283-308.

Tran, Q., Nguyen, T.; Ho, D.; Duong, D. (2021). The Impact of Corporate Social Responsibility on Employee Management: A Case Study in Vietnam. *Journal of Asian Finance Economics and Business,* 8(4), 1033-1045.

Trevino, L.; Weaver, G.; Brown, M. (2008). It's lovely at the top: Hierarchical levels, identities, and perceptions of organizational ethics. *Business Ethics Quarterly*, 18(2), 233-252.

Tsai, Y.; Joe, S.; Lin, C.; Chiu, C.; Shen, K. (2015). Exploring corporate citizenship and purchase intention: mediating effects of brand trust and corporate identification*. Business Ethics,* 24(4), 361-377.

Tsai, Y.; Lin, C.; Ma, H.; Wang, R. (2015). Modeling corporate social performance and job pursuit intention: Forecasting the job change of professionals in technology industry. *Technological Forecasting and Social Change,* 99, 14-21.

Tsukamoto, N.; Hirata, A.; Funaki, Y. (2019). Organizational identity and the state of organizational identification in nursing organizations. *Nursing Open,* 6(4), 1571-1579.

Tuan, L. T. (2018). Activating tourists' citizenship behavior for the environment: the roles of CSR and frontline employees' citizenship behavior for the environment. *Journal of Sustainable Tourism*, 26(7), 1178-1203.

Tuohy, A.; Wrennall, M.; McQueen, R.; Stradling, S. (1993). Effect of socialization factors on decisions to prosecute - the organizational adaptation of scottish police recruits. Law and Human Behavior, 17(2), 167-181.

Tyworth, M. (2014). Organizational identity and information systems: how organizational ICT reflect who an organization is. *European Journal of Information Systems*, 23(1), 69-83.

Umphress, E.; Tihanyi, L.; Bierman, L.; Gogus, C. (2013). Personal lives? The effects of nonwork behaviors on organizational image. Organizational Psychology Review, 3(3), 199-221.

Ungureanu, P.; Bertolotti, F.; Mattarelli, E.; Bellesia, F. (2020). Collaboration and identity formation in strategic interorganizational partnerships: An exploration of swift identity processes. *Strategic Organization,* 18(1), 171-211.

Uribe, J.; Sytch, M.; Kim, Y. (2020). When Friends Become Foes: Collaboration as a Catalyst for Conflict. *Administrative Science Quarterly,* 65(3), 751-794.

Vaast, E.; Levina, N. (2015). Speaking as one, but not speaking up: Dealing with new moral taint in an occupational online community. Information and *Organization,* 25(2), 73-98.

Valsecchi, R.; Anderson, N.; Balta, M.; Harrison, J. (2019). The Creation of a Hybrid and Innovative Model of Occupational Health Delivery through the Lens of Institutional Work. *European Management Review,* 16(4), 975-996.

van der Stoep, J.; Sleebos, E.; van Knippenberg, D.; van de Bunt, G. (2020). The empowering potential of intergroup leadership: How intergroup leadership predicts psychological empowerment through intergroup relational identification and resources. *Journal of Applied Social Psychology,* 50(12), 709-719.

van Knippenberg, D. (2020). Meaning-based leadership. *Organizational Psychology Review,* 10(1), 6-28.

van Leeuwen, E.; Harinck, F. (2016). Increasing Intergroup Distinctiveness: The Benefits of Third Party Helping. *Personality and Social Psychology Bulletin,* 42(10), 1402-1415.

van Rekom, J.; van Riel, C.; Wierenga, B. (2006). A methodology for assessing organizational core values. *Journal of Management Studies*, 43(2), 175-201.

Varendh-Mansson, C.; Wry, T.; Szafarz, A. (2020). Anchors Aweigh? Then Time to Head Upstream: Why We Need to Theorize Mission Before Drift. *Academy of Management Review,* 45(1), 230-234.

Veldsman, t.; Veldsman, D. (2020). Critically problematising existing organisational identity theory against practice: Part 1 - The thinking framework of organisational identity. *SA Journal of Industrial Psychology,* 46 (1), 1-12.

Vidal, D.; Pittz, T. (2019). Educating beyond the classroom: alumni giving and the value of campus culture. *Studies in Higher Education,* 44(12), 2208-2222.

Vieru, D.; Rivard, S. (2014). Organizational identity challenges in a post-merger context: A case study of an information system implementation project. *International Journal of Information Management*, 34(3), 381-386.

Vincent, S. (2018). Social memory assets as a defense mechanism: the Onondaga Pottery in World War II*. Management and Organizational History,* 13(4), 352-372.

Vora, D.; Sumelius, J.; Makela, K.; John, S. (2021). Us and them: Disentangling forms of identification in MNCs. *Journal of International Management,* 27(1).

Voss, Z.; Cable, D.; Voss, G. (2006). Organizational identity and firm performance: What happens when leaders disagree about who we are?. *Organization Science*, 17(6), 741-755.

Wadham, H.; Warren, R. (2014). Telling Organizational Tales: The Extended Case Method in Practice. *Organizational Research Methods,* 17(1), 5-22.

Waeraas, A. (2008). Can public sector organizations be coherent corporate brands?.*Marketing Theory*, 8(2), 205-221.

Waeraas, A. (2010). Communicating Identity: The Use of Core Value Statements in Regulative Institutions. *Administration and Society,* 42(5), 526-549.

Waeraas, A. (2014). Beauty From Within: What Bureaucracies Stand for. *American Review of Public Administration*, 44(6), 675-692.

Waeraas, A. (2020). They Put Themselves Out There: A Longitudinal Study of Organizational Expressiveness. *Corporate Reputation Review,* 23(4), 267-279.

Waeraas, A.; Byrkjeflot, H. (2012). Public sector organizations and reputation management: five problems. International Public Management Journal, 15(2), 186-206.

Waeraas, A.; Solbakk, M. (2009). Defining the essence of a university: lessons from higher education branding. *Higher Education*, 57(4), 449-462.

Waldron, T.; Fisher, G.; Pfarrer, M. (2016). How Social Entrepreneurs Facilitate the Adoption of New Industry Practices. *Journal of Management Studies*, 53(5), 821-845.

Waldron, T.; Navis, C.; Fisher, G. (2013). Explaining differences in firms' responses to activism. *Academy of Management Review*, 38(3), 397-417.

Waldron, T.; Navis, C.; Karam, E.; Markman, G. (2022). Toward a Theory of Activist-Driven Responsible Innovation: How Activists Pressure Firms to Adopt More Responsible Practices, *Journal of Management Studies,* 59(1), 163-193.

Walker, B.; Bridgman, T. (2013). Organisational identity and alcohol use among young employees: A case study of a professional services firm. *International Journal of Drug Policy*, 24(6), 597-604.

Walsh, I.; Bartunek, J. (2011). Cheating the fates: organizational foundings in the wake of demise. *Academy of Management Journal*, 54(5), 1017-1044.

Walsh, I.; Pazzaglia, F.; Ergene, E. (2019). Loyal after the end: Understanding organizational identification in the wake of failure. *Human Relations,* 72(2), 163-187.

Walsh, K.; Gordon, J. (2008). Creating an individual work identity. *Human Resource Management Review*, 18(1), 46-61.

Wan, W.; Chen, H.; Yiu, D. (2015). Organizational image, identity, and international divestment: a theoretical examination. *Global Strategy Journal,* 5(3), 205-222.

Wang, H.; Tseng, J.; Yen, Y.; Huang, I. (2011). University staff performance evaluation systems, organizational learning, and organizational identification in taiwan. *Social Behavior and Personality*, 39(1), 43-54.

Wang, T.; Wezel, F.; Forgues, B. (2016). Protecting market identity: when and how do organizations respond to consumers' devaluations?. *Academy of Management Journal*, 59(1), 135-162.

Wang, X. (2022). Marketization in a statist-corporatist nonprofit sector: the case of Hong Kong. *International Review of Administrative Sciences,* 88(2), 449-470.

Wang, Y. (2011). Mission-Driven Organizations in Japan: Management Philosophy and Individual Outcomes. *Journal of Business Ethics*, 101(1), 111-126.

Wang, Z.; Pan, Shan L.; Ouyang, T.; Chou, T. (2014). Achieving IT-Enabled Enterprise Agility in China: An IT Organizational Identity Perspective. *Ieee Transactions On Engineering Management*, 61(1), 182-195.

Wang, Z.; Huang, J.; Tan, B. (2013). Managing organizational identity in the e-commerce industry: An ambidexterity perspective. *Information and Management,* 50(8), 673-683.

Watkins, B.; Smith, S. (2022). I want to work there: how public relations agencies communicate organizational culture and identity on Instagram. *Journal of Communication Management,* 26(1), 58-83.

Watson, A.; Dada, O.; Grunhagen, M.; Wollan, M. (2016). When do franchisors select entrepreneurial franchisees? An organizational identity perspective. *Journal of Business Research*, 69(12), 5934-5945.

Weber, P.; Long, R. (2021). Responding to Change in Higher Education The Case of the Nonprofit Leadership Studies Program at Murray State University and Program Identity. *Journal of Nonprofit Education and Leadership,* 11(1), 54-72.

Wedlin, L. (2007). The role of rankings in codifying a business school template: classifications, diffusion and mediated isomorphism in organizational fields. *European Management Review,* 4(1), 24-39.

Wegner, C.; Jones, G.; Jordan, J. (2019). Voluntary sensemaking: the identity formation process of volunteers in sport organizations. *European Sport Management Quarterly,* 19(5), 625-644.

Weick, K.; Sutcliffe, K.; Obstfeld, D. (2005). Organizing and the process of sensemaking*. Organization Science*, 16(4), 409-421.

Weierter, S. (2001). The organization of charisma: Promoting, creating, and idealizing self. *Organization Studies,* 22(1), 91-115.

Welbourne, T.; Paterson, T. (2017). Advancing a Richer View of Identity at Work: The Role-Based Identity Scale. *Personnel Psychology*, 70(2), 315-356.

Weller, S.; Ran, B. (2020). Social Entrepreneurship: The Logic of Paradox. *Sustainability,* 12(24).

Wenzel, M.; Cornelissen, J.; Koch, J.; Hartmann, M.; Rauch, M. (2020). (Un)Mind the gap: How organizational actors cope with an identity-strategy misalignment. *Strategic Organization,* 18(1), 212-244.

Wessel, L.; Baiyere, A.; Ologeanu-Taddei, R.; Cha, J.; Jensen, T. (2021). Unpacking the Difference Between Digital Transformation and IT-Enabled Organizational Transformation. *Journal of the Association for Information Systems,* 22(1), 102-129.

Westermann-Behaylo, M.; Berman, S.; Van Buren, Harry J., III (2014). The Influence of Institutional Logics on Corporate Responsibility Toward Employees. *Business and Society,* 53(5), 714-746.

Whetten, D. (2006). Albert and Whetten revisited - Strengthening the concept of organizational identity. *Journal of Management Inquiry*, 15(3), 219-234.

Wielsma, A.; Brunninge, O. (2019). Who am I? Who are we? Understanding the impact of family business identity on the development of individual and family identity in business families. *Journal of Family Business Strategy,* 10(1), 38-48.

Wiesenfeld, B.; Raghuram, S.; Garud, R. (1999). Communication patterns as determinants of organizational identification in a virtual organization. *Organization Science*, 10(6), 777-790.

Wightman, G.; Christensen, R.; Sanford, P. (2020). Attitudinal Divergence and Convergence Concerning Collaboration: A Descriptive Case of Public Education in Georgia. *Public Performance & Management Review,* 43(6), 1318-1341.

Wikhamn, B.; Styhre, A. (2017). Open innovation as a facilitator for corporate exploration. International Journal of Innovation Management, 21(6), 1750042.

Williamson, A.; Luke, B.; Fumeaux, C. (2021). Ties That Bind: Public Foundations in Dyadic Partnerships. *Voluntas.* 32(2), 234-246.

Williamson, A.; Luke, B. (2019). Publicness and the Identity of Public Foundations. *Foundation Review,* 11(3), 68-80.

Williamson, A.; Luke, B. (2021). Exploring the accountability and organizational identity of public philanthropic foundations. *International Journal of Management Reviews,* 23(3), 394-410.

Williamson, A.; Luke, B.; Fumeaux, C. (2021). Perceptions and Conceptions of Place in Australian Public Foundations. *Nonprofit and Voluntary Sector Quarterly,* 50(6), 1125-1149.

Withers, M.; Ireland, R.; Miller, D.; Harrison, J.; Boss, D. (2018). Competitive landscape shifts: the influence of strategic entrepreneurship on shifts in market commonality. *Academy of Management Review*, 43(3), 349-370.

Wolfe, A.; Blithe, S. (2015). Managing Image in a Core-Stigmatized Organization: Concealment and Revelation in Nevada's Legal Brothels. *Management Communication Quarterly,* 29(4), 539-563.

Wolter, J.; Bock, D.; Smith, J.; Cronin, J. (2017). Creating Ultimate Customer Loyalty Through Loyalty Conviction and Customer-Company Identification. *Journal of Retailing*, 93(4), 458-476.

Wolter, J.; Landers, V.; Brach, S.; Cronin, J. (2018). Customer-company identification transfer across service alliances. *Journal of Service Management,* 29(1), 98-119.

Worek, M.; De Massis, A.; Wright, M.; Veider, V. (2018). Acquisitions, disclosed goals and firm characteristics: A content analysis of family and nonfamily firms. *Journal of Family Business Strategy*, 9(4), 250-267.

Wright, A.; Zammuto, R. (2013). Creating opportunities for institutional entrepreneurship: The Colonel and the Cup in English County Cricket. *Journal of Business Venturing,* 28(1), 51-68.

Wry, T.; Lounsbury, M.; Glynn, M. (2011). Legitimating nascent collective identities: coordinating cultural entrepreneurship. *Organization Science*, 22(2), 449-463.

Wu, J. (2022). Impact of natural disasters on New Zealand regional family businesses: perspectives of baby boomer family business owners. *Journal of Enterprising Communities,* 16(2), 218-237.

Wu, X. (2022). Internationalization Issues in Chinese Firms: One Belt, One Road-Based Perspective. *Frontiers in Psychology,* 13.

Xie, C.; Bagozzi, R.; Meland, K. (2015). The impact of reputation and identity congruence on employer brand attractiveness. *Marketing Intelligence and Planning,* 33(2), 124-146.

Xiu, L.; Lu, F.; Liang, X. (2019). Legitimized identity vs identifiable legitimacy Toward a theoretical framework of the relationship between organizational identity and organizational legitimacy. *Nankai Business Review International,* 11(1), 102-120.

Xu, J.; Xu, D.; Lu, Y.; Wang, Q. (2018). A bridged government-NGOs relationship in post-earthquake reconstruction: the Ya'an service center in Lushan earthquake*. Natural Hazards*, 90(2), 537-562.

Yakimova, Z.; Tsareva, N.; Vlasenko, A. (2017). Value personnel management: diagnostic tools and development mechanisms. Turkish Online *Journal of Design Art and Communication*, 7 [December Special Edition], 1571-1581.

Yang, H.; Wang, R.; Chen, H. (2021). Professional identity construction among social work agencies. *Journal of Social Work,* 21(4), 753-773.

Yang, T. (2022). Identity development in institutional change of international branch campuses in Malaysia: an empirical study. *Educational Research and Evaluation,* 27(3-4), 309-334.

Yazici, O.; Memili, E.; Patel, P. (2022). Non-family Employees in Family firms and Turnover Intentions: The Relevance of Identification and Justice Perceptions. *Entrepreneurship Research Journal,* 12(2), 107-135.

Yin, X.; Shanley, M. (2008). Industry determinants of the merger versus alliance decision. *Academy of Management Review*, 33(2), 473-491.

Young, J. (2017). Facebook, Twitter, and Blogs: The Adoption and Utilization of Social Media in Nonprofit Human Service Organizations. *Human Service Organizations Management, Leadership and Governance*, 41(1), 44-57.

Younger, S.; Fisher, G. (2020). The exemplar enigma: New venture image formation in an emergent organizational category. *Journal of Business Venturing,* 35(1).

Yu, S.; Zhang, Y.; Yu, J.; Yang, X.; Mardani, A. (2021). The Moderating Impact of Organizational Identity Strength between Strategic Improvisation and Organizational Memory and Their Effects on Competitive Advantage. *Sustainability,* 13(6).

Zachary, M.; McKenny, A.; Short, J.; Davis, K.; Wu, D. (2011). Franchise branding: an organizational identity perspective. *Journal of the Academy of Marketing Science*, 39(4), 629-645.

Zachary, M.; McKenny, A.; Short, J.; Payne, G. (2011). Family Business and Market Orientation: Construct Validation and Comparative Analysis. *Family Business Review*, 24(3), 233-251.

Zagenczyk, T.; Gibney, R.; Few, W.; Scott, K. (2011). Psychological Contracts and Organizational Identification: The Mediating Effect of Perceived Organizational Support. *Journal of Labor Research*, 32(3), 254-281.

Zaman, R.; Jain, T.; Samara, G.; Jamali, D. (2022). Corporate Governance Meets Corporate Social Responsibility: Mapping the Interface. *Business and Society,* 61(3), 690-752.

Zamparini, A.; Lurati, F. (2017). Being different and being the same: Multimodal image projection strategies for a legitimate distinctive identity. *Strategic Organization,* 15(1), 6-39.

Zavyalova, A.; Pfarrer, M.; Reger, R. (2017). Celebrity and infamy? The consequences of media narratives about organizational identity. *Academy of Management Review*, 42(3), 461-480.

Zellweger, T.; Kellermanns, F.; Eddleston, K.; Memili, E. (2012). Building a family firm image: How family firms capitalize on their family ties. *Journal of Family Business Strategy*, 3(4), 239-250.

Zellweger, T.; Nason, R.; Nordqvist, M.; Brush, C. (2013). Why Do Family Firms Strive for Nonfinancial Goals? An organizational identity perspective. *Entrepreneurship: Theory and Practice,* 37(2), 229-248.

Zellweger, T.; Dehlen, T. (2012). Value Is in the Eye of the Owner: Affect Infusion and Socioemotional Wealth Among Family Firm Owners. *Family Business Review*, 25(3), 280-297.

Zellweger, T.; Nason, R.; Nordqvist, M. (2012). From Longevity of Firms to Transgenerational Entrepreneurship of Families: Introducing Family Entrepreneurial Orientation. *Family Business Review*, 25(2), 136-155.

Zhang, H.; Kwan, H.; Everett, A.; Jian, Z. (2012). Servant leadership, organizational identification, and work-to-family enrichment: The moderating role of work climate for sharing family concerns. *Human Resource Management,* 51(5), 747-767.

Zhang, Y.; Biniari, M. (2021). Forging a collective entrepreneurial identity within existing organizations through corporate venturing. *International Journal of Entrepreneurial Behaviour and Research.* 27(6), 1502-1525.

Zhang, Y.; Huxham, C. (2020). Collective identity construction in international collaborations. *Journal of General Management,* 45(3), 123-140.

Zhao, L.; Lee, J.; Moon, S. (2019). Employee response to CSR in China: the moderating effect of collectivism. *Personnel Review,* 48(3), 839-863.

Zhao, W. (2008). Social categories, classification systems, and determinants of wine price in the California and French wine industries. *Sociological Perspectives,* 51(1), 163-199.

Zhu, B. (2019). The Arts and International Relations: The Evolving Identity of the Edinburgh International Festival in the Cold War Era. *Journal of Arts Management Law and Society,* 49(3), 203-218.

Zundel, M.; Holt, R.; Popp, A. (2016). Using history in the creation of organizational identity. *Management and Organizational History,* 11(2), 211-235.

**Appendix 2** *Topic modeling specifications*

The automated textual analysis can be divided into two steps. First, we had to convert all pdf files into txt files to prepare the raw text. We deleted all journal-specific annotations, footnotes, tables, acknowledgments, and references. At the end of this first cleaning step, we had a comparable, machine-readable corpus. After all txt files were adjusted, the authors proofread the changes of one another and imported the files into a python dataframe (pandas library, version 1.0.1). Second, we cleaned our data from all punctuations, stop words (prepositions, pronouns, …) and lowercased everything as well as lemmatized and stemmed the corpus in the end (nltk library, version 3.4.5).

For topic modeling, we used the python library genism (version 3.8.1) and matplotlib (version 3.2.0) to calculate and visualize our results. To gain a topic number that represented all topics without generating too many similar topics, we first calculate a coherence model with a range of 10-120 topics by two topics per step. We choose the first peak of the coherence model resulting in the best fit at 18 topics for our dataset. To ensure that our results are reproducible we fixed a seed for the randomized steps of LDA (seed = 77). Thereafter, we explored the best fitting alpha and observed that the alpha setting auto for gensim provided us with the best readable output. We did the same for eta and decided on the default setting as it gave us the best output.


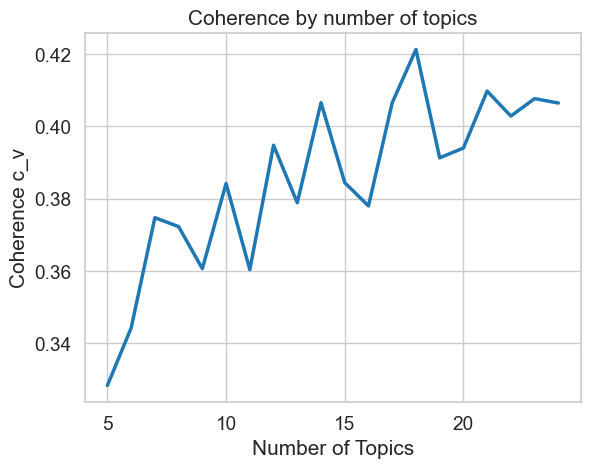


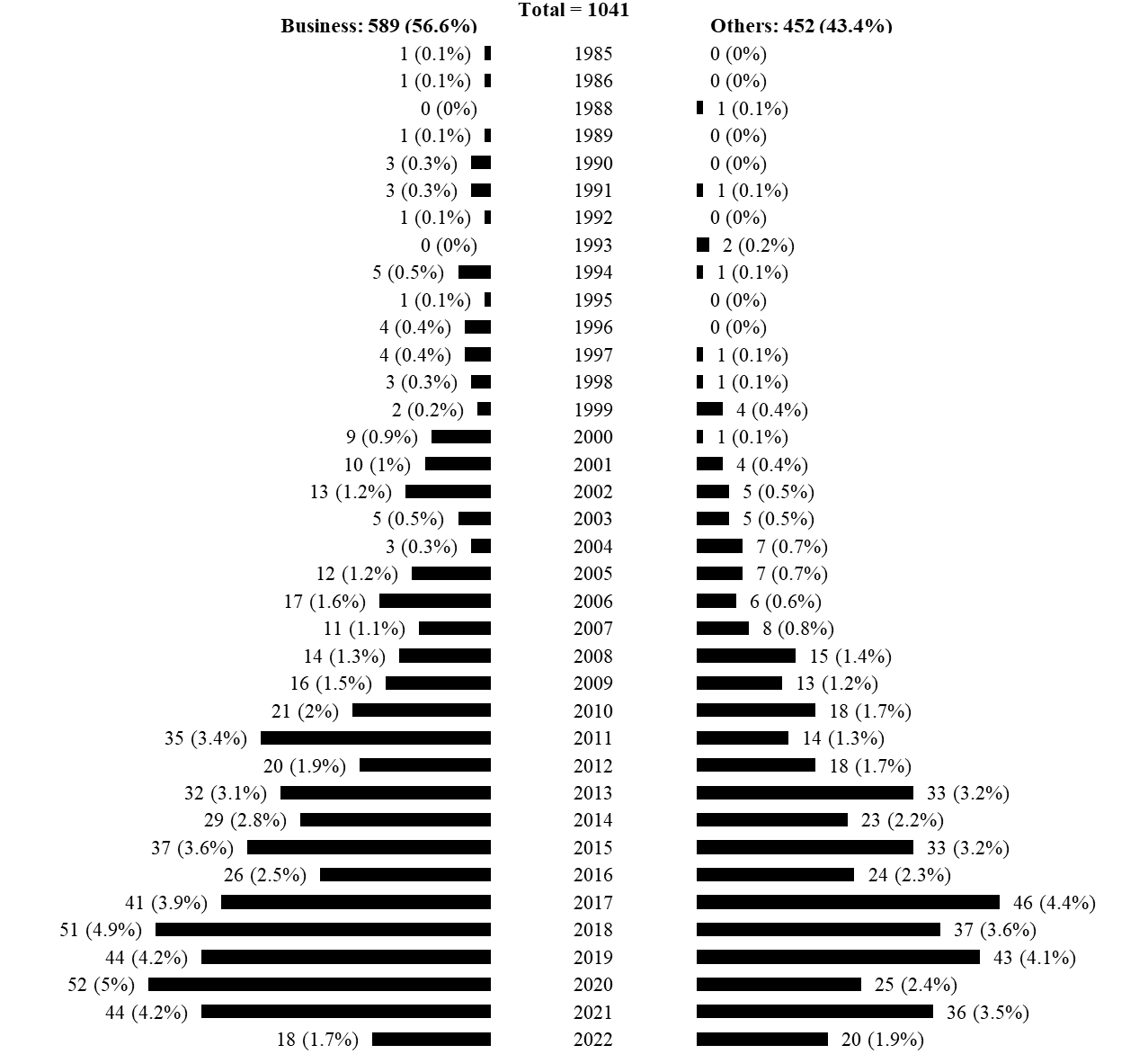
**APPENDIX 3** *Publication per Field by Year (Total of 1,041 Papers)*

**APPENDIX 4** *Frequencies of Top-Tier Journal Publications per Aggregated Dimension*

| **Journal and published aggregated dimensions** | **# of publi-cations^[[1]](#footnote-1)^** | **SCImago Journal Rank** | **Earliest publication** | **Mean year of publi-cation^[[2]](#footnote-2)^** | **Latest publi- cation** |
| --- | --- | --- | --- | --- | --- |
| **Administrative Science Quarterly** | **23** | **17.36** | **1986** | **2008** | **2021** |
| Methodology/ Research approach | 10 | 17.36 | 1998 | 2013 | 2021 |
| Organizational development | 5 | 17.36 | 1996 | 2009 | 2019 |
| Organizational theory research | 6 | 17.36 | 1986 | 2000 | 2011 |
| Strategic management | 2 | 17.36 | 1998 | 2004 | 2009 |
| **Academy of Management Annals** | **9** | **14.78** | **2011** | **2015** | **2018** |
| Organizational development | 3 | 14.78 | 2013 | 2016 | 2018 |
| Organizational theory research | 5 | 14.78 | 2011 | 2015 | 2018 |
| Strategic management | 1 | 14.78 | 2014 | 2014 | 2014 |
| **Academy of Management Journal** | **40** | **10.87** | **1990** | **2011** | **2021** |
| Methodology/ Research approach | 17 | 10.87 | 1991 | 2013 | 2020 |
| Organizational development | 6 | 10.87 | 2006 | 2012 | 2021 |
| Organizational fields | 4 | 10.87 | 2000 | 2009 | 2015 |
| Organizational theory research | 8 | 10.87 | 2005 | 2014 | 2019 |
| Strategic management | 5 | 10.87 | 1990 | 2002 | 2015 |
| **Strategic Management Journal** | **13** | **9.44** | **1991** | **2009** | **2021** |
| Methodology/ Research approach | 2 | 9.44 | 2001 | 2010 | 2019 |
| Organizational development | 5 | 9.44 | 1991 | 2009 | 2019 |
| Organizational fields | 5 | 9.44 | 2000 | 2011 | 2021 |
| Organizational theory research | 1 | 9.44 | 1997 | 1997 | 1997 |
| **Academy of Management Review** | **41** | **7.62** | **1989** | **2011** | **2021** |
| Methodology/ Research approach | 1 | 7.62 | 1990 | 1990 | 1990 |
| Organizational development | 8 | 7.62 | 1994 | 2006 | 2014 |
| Organizational fields | 4 | 7.62 | 2008 | 2014 | 2018 |
| Organizational theory research | 24 | 7.62 | 1989 | 2012 | 2021 |
| Strategic management | 4 | 7.62 | 2008 | 2014 | 2018 |
| **Journal of Marketing** | **6** | **7.46** | **1994** | **2001** | **2012** |
| Methodology/ Research approach | 4 | 7.46 | 1994 | 2002 | 2012 |
| Organizational development | 1 | 7.46 | 1994 | 1994 | 1994 |
| Organizational theory research | 1 | 7.46 | 2003 | 2003 | 2003 |
| **Journal of Management** | **5** | **7.12** | **1991** | **2010** | **2022** |
| Organizational development | 2 | 7.12 | 2001 | 2011 | 2021 |
| Organizational theory research | 2 | 7.12 | 1991 | 2004 | 2017 |
| Strategic management | 1 | 7.12 | 2022 | 2022 | 2022 |
| **Organization Science** | **38** | **6.87** | **1997** | **2009** | **2021** |
| Methodology/ Research approach | 8 | 6.87 | 1997 | 2006 | 2020 |
| Organizational development | 8 | 6.87 | 2000 | 2007 | 2016 |
| Organizational theory research | 19 | 6.87 | 1997 | 2010 | 2021 |
| Strategic management | 3 | 6.87 | 2010 | 2014 | 2018 |
| **Journal of Applied Psychology** | **1** | **6.45** | **2005** | **2005** | **2005** |
| Strategic management | 1 | 6.45 | 2005 | 2005 | 2005 |
| **Journal of Business Venturing** | **4** | **5.82** | **2013** | **2019** | **2022** |
| Organizational development | 2 | 5.82 | 2020 | 2021 | 2022 |
| Organizational theory research | 1 | 5.82 | 2021 | 2021 | 2021 |
| Strategic management | 1 | 5.82 | 2013 | 2013 | 2013 |
| **Management Science** | **1** | **5.09** | **2020** | **2020** | **2020** |
| Methodology/ Research approach | 1 | 5.09 | 2020 | 2020 | 2020 |
| **Personnel Psychology** | **3** | **5.08** | **2012** | **2016** | **2020** |
| Strategic management | 3 | 5.08 | 2012 | 2016 | 2020 |

**APPENDIX 5** *Future Research*

| **Research gap** | **Potential research questions** |
| --- | --- |
| **Evolving character of organizational identity (life-cycles of organizations)** | How can the construction of organizational identity be managed during different and changing life-cycles of the organization?  How does the growth rate of an organization influence the construction of organizational identity? |
| **Metaphor analysis as a methodological approach to organizational identity** | How are metaphors used to describe organizational identity?  What influence do metaphors used for identity construction have on the employees’ or stakeholders’ identification with the organization? |
| **Demand for a better operationalization** | How can organizational identity be operationalized for quantitative analysis? |

1. The total number of journal publications citing Albert and Whetten (1985) including all papers, even those that do not have a dominant dimension. [↑](#footnote-ref-1)
2. Years were rounded up or down to whole numbers. [↑](#footnote-ref-2)
